# A scalable deep-learning framework for cancer detection using cell-free DNA shallow whole-genome sequencing

**DOI:** 10.1126/sciadv.ady9432

**Published:** 2026-07-10

**Authors:** Haichao Wang, Paulius D. Mennea, Grainne McAndrew, Ozge Sonmezler, Dmitry S. Shcherbo, Emma-Jane Ditter, Sarah Østrup Jensen, Alessandra I. G. Buma, Christopher G. Smith, Zhao Cheng, Clare Harris, Rosalind. J. Cutts, Sarah Hrebien, Philip A. J. Crosbie, Pippa G. Corrie, Michel M. van den Heuvel, Amit Roshan, Frank McCaughan, Robert C. Rintoul, Florian Markowetz, Tommy Kaplan, Wendy N. Cooper, Hui Zhao, Nitzan Rosenfeld

**Affiliations:** ^1^ Centre for Cancer Cell and Molecular Biology, Barts Cancer Institute, Queen Mary University of London, John Vane Science Centre, Charterhouse Square, London EC1M 6BQ, UK.; ^2^ Cancer Research UK Cambridge Institute, University of Cambridge, Cambridge CB2 0RE, UK.; ^3^ Cancer Research UK Cambridge Centre, University of Cambridge, Cambridge CB2 0RE, UK.; ^4^ Department of Respiratory Medicine, Radboud University Medical Center, Nijmegen, Netherlands.; ^5^ Victor Philip Dahdaleh Heart and Lung Research Institute, Department of Medicine, University of Cambridge, Cambridge CB2 0BB, UK.; ^6^ Breast Cancer Now Toby Robins Research Centre, The Institute of Cancer Research, London SW3 6JB, UK.; ^7^ Division of Immunology, Immunity to Infection and Respiratory Medicine, Faculty of Biology Medicine and Health, University of Manchester, Manchester M13 9PT, UK.; ^8^ Department of Oncology, Cambridge University Hospitals NHS Trust, Cambridge CB2 0QQ, UK.; ^9^ Department of Respiratory Medicine, University Medical Center Utrecht, Utrecht, Netherlands.; ^10^ Department of Plastic & Reconstructive Surgery, Cambridge University Hospitals NHS Trust, Cambridge CB2 0QQ, UK.; ^11^ Royal Papworth Hospital NHS Foundation Trust, Cambridge CB2 0AY, UK.; ^12^ Department of Oncology, University of Cambridge, Cambridge CB2 0QQ, UK.; ^13^ School of Computer Science and Engineering, The Hebrew University of Jerusalem, Jerusalem, Israel.; ^14^ Department of Developmental Biology and Cancer Research, Faculty of Medicine, The Hebrew University of Jerusalem, Jerusalem, Israel.

## Abstract

Cell-free DNA (cfDNA) in body fluids enables noninvasive cancer detection. Multifeature artificial intelligence (AI) can improve sensitivity by integrating diverse biomarkers when cancer signals are sparse. Tumor-informed assays that rely on mutations have limited practicality for early cancer detection. Emerging fragmentomic and epigenetic features underpin tumor-naive approaches to screening for individuals with low tumor burden. Here, we designed UNITE—a universal cfDNA feature ensemble framework that provides scalable cancer detection methods based on “genomic bin–fragment length” matrices derived from shallow whole-genome sequencing (sWGS) data at 0.1× depth. Using sWGS data from 2063 plasma samples (631 controls and 1432 cases from 26 cancer types), we systematically evaluated both XGBoost (UNITE-XGB) and convolutional neural networks (UNITE-CNN) across multiple feature spaces and cancer stages. In stage I-II cancer, UNITE-XGB and UNITE-CNN achieved 31 and 21% sensitivity, respectively, at 95% specificity. These findings provide roadmaps for developing multifeature AI beyond plasma biopsies.

## Teaser

Multifeature AI detects cancer across all stages with 47% sensitivity at 95% specificity using sWGS data.

## INTRODUCTION

Cell-free DNA (cfDNA) is naturally released into body fluids (e.g., blood, urine, and cerebrospinal fluid) through various biological processes (*1*–*4*). Plasma cfDNA has a modal size of 166 base pairs (bp), which is proposed to reflect DNA wrapped around nucleosomes (around 146 bp) plus the linker DNA between adjacent nucleosomes (around 20 bp) (*5*). These fragments are short-lived (half-life of ~30 min) and carry both genetic and nongenetic information that reflects the physiological state and disease progression of the host (*1*, *2*). As minimally invasive biopsies, cfDNA isolated from peripheral blood has been used in various clinical settings, including noninvasive prenatal testing (*6*), microbial infection monitoring during organ transplant (*7*), and cancer detection (*8*–*12*).

Although mutation-based approaches, such as tumor-informed circulating tumor DNA (ctDNA) assays, have been widely adopted for minimal residual disease detection (*8*–*10*, *13*), their reliance on matched tissue specimens limits their practicality, particularly when tumor tissue is unavailable (e.g., cancer screening in high-risk population). In contrast, tumor-naive cfDNA features, such as fragment length (*14*–*17*), nucleotide context (*18*–*21*), topology (*14*, *22*, *23*), nucleosome footprints (*24*–*29*), and repetitive elements (*30*–*32*), have gained increasing attention for their applicability in detecting cancer without prior tumor information (*14*, *19*, *33*–*37*). Collectively termed fragmentomic features (*34*, *36*, *38*), these characteristics capture the structural and biochemical properties of cfDNA molecules that differ between healthy and cancer-derived fragments.

For example, ctDNA fragments are typically shorter than cfDNA derived from nonmalignant cells (*14*, *37*), and cancer signals can be enriched by selectively analyzing shorter fragments (*15*). Cristiano *et al.* (*17*) quantified this phenomenon by computing short-to-long fragment ratios across genomic bins to infer the presence of cancer. In addition, cfDNA coverage patterns around transcription factor–binding sites and transcription start sites reflect nucleosome positioning and gene activity, providing insights into the underlying cell of origin (*24*–*26*, *28*). The landscape of 5′ end motifs often shows elevated frequencies of A or T nucleotides in ctDNA, reflecting altered nuclease cleavage preferences, (for example, DNASE1 and DNASE1L3), that correlates with methylation status of the fragments (*19*–*21*, *37*, *39*–*41*). Together, these fragmentomic signatures form the basis of tumor-naive, tissue-independent cancer detection strategies (*11*, *12*, *17*, *21*, *27*, *42*–*44*).

In recent years, artificial intelligence (AI) has evolved from a specialized resource into a broadly accessible research tool. Various deep-learning architectures, such as convolution neural networks (CNNs) (*45*–*47*), recurrent neural networks (RNNs), graph neural networks, and transformers (*48*, *49*), have been applied to a broad range of biomedical research topics (*50*). Examples include CNNs in computational pathology (*51*), large language models (LMMs) for predicting phenotypes from DNA sequences (*52*), image-based deep learning and LLM in primary diabetes care (*53*), and RNNs in drug discovery (*54*). In contrast to traditional statistical machine-learning methods [e.g., support vector machine (*55*) and logistic regression (*56*)], the full potential of deep-learning neural networks has not yet been exploited in the field of blood-based cancer detection. Furthermore, it remains unclear how individual features contribute to an AI model’s decision-making in this context.

**Fig. 1.**
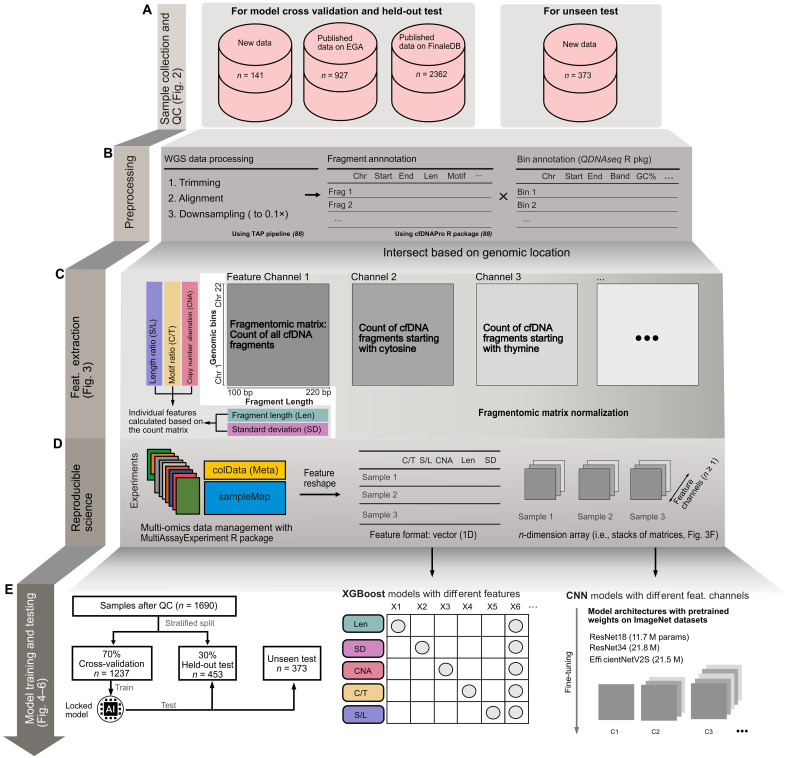
Study overview. (**A**) sWGS data (*n* = 3430) were collected for cross-validation and held-out testing. This includes samples sequenced in this study and published datasets from EGA and FinaleDB (*57*). The numbers shown are before sample filtering (see Fig. 2 for sample selection). An additional independent dataset was collated for the unseen test. (**B**) Sample selection and data preprocessing. (**C**) Feature extraction: (i) Marginal features (i.e., Len, SD of Len counts across 5-Mb genomic bins; and S/L ratio, 5′ C/T motif ratio and CNA in each bin). (ii) Preparation of feature matrices. (**D**) Multi-omics data management using the MultiAssayExperiment R package and feature preparation: Features are arranged into dataframes or tensors as input to XGBoost and CNN models. (**E**) Comparison of the performance of XGBoost and CNN binary classifiers. “Short” and “Long” denote 100 to 150 bp and 151 to 220 bp, respectively; “C” and “T” indicate “cytosine” and “thymine” at the 5′ start positions of fragments, respectively.

To assess the feasibility of multifeature AI for early cancer detection, we collated shallow whole-genome sequencing (sWGS) data from 3430 plasma samples from three sources: new data sequenced for this study, the European Genome-phenome Archive (EGA), and FinaleDB (*57*). After quality control, 1690 samples remained for downstream analysis and were stratified into 1237 cross-validation and 453 held-out test samples. In addition, an independent cohort of 373 plasma samples was assembled as a completely unseen test set for model evaluation (Fig. 1A). sWGS data were converted into vector features and a multidimensional array (Fig. 1, B to D), including fragment length (Len); SD of Len counts (SD) across 5-Mb genomic bins; and short/long length ratio (S/L), 5′ cytosine/thymine motif ratio (C/T), and copy number aberration (CNA) for each bin, were derived. On the basis of this framework, we benchmarked two types of models: XGBoost (using vector features as input) and CNN model (using multidimensional arrays as input) for binary classification (cancer versus healthy). Models were evaluated by repeated cross-validation and then trained using the entire cross-validation set and tested on held-out and unseen test sets (Fig. 1E). These analyses showed that UNITE can sensitively detect inherent cancer signals from cfDNA sWGS data (0.1×), providing a proof-of-principle strategy for cancer screening using multifeature deep-learning approaches.

## RESULTS

### Sample selection

**Fig. 2.**
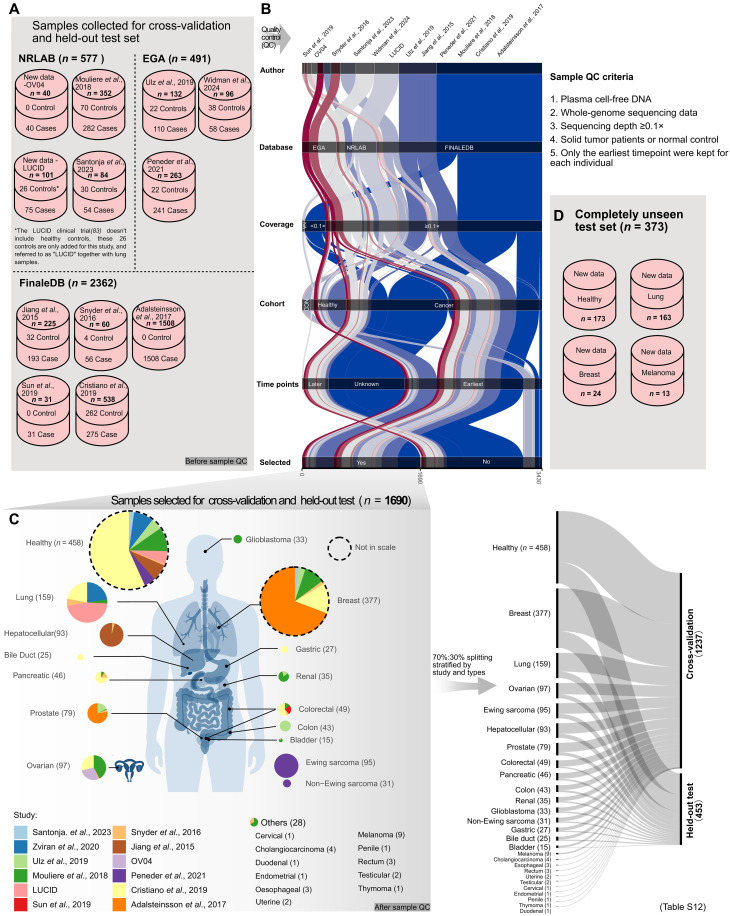
Plasma samples collated in the study. (**A**) Various sWGS data (*n* = 3430) were collected for cross-validation and held-out testing. (**B** and **C**) 1690 of 3430 plasma samples were selected for cross-validation and held-out testing, including healthy donors (*n* = 458) and 26 cancer types (*n* = 1232). The datasets in the top “Author” bar of (B), from left to right: Sun *et al.* (*16*); OV04; Snyder *et al.* (*24*); Santonja *et al.* (*88*); Widman *et al.* (*12*); LUCID; Ulz *et al.* (*26*); Jiang *et al.* (*14*); Peneder *et al.* (*27*); Mouliere *et al.* (*15*); Cristiano *et al.* (*17*). (**D**) Plasma cfDNA sWGS data (*n* = 373) of healthy donors and patients with lung cancer, breast cancer, and melanoma were collated for completely unseen tests of the locked modes.

For the sample pool prepared for cross-validation and held-out testing, we selected 1690 of 3430 plasma samples (from 26 cancer types) across various studies for downstream analysis (Fig. 2, A and B). Only samples meeting the following criteria were included: (i) plasma cfDNA, (ii) obtained from patients with cancer or healthy donors, (iii) WGS with sequencing depth ≥0.1×, and (iv) only the earliest time point selected if multiple specimens are collected from the same individual. The three largest classes are healthy (*n* = 458), breast cancer (*n* = 377), and lung cancer (*n* = 159) (Fig. 2C). In addition, we assembled a completely independent unseen test set (*n* = 373), which includes sWGS data of plasma samples from healthy donors (*n* = 173), lung cancer (*n* = 163), breast cancer (*n* = 24), and patients with melanoma (*n* = 13) (Fig. 2D).

### Vector features extracted for the XGBoost model and arrays for the CNN model differ between case and control samples

**Fig. 3.**
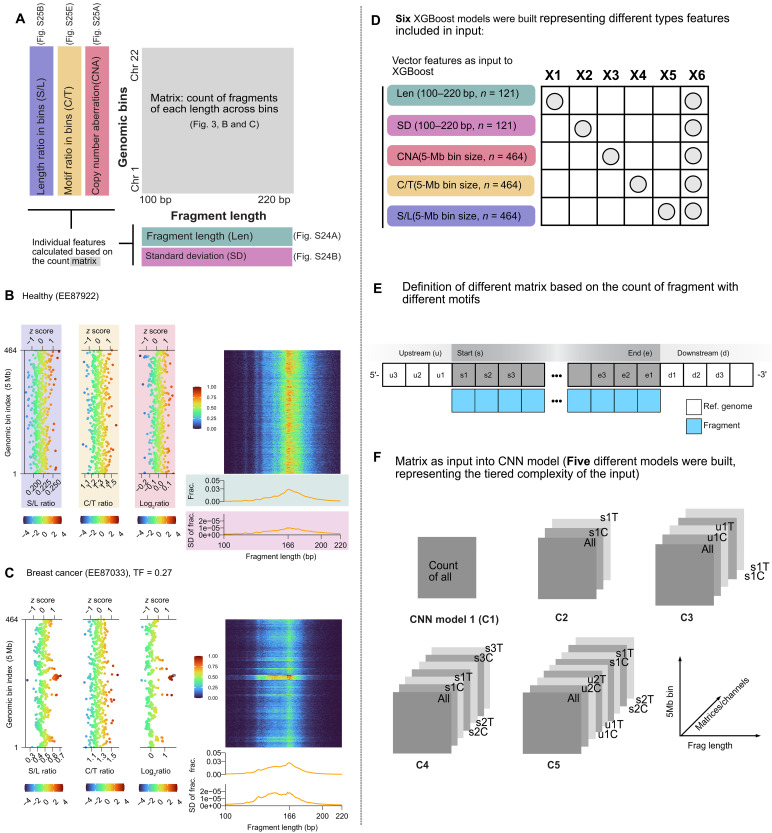
Features used and models built. (**A**) Definition of the fragmentomic matrix and its marginal features. (**B**) A fragmentomic matrix and its marginal features derived from a healthy individual. (**C**) A fragmentomic matrix and its marginal features derived from a patient with breast cancer. (**D**) XGBoost models (X1 to X6) constructed using different feature combinations. Additional combinations of individual features are shown in fig. S3. (**E**) Definition of different motif categories at various positions relative to fragment alignment to the reference genome. The letters u, s, e, and d denote four motifs categorized on the basis of the upstream, start, end, and downstream nucleotides, respectively. (**F**) CNN models (C1 to C5) built using various feature channels. For example, channel s1C represents a matrix of fragment counts starting with cytosine.

For each sample, we conceptualize a “genomic bin–Len” matrix, referred to as the fragmentomic matrix. Columns (*n* = 121) represent Len from 100 to 220 bp (1-bp increments), rows (*n* = 464) correspond to nonoverlapping 5-Mb genomic bins. Matrix values represent fragment counts scaled between 0 and 1. On the basis of this matrix, we derived five marginal features: Len, calculated as the sum of fragment counts across all bins; the SD; and three bin-wise metrics used to assess the relationship between fragmentomic features and CNAs, including the S/L, defined as the ratio of short (100 to 150 bp) to long (150 to 220 bp) fragments in each bin (*17*); the C/T, representing the ratio of C- to T-starting 5′ end motifs in each bin; and the GC-corrected, self-normalized, and segmented log_2_ ratio (CNA) (Fig. 3A) (*17*).

For illustration, Fig. 3 (B and C) shows the raw matrix (heatmap) and marginal features of a healthy and a breast cancer sample [female, tumor fraction (TF) = 0.27], respectively. Red regions on the heatmap indicate a higher count (i.e., copy number gains). In general, these signals are reflected in row-wise marginal features such as S/L, C/T, and CNA. However, as shown in Fig. 3C, cancer-associated signals are not always linearly represented on the heatmap, and such patterns cannot be fully captured by marginal features alone. This strongly supports the need for a deep-learning model capable of detecting nonlinear patterns for improved sensitivity.

To quantify and compare the biological feature dynamics across different tumor burdens or cancer stages, we stratified the samples into five categories based on the TF estimated using ichorCNA (fig. S23A) (*58*). Healthy controls (*n* = 458) were assigned to the “Healthy” category. Tumor samples were assigned to TF categories of [0, 0.03] (*n* = 736), (0.03, 0.1] (*n* = 239), (0.1, 0.2] (*n* = 121), and (0.2, 1] (*n* = 136) (fig. S23B). We observed that with increasing TF, cancer samples displayed a progressively larger fraction of fragments below 150 bp (figs. S17 and S24A). All four TF categories showed significantly higher counts of 100- to 150-bp fragments compared with healthy controls (*P* ≤ 0.0001; fig. S24D), and their medians were hierarchically elevated (fig. S24C). We further hypothesized that fragment counts within genomic bins would be more variable in the cancer samples because of the copy-number or chromatin-status changes. SD was calculated for each Len (i.e., step size of 1 bp). As expected, cancer samples displayed higher variability than healthy controls (fig. S24, B and E), and the sum of SD values for 100- to 150-bp fragments differed significantly across TF categories compared with healthy controls (fig. S24F).

CNA profiles exhibited increasing heterogeneity with higher TF (fig. S25, A and D). We calculated the correlation between each sample’s S/L profile and the healthy median (the median profile of all healthy samples). Correlation decreased progressively with increasing TF (fig. S25, B and C).

We performed the same analysis for the C/T and observed consistent trends (fig. S25, E and F). Beyond bin-wise C/T measurements, we also calculated whole-genome level motif ratios. Previous studies have shown that samples from healthy individuals exhibit a higher proportion of CCC motifs (*18*, *37*), and changes in the relative abundance of CGN and NCG motifs at 5′ ends of fragments (N represents any nucleotide) can reflect CpG methylation status (*19*). Thus, we calculated CCC/AAA and CGN/NCG motif ratio and assessed their correlation with the healthy median. Both ratios showed increasingly negative correlations with rising TF (figs. S25G and S18E). These findings are consistent with prior reports (*18*–*21*, *37*, *40*).

### Cross-validation results indicate that multifeature integration improves detection sensitivity

**Fig. 4.**
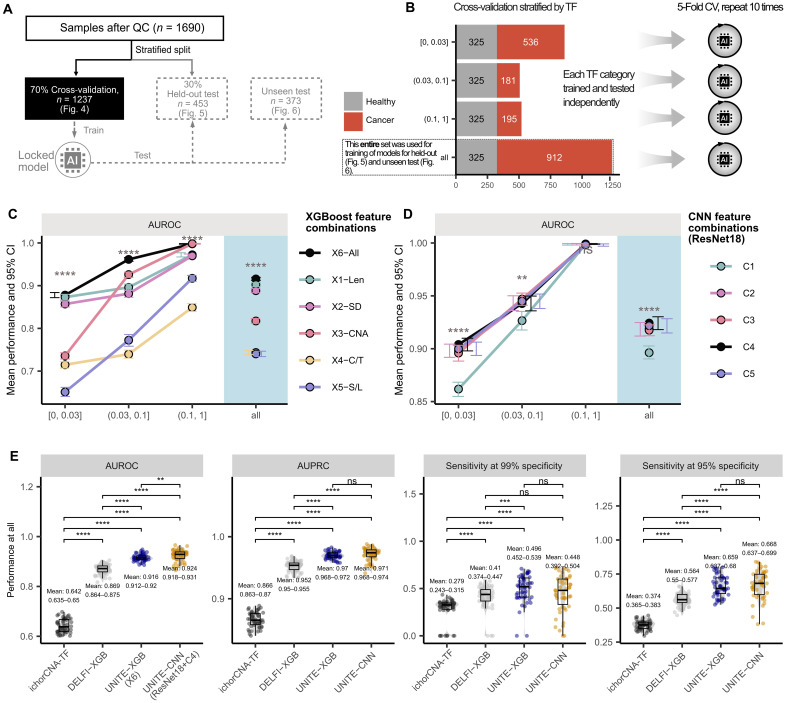
Model performances during cross-validation. (**A**) Sample splitting strategy. (**B**) Samples were stratified into four categories based on ichorCNA-inferred TF to evaluate model performance across TF levels. Only models trained on the unstratified full dataset were used for the held-out test (Fig. 5) and the unseen test (Fig. 6). (**C**) Comparison of XGBoost model performance. The X6 model, which uses all features as input, achieved the highest AUC. (**D**) Comparison of CNN model performance. The C1 model shows suboptimal AUC, whereas multichannel models (C2 to C5) demonstrated improved performance (**E**) Model performance across cross-validation folds without TF stratification (all). Statistical comparisons were performed using the Kruskal-Wallis test. Source data are provided as data file S2. ns, not significant; **P ≤ 0.01; ***P ≤ 0.001; ****P ≤ 0.0001.

To assess the model robustness when trained on samples with different TF levels, we trained and tested XGBoost and CNN models using samples from four different TF categories independently: “[0, 0.03],” “(0.03, 0.1],” “(0.1, 1]”, and “all” (i.e., “[0, 1]”) (Fig. 4B, and fig. S2, A and B). For each cancer type and TF category, the samples were split into 70% training and 30% testing (fig. S2C), which is consistent with the overall data splitting strategy (Fig. 4A). For example, the [0, 0.03] category resulted in 861 training samples (325 healthy and 536 cancer) and 333 testing samples (133 healthy and 200 cancer). The all category included the entire dataset, with 1237 samples (325 healthy and 912 cancer) for training and 453 samples (133 healthy and 320 cancer) for testing (Fig. 4B). To evaluate the relative contributions of different biological features across TF categories, we trained XGBoost models using both individual features and ensemble feature sets (Fig. 3D and fig. S3, B to E).

We found that (i) the ensemble model (the X6 model including all features, hereafter referred to as “UNITE-XGB”) consistently achieved the highest area under the receiver operating characteristic curve (AUC) across all TF categories (Fig. 4C); (ii) for samples with TF below 3%, the ensemble model achieved a mean AUC of 0.878 [95% confidence interval (CI): 0.872 to 0.884], followed by the model using only Len (0.873, 0.867 to 0.879), SD (0.857, 0.851 to 0.863), CNA (0.736, 0.728 to 0.743), C/T (0.715, 0.707 to 0.722), and S/L (0.651, 0.640 to 0.663); (iii) for samples with TF above 3%, CNA became the strongest individual feature, followed by Len, SD, S/L, and C/T; (iv) notably, the S/L outperformed C/T in the (0.03, 0.1] and (0.1, 1] categories (Fig. 4C). Feature strength rankings were consistent across evaluation metrics, including area under precision-recall curve (AUPRC), sensitivity, specificity, F1 score, and accuracy (fig. S3A). Raw performance data and statistical outputs are provided in the data file S1.

We quantified the feature importance with Shapley additive explanations (SHAPs) (*59*), averaged across fivefold cross-validation (10 repeats) in the UNITE-XGB model. The top 30 features are shown in figs. S5 and S6. For samples with ≤3% TF, Len, and SD contributed 8 of the top 10 most important features, consistent with the performance ranking of models X1-X5 in cross-validation (Fig. 4C). The top three features were the fraction of fragments at 105 bp, the copy number of Chr 9 (first bin of p-arm), and the fraction of 104-bp fragments (fig. S5A).

For samples with TF >3%, copy number–derived features became dominant contributors to model predictions. In the (0.03, 0.1] category, 9 of the top 10 features were CNA features, with the top 3 being the copy number of Chr 8 (11th and 9th bin of the q-arm), and Chr 13 (first bin of the q-arm) (fig. S5B). In the [0.1, 1] category, 8 of the top 10 features were CNA features, with the top 3 being the copy number of Chr 8 (15th bin of the q-arm), the fraction of 150-bp fragments, and Chr 8 (13th bin of the q-arm) (fig. S6A). For the all category, 6 of the top 10 features originated from Len features, followed by 2 SD features and 2 CNA features. The top three features for this category were the SD at 150 bp, the fraction of 105-bp fragments, and the copy number of Chr 9 (first bin of the p-arm) (fig. S6B).

### CNNs detects cancer-associated signals directly from fragmentomic matrices

**Fig. 5.**
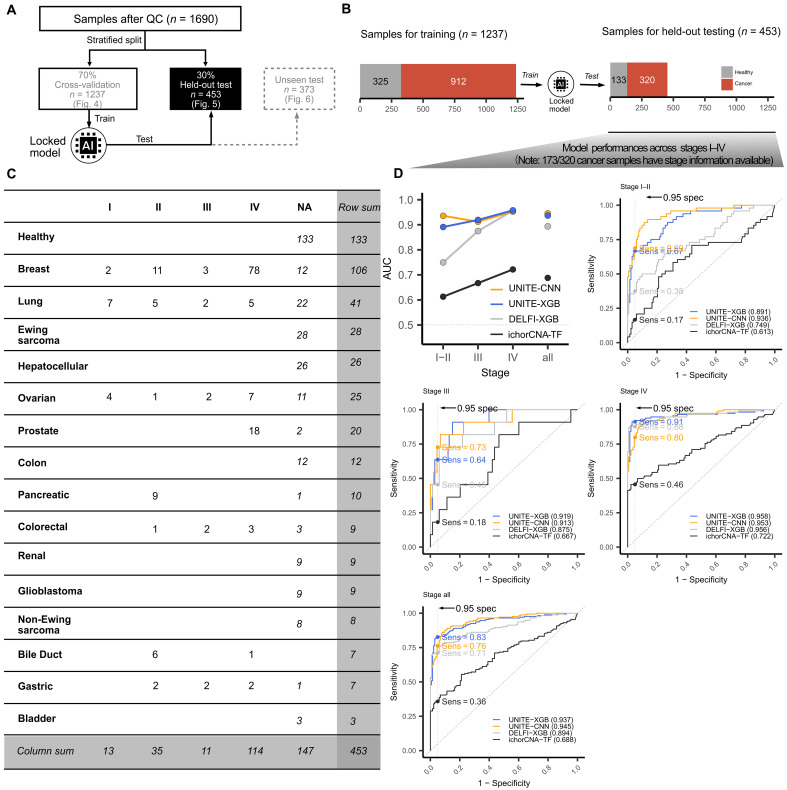
The performance of the locked UNITE models on the held-out test set. (**A** and **B**) Models were trained on the entire cross-validation dataset and subsequently tested on the held-out dataset. (**C**) Number of samples from different cancer types across stages in the held-out test set. NA indicates nonapplicable or not available. (**D**) Performance of the models on the held-out test set. Vertical gray dashed lines indicate 95% specificity. Source data are provided as data file S2.

**Fig. 6.**
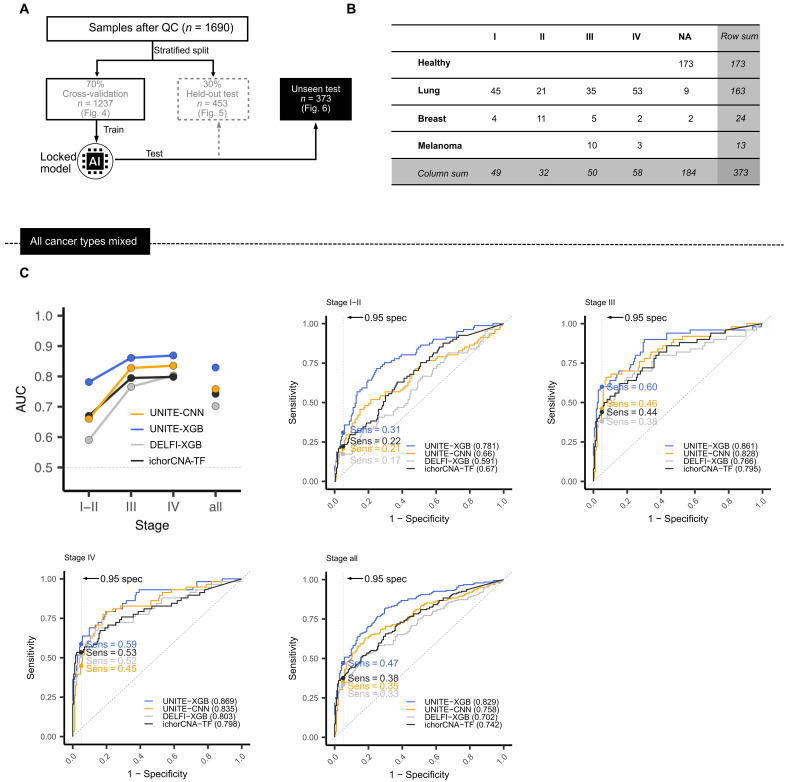
Model comparison in the unseen test set. (**A**) Data splitting and model testing overview schema. (**B**) Cancer types and sample sizes in the unseen test set. (**C**) AUC curves for the UNITE-XGB, UNITE-CNN, ichorCNA-TF, and DELFI-XGB models across cancer stages. Stage I and II were merged to balance the sample sizes across groups. Vertical gray dashed lines indicate 95% specificity. Source data are provided as data file S2.

We hypothesized that cancer-associated signals are inherently represented in the fragmentomic matrix and that CNN architectures can extract these abnormal patterns. We applied multiple CNN architectures: ResNet18 (*47*), ResNet34 (*47*), and EfficientNetV2S (*60*) with 11.7, 21.8, and 21.5 million parameters, respectively. We designed models C1 to C5, each incorporating an increasing number of feature channels (Fig. 3, E and F) to capture different combinations of signals across the expanded feature spaces. The entire matrix (i.e., all feature channels stacked together) was used as input. Models were trained using a fine-tuning strategy based on pretrained ImageNet weights (more than 10 million images from 1000 classes; https://image-net.org/). We found that CNNs successfully detect cancer signals from the matrices (fig. S7). Among the architectures, ResNet18 achieved the highest AUC. For example, in the [0, 0.03] TF category, ResNet18-C4 achieved a mean AUC of 0.904 (95% CI: 0.898 to 0.91) (figs. S36B and S4). While model C1 exhibited the lowest performance, models C2 to C5 achieved similarly strong AUC values within each TF category (Fig. 4D). ResNet18-C4, hereafter referred to as UNITE-CNN, was selected as the optimal architecture for held-out sample evaluation (Fig. 5) and unseen data testing (Fig. 6).

### Benchmarking UNITE-XGB and UNITE-CNN against state-of-the-art models for ctDNA detection

To compare UNITE-XGB and UNITE-CNN with previously published state-of-the-art approaches, we implemented two other models: (i) ichorCNA-TF, which uses TF inferred by ichorCNA (*58*) as input to a logistic regression binary classifier; (ii) DELFI-XGB, which uses the same features as the DELFI study (*17*) as input to an XGBoost binary classifier.

Both models were trained and tested following the same workflow as the UNITE models (Fig. 4A). Across the entire cross-validation dataset without TF stratification (i.e., all), UNITE-CNN achieved the highest performance, with an AUC of 0.924 (95% CI: 0.918 to 0.931), followed by UNITE-XGB (0.916, 95% CI: 0.912 to 0.920), DELFI-XGB (0.869, 95% CI: 0.864 to 0.875), and ichorCNA-TF (0.642, 95% CI: 0.635 to 0.650) (Fig. 4E). These correspond to sensitivities at 95% specificity of 66.8% (CI: 63.7 to 69.9%), 65.9% (CI: 63.7 to 68.0%), 56.4% (CI: 55.0 to 57.7%), and 37.4% (CI: 36.5 to 38.3%), respectively (Fig. 4E). Model comparisons within each TF category are shown in figs. S36A and S8. In addition, model performances were also stratified by cancer stages, which are shown in fig. S36C. As expected, AUCs increased with rising TF. Of note, ichorCNA-TF exhibited a marked increase from 0.603 (CI: 0.595 to 0.611) in the [0, 0.03] category to 0.983 (CI: 0.981 to 0.985) in the (0.03, 0.1] category. In the following analyses, models trained using the entire cross-validation set were referred to as locked models for testing on the held-out and unseen test sets.

### UNITE-CNN outperforms other models in the held-out test set

We evaluated model performance using held-out samples (*n* = 453; Fig. 5, A and B). To provide clinically meaningful interpretation, we examined performance across cancer stages I to IV (Fig. 5, C and D). Stage information was available for 173 of 320 cancer samples. Consistent with the cross-validation results shown in Fig. 4, the UNITE-CNN model achieved the best performance compared to the other models (Fig. 5D).

In this held-out test set, among stage I-II cancers, UNITE-CNN achieved an AUC of 0.936, followed by UNITE-XGB (0.891), DELFI-XGB (0.749), and ichorCNA-TF (0.613). At 95% specificity, the corresponding sensitivities were 69, 67, 38, and 17%, respectively. In stage III cancers, the AUCs were 0.913, 0.919, 0.875, and 0.667, respectively, with sensitivity at 95% specificity of 73, 64, 45, and 18%, respectively. In stage IV cancers, UNITE-XGB had a marginally higher AUC of 0.958, followed by DELFI-XGB (0.956), UNITE-CNN (0.953), and ichorCNA TF (0.722), with sensitivities at 95% specificity of 91, 88, 80, and 46%, respectively. When combining all stages, UNITE-CNN achieved an AUC of 0.945, while UNITE-XGB, DELFI-XGB, and ichorCNA-TF achieved AUCs of 0.937, 0.894, and 0.688, respectively. These correspond to sensitivities of 76, 83, 71, and 36% at 95% specificity, respectively (Fig. 5D).

In addition to stratification by stages shown in Fig. 5, we also performed stratification by ichorCNA TF as this allows more samples to be included in the benchmarking. For the held-out test (fig. S9A), results were consistent: UNITE-CNN was 0.946, compared to 0.944 for UNITE-XGB (fig. S9B). The results were further supported by Venn diagram comparison and individual prediction score distributions (fig. S9, C to E). Additional comparisons across various TF stratified categories are provided in the Supplementary Materials (figs. S8, S10, S11, and S12).

### UNITE-XGB outperforms other models tested using the unseen test set

To independently evaluate the models trained using the entire cross-validation dataset, we tested them on a completely unseen test set (Fig. 6A), which comprised healthy control samples (*n* = 173) and samples from patients with lung cancer (*n* = 163), breast cancer (*n* = 25), and melanoma cancer (*n* = 13) (Fig. 6B). In stages I-II, III, IV, and unstratified all stages, UNITE-XGB consistently showed better performances than other models with AUC of 0.781, 0.861, 0.869, and 0.829, respectively. By fixing the specificity at 95%, UNITE-XGB achieved sensitivity of 31, 60, 59, and 47% in stages I-II, III, IV, and unstratified all stages, respectively (Fig. 6C). In stage I-II cancers, UNITE-CNN and ichorCNA-TF showed comparable AUCs, while UNITE-CNN surpassed ichorCNA-TF in stage III and IV cancers. DELFI-XGB yielded an AUC of 0.591 in stage I-II cancers, lower than UNITE-XGB (0.781), UNITE-CNN (0.66), and ichorCNA-TF (0.67). However, in stage III and IV cancers, DELFI-XGB and ichorCNA-TF achieved comparable AUCs (Fig. 6C).

More specifically, in stage I-II cancers, UNITE-XGB, UNITE-CNN, ichorCNA-TF, and DELFI-XGB achieved AUCs of 0.781, 0.66, 0.67, and 0.591, respectively, corresponding to sensitivities of 31, 21, 22, and 17% at 95% specificity. In stage III cancers, UNITE-XGB and UNITE-CNN outperformed DELFI-XGB and ichorCNA-TF, with AUCs of 0.861, 0.828, 0.766, and 0.795, and sensitivities at 95% specificity of 60, 46, 38, and 44%, respectively. In stage IV cancers, UNITE-XGB achieved an AUC of 0.869, followed by UNITE-CNN (0.835), DELFI-XGB (0.803), and ichorCNA TF (0.798).

When evaluated on the entire unseen test set including all stages, UNITE-XGB demonstrated the highest overall AUC (0.829), outperforming UNITE-CNN (0.758), ichorCNA-TF (0.742), and DELFI-XGB (0.702), corresponding to sensitivities of 47, 35, 38, and 33% at 95% specificity, respectively. Performance across cancer types is shown in figs. S27 and S28.

To access robustness, we challenged the models in different ways. First, we removed lung or breast cancer samples from the training set and then tested performance on the corresponding cancer types from the unseen test set. UNITE-XGB remained robust, with lung-cancer detection sensitivity of 39% before removal and 38% after removal (fig. S29, A and B). Similarly, UNITE-XGB showed a sensitivity of 73 and 68% in the unseen breast test before and after removing breast cancer samples in the training (fig. S29, C and D). In contrast, UNITE-CNN performance degraded when specific cancer types were removed from the training set. For example, the sensitivity for lung cancer dropped from 28 to 17% after removing lung cancer samples during training (fig. S29, A and B). Feature importance analyses showed shifts in rankings but no systematic pattern induced by removing a specific cancer type from the training set (figs. S30 and S31).

Second, we trained models using either all samples in the cross-validation set or only samples with TF ≤3% and tested both on the entire unseen test set. Results indicated that TF distribution did not affect performance, except for the ichorCNA-TF logistic regression model, which failed as expected (fig. S29, E and F). In addition, to estimate the minimum sample size required for effective training, we conducted an ablation study by progressively reducing the number of training samples. Model performance generally increased with larger sample sizes and a sharp decline in AUC occurred when fewer than 150 samples were used (fig. S32). In addition, we also tested the trained model using test set with various sequencing depths (i.e., 0.3×, 0.5×, 1.0×, and 1.5×), results indicate that UNITE-XGB is not sensitive to changes in depth, while UNITE-CNN model requires consistency between training and testing samples (fig. S38).

To access the clinical applicability of UNITE as a complementary tool to standard-of-care cancer screening (fig. S33), we benchmarked its performance on the unseen test set against the performances of various models reported in publications using different features, e.g., mutations, methylations, and fragmentomics. At 99.6% specificity, UNITE-XGB showed 22% sensitivity across all unseen cancer samples (fig. S34). At 98% specificity, UNITE-XGB achieved 19% sensitivity in stage I-II cancers, comparable to or exceeding other models such as Lung CLiP (20 to 29%) (*61*) and DELFI (16 to 17%) (fig. S35) (*62*). The mode performances at various cutoff of specificity is also directly compared in fig. S37. Given the scalability of the UNITE framework and its low sequencing depth requirements, UNITE demonstrates promising utility for translational implementation in cancer early detection settings.

## DISCUSSION

Technical advances have enabled multimodal characterization of cfDNA fragmentomics (*14*, *19*, *33*–*37*), proteomics (*63*), methylation (*41*, *64*, *65*), mutation (*11*, *12*, *66*–*68*), microbes (*7*, *69*), and copy number (*17*, *70*–*75*). This unlocks the door to studying cfDNA across different biochemical dimensions. The field has seen an increasing number of studies integrating multimodal features into AI models to improve the sensitivity of blood-based cancer detection (*11*, *17*, *21*, *26*, *27*, *76*).

However, it remains unclear to what extent different biological features contribute to an AI model’s decision-making across TFs. Furthermore, a gap persists between the rapid development of deep-learning techniques (e.g., neural networks) and their applications to cfDNA analysis. Our study advances the cfDNA field in several ways. Beyond widely studied features such as Len, S/L (*17*, *62*), and CNA, we introduced two more features: C/T and SD, which exhibit heterogeneous patterns between healthy individuals and patients with cancer. We systematically evaluate the importance and contribution of these features toward more sensitive cancer detection. Among patients with ≤3% TF, Len (AUC = 0.873) and SD (0.857) outperformed CNA (0.736), C/T motif ratio (0.715), and the S/L length ratio (0.651).

In settings where sample size limits the use of large-scale deep-learning models, sWGS offers a cost-effective, standardized, and widely accessible method compared with alternatives such as methylome profiling or deep WGS. UNITE is a versatile framework designed primarily for sWGS data, but readily extendable to other data types, to detect cancer. Using only 0.1× sWGS data, UNITE-XGB demonstrated robust and dominant performance, achieving an overall sensitivity of 47 at 95% specificity (31% sensitivity at 95% specificity in stage I-II cancers).

Current cfDNA-based screening assays face a common challenge: moderate sensitivity (typically 25 to 40% in asymptomatic population and lower in early-stage disease) alongside high specificity (usually >99%) (*77*–*79*). This yields low false-positive rates and thus acceptable positive predictive value (PPV). By fixing at a very high specificity of 99.6%, UNITE-XGB achieved 22% sensitivity in the unseen test, which is higher than other models benchmarked in this study. Of note, the model was trained using only 1237 samples, considering development costs and implementation feasibility, UNITE-XGB represents a competitive and potentially more accessible cancer early detection assay, trading a modest degree of sensitivity for improved scalability.

UNITE is highly scalable. Feature channels can be easily expanded (e.g., to incorporate mutations), enabling seamless integration into larger multimodal feature spaces. The MultiAssayExperiment-based feature management ensures reproducibility and provides a structured framework for future cfDNA studies. The datasets collated here form an important reference resource to benchmark emerging liquid biopsy biomarkers. Unlike tissue sequencing from solid cancer, fragmentation profiles are unique to cfDNA. UNITE extends these signals into a two-dimensional representation. This framework supports the development of features and AI models. For example, we introduced the SD feature, which captures disturbances in Len profiles driven by copy number or chromatin-status changes across the genome. Leveraging CNN architectures pretrained on tens of millions of ImageNet images, UNITE provides a versatile framework for multifeature integration and achieves optimized sensitivity using shallow-depth sequencing data for cancer detection.

Despite these findings, several limitations warrant consideration. First, the minimum TF detected by UNITE—particularly in patients with stage I-II cancer—cannot be fully assessed because of the absence of experimentally validated plasma variant allele frequency measurements. The model was not evaluated for its ability to quantify longitudinal TF changes or track disease dynamics. In addition, although pretrained CNNs offer practical advantages given dataset size and computational constraints, their training on natural images may limit domain-specific feature learning. Furthermore, preanalytical variability (e.g., different protocols with various collection tubes and freeze-defrost cycles) and clonal hematopoiesis can affect cfDNA fragmentomic features. In conclusion, the systematic evaluation of diverse biological features, development of the UNITE deep-learning framework, and the assembly of large-scale multicenter datasets collectively advance the frontier of cfDNA-based early cancer detection.

## MATERIALS AND METHODS

### Experimental design

This study aimed to propose a framework (i.e., UNITE) and identify biomarkers derived from this framework to detect cancer signals from plasma cfDNA in a tumor-naive way. WGS was performed to generate sWGS data from lung cancer (LUCID) and ovarian cancer (OV04) plasma samples (Fig. 2A), and from healthy individuals, lung, breast, and melanoma plasma samples as the unseen test set (Fig. 2D). In addition, we collected data from previously published datasets deposited in various databases, e.g., EGA and FinaleDB (*57*). CNN and XGBoost models were built and tested using these data. Model performance and feature importance (e.g., Len and SD) across different cancer types and TFs [inferred using ichorCNA (*58*)] were comprehensively benchmarked.

### Sample collection, sequencing and alignment

cfDNA samples used to generate new sWGS data were derived from the following sources: (i) Ov04: Plasma collected from ovarian cancer patients enrolled in the OV04 study (CTCR-OV04; REC: 08/H0306/61) at Addenbrooke’s Hospital (Cambridge, UK) (*80*, *81*) and (ii) LUCID: Plasma collected from patients with lung cancer enrolled in the LUCID (*82*) study (REC: 14/WM/1072) at Papworth Hospital or Addenbrooke’s Hospital (Cambridge, UK).

Newly sequenced data in the unseen test set were derived from additional studies with appropriate ethical approvals: Melanoma (MELR): (REC: 11/NE/0312, “MelResist: investigating resistance to molecular-targeted melanoma therapies”). Lung cancer: MISIL1 REC: 20/PR/0622 (London - London Bridge Research Ethics Committee); MOD-LUC: 21/WM/0024 (Health and Care Research Wales); a subset of the LEMA cohort (*83*): REC: NL54778.031.15, ClinicalTrials.gov: NCT02894853 (Medical Ethics Committee of the Netherlands Cancer Institute); REC: 18/EE/0269 (East of England–Cambridge East Research Ethics Committee). Control: REC: 19/LO/1477 (London-Surrey Borders Research Ethics Committee). Breast cancer: Neo-RT (*84*): REC: 17/EE/0176 (Cambridge South Research Ethics Committee); and samples from ABC-Bio (*85*) and ChemoNEAR (*86*) studies. Informed consent was obtained from all study participants, and all experiments conformed to the principles of the World Medical Association Declaration of Helsinki and the Department of Health and Human Services Belmont Report. The detailed number of samples from controls and cancer types in cross-validation, held-out test, and unseen test sets are described in table S12.

CfDNA was extracted from plasma using the QIAamp circulating nucleic acid kit (QIAGEN) and sequenced (paired-end 150-bp sWGS). The library preparation kit and sequencer used for the samples are recorded in table S13. Sequencing services were supplied by the Genomics Core Facility at the Department of Cancer Research UK Cambridge Institute, School of Clinical Medicine, University of Cambridge. Data trimming, alignment, and duplication marking was performed using the internal, published Trim Align Pipeline (TAP) (github.com/nrlab-CRUK/TAP) (*87*). Information on extraction kit, library preparation kit, and sequencing platform are available in tables S2 and S13. All resulting bam files were downsampled to 1 million fragments using cfDNAPro (https://github.com/NRLAB-CRUK/cfDNAPro). Version number of tools, software, and R packages used in the analyses are summarized in table S9.

### EGA and FinaleDB data retrieval and processing

Datasets from Mouliere *et al.* (*15*), Santonja *et al.* (*88*), Ulz *et al.* (*26*), Widman *et al.* (*12*), and Peneder *et al.* (*27*) were obtained from EGA. If data holders provided raw data as BAM files, they were converted to FASTQ format first and subsequently processed using the Trim and Align Pipeline (github.com/nrlab-CRUK/TAP). In addition, data from Jiang *et al.* (*14*), Cristiano *et al.* (*17*), Sun *et al.* (*16*), Snyder *et al.* (*24*), and Adalsteinsson *et al.* (*58*) were downloaded directly from FinaleDB (*57*) (http://finaledb.research.cchmc.org). The raw data were provided as “fragment profiles” (TSV files containing fragment alignment coordinates). Fragment profiles were converted into GRanges object in R using plyranges (see Supplementary Materials for more details) (*89*).

### Sample meta-information

For each specimen, meta-information was manually curated from various sources (e.g., collaborators, published articles, EGA records, or FinaleDB). Detailed description of meta-variables associated with each sample is provided in tables S1, S2, and S13. The ichorCNA-derived TF of each sample across stage categories, stratified by dataset and the corresponding sample numbers per stage and TF category are summarized in fig. S13 (A to C). Age and gender information are shown in fig. S14 (A and B), respectively. Only plasma samples with an original sequencing depth ≥0.1× (using the earliest available time point if multiple samples existed for an individual) were included in downstream analyses. Raw sequencing depth information is shown in fig. S13D. Samples with accession numbers “EE87920,” “EE87921,” “EE86749,” and “EE86532” were excluded because quality control failure.

TFs were inferred using ichorCNA (*58*). As expected, healthy individuals generally exhibited TFs below 3% (figs. S1 and S13C), whereas plasma samples from patients with cancer displayed high variability in TF distribution (figs. S1 and S13, D to F). For each study, cancer type, and ichorCNA TF stratum, 70% of samples were assigned as the training set and 30% as the testing set. The detailed sample sizes after splitting are shown in table S8. Stage-stratified sample sizes for cross-validation, held-out test, and unseen test set are shown in fig. S1A.

### Annotation of genomic bins

The bin annotation was performed in four major steps: Step 1: Obtaining 100-kb bin annotations. The human genome (hg19) was divided into nonoverlapping, fixed-sized 100-kb bins. These annotations were retrieved directly from QDNAseq R package (*90*). Step 2: Adding additional bin information. The following annotations were added to each bin: open/close chromatin status based on GM12878 cell line from Xiong *et al.* (*91*), plus Chromosomal arm and chromosomal band information. Step 3: Filtering bins. The following bins were filtered out: bins overlapping with Duke Excluded Regions and ENCODE Data Analysis Center blacklisted regions (*92*, *93*); bins with residual values exceeding four times of their SD (an illustration of these blacklisted bins is shown as a red “Residuals” track in fig. S16F); bins with <95% characterized bases. Step 4: Merging bins: Fifty adjacent 100-kb bins were merged to form 5-Mb bins (*17*). A diagram of the bin-merging and re-indexing procedure is shown in fig. S16F. A detailed description of each step is provided in the Supplementary Materials.

### Annotation of cfDNA fragments

Quality control of the sequencing reads was conducted using the following criteria: retain only paired reads, remove duplicated reads, exclude secondary alignment and supplementary alignment, exclude unmapped reads, discard reads with discordant read names or missing strand information, discard outward-facing read pairs, reset fragment start to the start coordinate of the forward read, reset fragment end to the end coordinate of the reverse read, and remove out-of-bound reads (*87*). After quality control, read pairs were merged into cfDNA “fragments,” which were further filtered on the basis of the following criteria: retain fragments between 100 and 220 bp, retain fragments with map quality ≥30, and remove fragments containing insertions (I) and deletions (D) in their CIGAR string. The finalized fragment annotation file contains fragment-level metrics such as alignment coordinates, length, and motifs (table S6 and fig. S15A).

### Feature extraction and fragmentomic matrix preparation

Bin annotations were intersected with fragment annotations based on genomic coordinates (table S7). The resulting raw fragmentomic matrices were converted into two- or three-dimension matrices (collectively referred to as the “fragmentomic matrix” in this study), depending on the number of feature channels in each CNN model (Fig. 3F). For example, model C5 includes nine feature channels: “All,” “u2C,” “u2T,” “u1C,” “u1T,” “s1C,” “s1T,” “s2C,” and “s2T.” Definitions of these channels are shown in Fig. 3E. For example, u2C denotes fragments with a cytosine (C) at upstream position 2. s1C denotes fragments with the cytosine (C) at the 5′ start position. Values with each feature channel represent fragment counts matching a specific category. Feature channels were then stacked into a three-dimensional matrices and scaled jointly between 0 and 1. Multifeature data (vector features, matrices, and sample metadata) were integrated using the MultiAssayExperiment package (fig. S15B) (*94*). Each feature and fragmentomic matrix (after vector reshaping) is recorded as an “assay” in the MAE object (fig. S15, D and E). Feature channels from healthy individuals and cancer samples are shown as heatmaps in figs. S19 and S20.

Five marginal features were calculated from the fragmentomic matrix: (i) Len: fraction of fragments with lengths between 100 and 220 bp (1-bp increments); (ii) SD: SD of fragment counts across 5 Mb genomic bins for 100- to 220-bp fragments; (iii) CNA: fragment counts per bin were normalized for GC-content and mappability using LOESS and segmented using CBS (fig. S21) (*95*). Notably, Chr19 exhibited a systematic decrease in log_2_ ratios across its 5-Mb bins after GC correction and segmentation, likely due to its extreme gene density and high GC content (*96*, *97*). Prior studies raised concerns about including Chr19 in modeling (*58*, *98*). Given the potential biases and its limited data contribution (8 of 474 bins), Chr19 was excluded for both XGBoost and CNN models. (iv) S/L: defined as the ratio of 100 to 150 bp (short) to 151 to 220 bp (long) fragments. Counts were GC- and mappability-normalized before ratio calculation. (v) C/T: defined as the ratio of fragments with cytosine (C) at the 5′ end to those with thymine (T) at the 5′ end. As above, counts were normalized for GC-content and mappability before ratio calculation.

### Data splitting and attenuation of batch effects

cfDNA data generated across diverse laboratory protocols are susceptible to batch effects (*99*–*101*). Using fragment-length feature as an example, we examined sample-to-sample variability across the studies via principal component analysis (PCA). Len distributions are shown in fig. S15. PCA was performed using the prcomp function in the stats package (v4.3.2) (*102*) and visualized via the factoextra package (v1.0.7) (*103*). As expected, when samples were ordered by increasing tumor burden, fragment-length features showed progressive deviation from those of healthy individuals (fig. S18), consistent with fig. S24 (A, C, and D).

In our study, datasets were split into 70% training and 30% testing by sampling with each study, TF category and cancer type. This stratified sampling strategy attenuated domain differences between training and testing phases and avoided overcorrection of batch effects, which can reduce the generalization of models. Comparison of ichorCNA TF distributions between the two sets indicated no significant differences (fig. S2D). We further assessed whether the five marginal features used for modeling exhibited clustering or batch effects. PCA results show no major batch effects between training and testing datasets (fig. S2E). To quantify the impact of batch correction, UNITE-XGB models with and without batch effects removal. Removing batch effects reduced the models’ generalizability and degraded performance on completely unseen test samples (fig. S26, B to E). The allocation of samples by study, cancer type, and ichorCNA TF category to training and testing sets is summarized in tables S3 and S8.

### Logistic regression model training and testing

ichorCNA-inferred TFs were extracted from the MAE object and formatted as tabular data for the ichorCNA-TF model. Samples were labeled as Healthy = 0 and “Cancer” = 1. The ichorCNA-TF model was trained using the keras (v3.3.3) and the sklearn (v1.4.2) (*104*) libraries in Python. A nested cross-validation framework was used (fig. S26A). For outer cross-validation, StratifiedGroupKFold ensured that no patient appeared in both training and test folds. Ten repeats were conducted, each with five outer folds. For inner cross-validation, StratifiedGroupKFold was applied within each outer fold for hyperparameter tuning. A randomized grid search optimized hyperparameters. The best model from each outer fold was evaluated on the corresponding test fold using the following metrics: AUC, AUPRC, accuracy, sensitivity, specificity, F1 score, and PPV. These metrics were additionally computed at manually selected specificity thresholds (99 and 95%). Last, the full 70% training dataset was used to train the logistic regression model, and performance was assessed on the 30% held-out test set.

### UNITE-XGB and DELFI-XGB models training and testing

Both UNITE-XGB and DELFI-XGB use the XGBoost model architecture (*105*). For the UNITE-XGB model, the five features (i.e., Len, SD, CNA, S/L, and C/T) were extracted from the MAE object and converted into tabular format. Labels were assigned as Healthy = 0 and Cancer = 1. For the DELFI-XGB model, feature definitions followed the DELFI study (*17*), which includes the count of short and long fragments in 5-Mb bins, the copy number *z* score in bins, and the fraction of mitochondria fragments. In this study, we set the DELFI-XGB model’s training and testing procedure identical to that of UNITE-XGB.

Models were trained using keras (v3.3.3) (https://keras.io) and sklearn (v1.4.2) (*104*) in Python. A nested cross-validation framework was applied (fig. S26A). For outer cross-validation, StratifiedGroupKFold ensured that no patient appeared in both training and test fold. Ten repeats were conducted, each with five outer folds; for inner cross-validation, StratifiedGroupKFold was applied within each outer fold for hyperparameter tuning. A randomized grid search optimized hyperparameters. The best model from each outer fold was evaluated on the corresponding test fold using the following metrics: AUC, AUPRC, accuracy, sensitivity, specificity, F1 score, and PPV. These metrics were additionally computed at manually selected specificity thresholds (99 and 95%). For each model, SHAP (*59*, *106*) were used to interpret the contribution of each feature to the XGBoost predictions. As with the logistic regression workflow, the final models were trained on the full 70% training dataset and evaluated on the 30% held-out set.

### CNN model training and testing

We used three CNN architectures [ResNet18, ResNet34 (*47*), and EfficientNetV2S (*60*)] implemented in the timm (v1.0.7) module (https://pypi.org/project/timm/), each pretrained on ImageNet (https://image-net.org/), as the base model for fine-tuning. The input layer of these architectures expects three channels (i.e. RGB channels in an image; fig. S22). In our training pipeline, the number of channels in the first convolutional layer was modified to match the dimensionality of the fragmentomic matrix (Fig. 3F): (i) single-channel input (C1 model; Fig. 3F): The three pretrained channel weights were summed to create a single input channel. (ii) Multichannel input (C2 to C5 models; Fig. 5E): The initial three pretrained channel weights were repeated as necessary, and the first *n* channels were used (https://timm.fast.ai/models). The pretrained CNNs served as feature extractors, while the classification head was replaced with a binary output layer for Healthy and Cancer.

As with the XGBoost workflow, a nested cross-validation strategy (fig. S26A) was used to ensure robust model validation and hyper-parameter tuning, implemented through the skorch (v1.0.0) module (https://pypi.org/project/skorch/). Tuned hyperparameters included learning rate, number of epochs, and batch sizes. The entire 70% training set was used for model training, and the 30% held-out test set was used for performance evaluation. Model performance metrics were identical to those used for the XGBoost models. Further details on CNN customization are provided in the source code and the Supplementary Materials.

### Software, tools, and environments used in data analysis

All data preprocessing and visualization were performed in R, using packages including cfDNAPro, GenomeAlignments, AnnotationHub, QDNAseq, and Rsamtools. A complete list of R packages with version numbers is provided in table S9.

XGBoost models were trained using the scikit-learn (*104*) framework in Python (*107*). CNN models were trained using timm, pytorch, and skorch (https://pypi.org/project/skorch/) in Python. CNN model training was performed using NVIDIA L40S GPUs (https://nvidia.com/en-gb/data-center/l40s/). Singularity (*108*) (singularity-ce version 3.11.5-1.el8) was used to create the training container using the command: singularity pull docker://pytorch/pytorch. Software versions for all tools and Python modules are summarized in table S10. Steps for running the entire analysis are described in table S11.

### Statistical analysis

The Mann-Whitney *U* test was used to compare two groups, and the Kruskal-Wallis *H* test was used for comparisons involving more than two groups. The 95% confidence intervals of model performances across cross-validation repeats were computed using the smean.cl.boot function (nonparametric bootstrap, *n* = 1000) from the Hmisc R package (https://github.com/harrelfe/Hmisc). All statistical analyses were performed in R (*102*).

## Supplementary Materials

### The PDF file includes:

Supplementary Text Figs. S1 to S38 Tables S1 to S13 Legends for data files S1 and S2 References
