# Supplementary material for: A scalable deep-learning framework for cancer detection using cell-free DNA shallow whole-genome sequencing
Source: Sci Adv. 2026 Jul 10;12(28):eady9432. doi: 10.1126/sciadv.ady9432 (PMC13353424; doi:10.1126/sciadv.ady9432)
Supplement: Supplementary file 1 — Supplementary Text Figs. S1 to S38 Tables S1 to S13 Legends for data files S1 and S2 References [file sciadv.ady9432_sm.pdf]

Science Advances
AAAS

Supplementary Materials for

A scalable deep-learning framework for cancer detection using cell-free DNA shallow whole-genome sequencing

Haichao Wang *et al.*

Corresponding author: Hui Zhao, hui.zhao@cruk.cam.ac.uk; Nitzan Rosenfeld, n.rosenfeld@qmul.ac.uk

Sci. Adv. **12**, eady9432 (2026)
DOI: 10.1126/sciadv.ady9432

The PDF file includes:

Supplementary Text
Figs. S1 to S38
Tables S1 to S13
Legends for data files S1 and S2
References

Other Supplementary Material for this manuscript includes the following:

Data files S1 and S2

Supplementary Text

Materials and methods

NRLAB sample sequencing and analysis

Data trimming, alignment, and duplication marking were done using Trim Align Pipeline (TAP) (https://github.com/nrlab-CRUK/TAP)(*88*). The input FASTQ files were split into smaller chunks with 100,000 reads as default. Then, the file chunks were trimmed using library-specific trimming strategies. The extraction kit, library preparation kit, and sequencing platform information are available in **Table S2** and **S13**. Then, the trimmed FASTQ files were aligned using bwamem2 software(https://github.com/bwa-mem2/bwa-mem2). The chunks were merged and duplicates were marked using Picard tools(*110*). Base quality and realignment were performed for each file using GATK(*111*). Finally, all resulting bam files were downsampled to 1 million fragments using cfDNAPro (https://github.com/nrlab-CRUK/cfDNAPro). Two samples were excluded from downstream analyses due to quality control failure: "SLX-10572.D704-D505" and "SLX-11870.D712-D503". The version number of tools, software and R packages used in the analysis were summarized in **Table S9**.

EGA data processing

The datasets from Mouliere et al(*15*) (EGAS00001003258), Santonja et al(*58*) (EGAD00001008589 and EGAD00001006293), Ulz et al(*26*) (EGAD00001005343), Widman et al(*12*) (EGAD00001006237), Peneder et al(*27*) (EGAS00001005127) and Paracchini et al (EGAS00001004670)(*109*) were retrieved from EGA. Sequencing data was converted to FASTQ

format unless directly available from EGA and then processed using Trim and Align Pipeline (https://github.com/nrlab-CRUK/TAP).

FinaleDB data processing

The alignment coordinates of fragments in Tab-Separated Values (TSV) format of samples published by Jiang et al(*14*), Cristiano et al(*17*), Sun et al(*16*), Snyder et al(*24*) and Adalsteinsson et al(*59*) were downloaded from FinaleDB (finaledb.research.cchmc.org). According to the documentation of FinaleDB(*57*), the original reads were cropped to 50bp to reduce potential batch effects, then trimmomatic (v0.39)(*112*) was used to perform quality trim and Illumina adaptor clipping. The Alignment was done using bwa mem (v0.7.17)(*113*) to hg19. After alignment, Samblaster (v0.1.25)(*114*) was used to mark duplicates. The BAM files were then filtered using the following criteria: **(1)** Exclude any reads which are not properly paired; **(2)** Exclude any reads which are secondary alignments; **(3)** Exclude any reads which are supplementary alignments; **(4)** Exclude any reads that failed QC; **(5)** Exclude any reads that are PCR or optical duplicates.

From each filtered BAM file, the outer fragment endpoint coordinates for each pair of aligned reads were obtained. The distance between the endpoints for each fragment was defined as "fragment length". The fragment coordinates were stored in a TSV file accessible via FinaleDB. There are five columns in the TSV files recording "chromosome name", "start coordinate of the fragment", "end coordinate of the fragment", and "MAPQ score of the alignment (if the two paired reads have different MAPQ scores, the smaller one was chosen)" and "strand to which the first read is mapped". The "fragment profiles" (i.e. TSV files) were downloaded and converted to

GRanges object in R using plyranges R package(*22*). The resulting objects were saved in R Data Serialization (RDS) file format for further processing. (see the session of "Annotation of fragments")

Annotation of genomic bins

Step 1: Obtain 100kb bin annotation. The bin annotation (100kb bin size) was directly retrieved from QDNAseq R package(*90*). The human genome hg19 was divided into non-overlapping, fixed-sized bins of 100 kb. As described by Scheinin et al(*90*), the bin annotation contains five bin-wise statistics for quality control. The percentage of non-ambiguous nucleotides was calculated by dividing the number of nucleotides A, C, G, and T by the bin size (100 kb). This is used to adjust read counts for bins partially covered by uncharacterized nucleotides (N's) or in-complete bins at some regions of chromosomes in a reference genome build; The GC content of each bin was calculated as the number of C and G nucleotides divided by the number of total nucleotides (A, C, G, and T) in the reference genome within each bin. The term mappability denotes the average mappability of all 50-mers sequences within a bin, allowing for 2 mismatches and scaling the value from 0 to 100. These values were calculated from the ENCODE alignability track for 50-mers (https://www.genome.ucsc.edu/) with the bigWigAverageOverBed program (https://www.encodeproject.org/). Percent overlap with blacklist. The ENCODE blacklisted regions (March 2012 Freeze) were used to calculate the percentage of overlap with each bin. Residual values in each bin based on 1000 Genomes samples were generated: Publicly available samples from the 1000 Genomes Project that matched the experimental setup were downloaded (Illumina single-read of at least 50 bp, low depth, whole-genome sequencing). For samples with read lengths longer than 50 bp, the reads

were truncated to the first 50bp. In total, 38 samples that matched the experimental setup were available. Alignment and two-dimensional LOESS correction were then performed as outlined above. Residuals [(observed read count - LOESS fit)/LOESS fit] from the correction were recorded, and medians per bin were then calculated across the 38 samples. The cutoff for exclusion was set at 4 standard deviations. After bins exceeding this cutoff were removed, the LOESS correction was repeated without these anomalous bins and residuals. This process was repeated until the list of bins to be excluded stabilized.

Step 2: Add extra bin information**.** The "open/close chromatin" status, chromosome band and arm information were added to bin annotation. Open/Close chromatin status annotations were based on the GM12878 cell line from a study by Xiong et al(*91*). The authors developed SNIPER (Subcompartment iNference using Imputed Probabilistic ExpRessions), based on denoising autoencoder and Multilayer Perceptron (MLP) classifier to infer subcompartments using Hi-C datasets with moderate depth. The epigenomic features in the GM12878 cell line were calculated and released by the authors using their method with a resolution of 100 Kb. Chromosome band and arm information was retrieved from the AnnotationHub package(*115*).

Step 3: Filter bins**.** Bins overlapped with the Duke Excluded Regions (retrieved from AnnotationHub R package(*115*)). Duke Excluded Region indicates "regions for which mapped sequence tags were filtered out before signal generation and peak calling for Open Chromatin: DNaseI HS and FAIRE tracks. This track contains problematic regions for short sequence tag signal detection (such as satellites and rRNA genes)". An illustration of these blacklisted bins was displayed as a red "Blacklist Duke" track in **Fig. S16F**. Similarly, the figure only showed

the region between 84 Mb and 96 Mb surrounding the centromere of chromosome 3 for clarity purposes.

Bins overlapped with the DAC blacklisted regions (originated from ENCODE project) retrieved from AnnotationHub R package(*115*). According to the description: "The DAC Blacklisted Regions aim to identify a comprehensive set of regions in the human genome that have anomalous, unstructured, high signal/read counts in next-generation sequencing experiments independent of cell line and type of experiment. These regions tend to have a very high ratio of multi-mapping to unique mapping reads and high variance in mappability. Some of these regions overlap with pathological repeat elements such as satellite, centromeric, and telomeric repeats" (https://genome.ucsc.edu/). An illustration of these blacklisted bins was displayed as a "Blacklist DAC" track colored red in **Fig. S16F**. Of note, in the figure, only the region between 84 Mb and 96 Mb surrounding the centromere of chromosome 3 was picked as an example for demonstration purposes.

Bins with residual values falling outside of 4 times its Mean Absolute Deviation (MAD) were discarded. An illustration of these blacklisted bins is displayed as a red "Residuals" track in Fig. **Fig. S16F**. Similarly, the figure only showed the region between 84 Mb and 96 Mb surrounding the centromere of chromosome 3 for clarity purposes. Bins with less than 95% characterized bases were discarded. These blacklisted bins were displayed as "Bases < 95%" track in **Fig. S16F**. Similarly, the figure only showed the region between 84 Mb and 96 Mb surrounding the centromere of chromosome 3 for clarity purposes.

A comparison between the distribution of raw (Grey-colored) and filtered (Green-colored) bin statistics is shown in **Fig. S16**. As expected, after filtering, bins overlapping with the blacklist region were discarded (**Fig. S16A**); Those bins with ≥ 5% uncharacterized bases were removed (**Fig. S16B**). After blacklisting, while there are only subtle changes in GC content (**Fig. S16C**), bins with extremely low mappability were removed (**Fig. S16D**). The residual filtering didn't change the distribution except for the left tail region (**Fig. S16E**).

Step 4: Merge adjacent bins to get larger bins for downstream analysis**.** The atomic bins (100 Kb) were merged to achieve a larger bin size for downstream analysis(*17*). **Fig. S16F** illustrates the bin merging and re-indexing process. Using 1 Mb target bin size as an example, the merging was performed as follows: Counting from the p-arm telomere to the centromere, the first ten 100 kb bins were labelled with integer 1, indicating all these 10 atomic bins (100kb) belong to the first 1 Mb bin. Then, the next 10 atomic bins (100kb) were labelled with integer 2, etc. When approaching the centromere, discard the remaining bins that are too few to be grouped into 1 Mb bin (i.e. less than 10 atomic bins left). Counting from the q-arm telomere to the centromere, repeat what has been done in step 1. Sort the order of the 1 Mb bins to follow this order: from chromosome 1 to chromosome 22, from the far end of the p-arm telomere to the centromere, and then from the centromere to the far end of the q-arm telomere. After sorting, re-index the bins with this new order.

In summary, the finalized 100kb bin annotation files contain various information: (1) Bin index. (2) Bin coordinates (chromosome, start, end of the bin); (3) Bin QC statistics (the percentage of non-N bases, GC content, mappability, the percentage overlap with ENCODE blacklisted

regions, residual values); (4) Additional bin annotations (i.e., arm, open/close chromatin status). A detailed explanation of each column is shown in **Table S4**. In addition, an example of a 1 MB bin annotation (for simplicity reasons, not all rows are shown) is provided in **Table S5**.

Annotation of cfDNA fragments

Step 1: The reads were filtered with following criteria: (1) Only keep paired reads; (2) Not duplicated reads; (3) Not secondary alignment; (4) Not supplementary alignment; (5) Not unmapped reads; (6) Discard reads with read name discordance; (7) Discard reads without strand name; (8) Discard outward-facing read pairs; (9) Reset fragment start coordinate as the start of the forward read; (10) Reset fragment end coordinate as the end of the reverse read. (11) Remove out-of-bound reads.

Step 2: the read-pairs were merged into cfDNA "fragments", which were filtered based on the following criteria: (1) Only keep fragments between 100-220bp fragments; (2) Only keep fragments with map quality ≥ 30; (3) Remove fragment containing insertion (i.e. I) and deletion (i.e. D) in its CIGAR string. Multiple fragment-wise metrics, such as alignment coordinates, length, and motifs, were calculated (**Table S6**). The bin and fragment annotation were intersected based on the coordinates (**Fig. S15A**).

Deep Learning model training and testing

We utilized three CNN architectures (i.e., ResNet18(*47*), ResNet34(*47*), and EfficientNetV2S(*61*)) implemented in the timm (v1.0.7) module (https://pypi.org/project/timm/), pre-trained on the ImageNet(https://www.image-net.org/), as the base model for fine-tuning. ResNet18 and ResNet34 These are part of the ResNet (Residual Network) family(*47*), designed to combat the vanishing gradient problem by using skip connections that allow gradients to flow more easily during backpropagation. ResNet18 has 18 layers, and ResNet34 has 34. The main difference between the two is that ResNet34 has more layers, allowing it to capture more complex features at the cost of increased computational load; EfficientNetV2 is a newer architecture designed with a compound scaling method, which scales depth, width, and resolution simultaneously, aiming for a more optimized balance between accuracy and computational efficiency. The "S" (small) variant is lighter in computational load than larger variants(*61*).

The input image of those base models has three channels (i.e. RGB channels in an image). In our training, we adjusted the number of channels of the first convolutional layer to match dimensions of the input (**Fig. 3F**): (1) When the input fragmentomic array has one channel (i.e., C1 model in **Fig. 3F**), the 3 channel weights were summed into a single channel. (2) When the input has n channels (n > 1) (i.e., C2-C5 models in **Fig. 3F**), the initial three channel weights were repeated as many times as required (when necessary), and the first n channels were used (https://timm.fast.ai/models). The pre-trained models were used as feature extractors; the top layers were reset to two classes (i.e., “Healthy” and “Cancer”) in our binary classifiers training.

A nested cross-validation (**Fig. S26A**) approach was employed to ensure robust model evaluation and hyper-parameter tuning through a framework provided by the skorch (v1.0.0) python module (https://pypi.org/project/skorch/): For the outer cross-validation , 5-fold cross-validation with 10 repeats was performed. For the inner cross-validation, a randomized grid search using 3-fold cross-validation was performed to optimize the hyper-parameters. CNN model evaluation was performed the same as the XGBoost model. All data pre-processing (e.g., data trimming, alignment, bin and fragment annotation, tensor preparation and feature extraction) and visualization steps (e.g., feature visualization, tensor visualization, model performance plotting) were performed using R(*102*). Details on the software used are shown in **Table S9**.

XGBoost model was trained using the scikit-learn(*104*) framework in Python(*107*). CNN model was trained using pytorch and skorch (https://pypi.org/project/skorch/) framework in Python. All training was performed using NVIDIA® L40S Graphics Processing Unit (GPU) cards (https://www.nvidia.com/en-gb/data-center/l40s/). Models were trained using a reproducible environment, i.e., a Singularity(*108*) (singularity-ce version 3.11.5-1.el8) image pulled from the Docker hub using the command: ***singularity pull docker://pytorch/pytorch***. This pulls the official PyTorch repository on Docker Hub, which contains NVIDIA® GPU-supporting containers using CUDA® for processing. The detailed version numbers of the software and Python modules used are shown in **Table S10**.

Supplementary Results

Comparison of UNITE-XGB and UNITE-CNN across TF categories

In samples with TF lower than 3%, UNITE-CNN achieved higher AUC than UNITE-XGB (0.904 vs 0.878, $p \leq 0.0001$) (**Fig. S8A**). In addition, UNITE-CNN achieved mean AUPRC of 0.939 (95%CI: 0.933-0.94), sensitivity at 0.99 specificity of 0.329 (0.286-0.37), and sensitivity at 0.95 specificity of 0.545 (0.507-0.58). In contrast, UNITE-XGB had worse performance of 0.922 (0.917-0.93), 0.253 (0.22-0.29), and 0.467 (0.435-0.5) respectively (**Fig. S8A**).

UNITE-CNN achieved AUC of 0.937, 45% sensitivity at 99% specificity, 74.5% sensitivity at 95% specificity, while UNITE-XGB achieved 0.911, 16.5% and 70.5% respectively (**Fig. S10A**). The overlaps regarding samples detected by UNITE-CNN and UNITE-XGB models are shown as a Venn diagram (**Fig. S10B**). For example, at 99% specificity, UNITE-XGB and UNITE-CNN detected 34 and 90 true cancer samples, respectively (28 were detected by both models). The prediction scores of samples from the healthy and 15 cancer types are shown in **Fig. S10C-D**. We observed that UNITE-CNN had better sensitivity (at 99% specificity) than UNITE-XGB in 12/15 cancer types. For example, in lung and breast cancer, the UNITE-CNN model achieved sensitivity of 29.41% and 67.65% at 99% and 95% specificity respectively, which are higher than XGBoost (5.88% and 61.76%).

The result from (0.03, 0.1] TF category is shown in **Fig. S11**. UNITE-XGB had higher ($p \leq 0.001$) AUC (mean: 0.962, 95% CI: 0.957-0.97) and AUPRC (0.952, 0.947-0.96) than UNITE-CNN (AUC = 0.943, 0.935-0.95; AUPRC = 0.931, 0.923-0.94) (**Fig. S11A**). While the sensitivity at 0.99 specificity is not significantly different (0.546, 0.475-0.62 vs 0.521, 0.453-0.59), UNTIE-XGB had higher sensitivity at 0.95 specificity than UNITE-CNN (0.78, 0.738-0.82 vs 0.734, 0.689-0.77) ($p \leq 0.01$). In the held-out testing, UNITE-XGB had higher AUC than UNTIE-CNN

(0.993 vs 0.962), which is also reflected by the Venn diagram and prediction scores (**Fig. S11B-D**). In the (0.1, 1] TF category, all models had near-perfect performance (**Fig. S12**).

Supplementary Figures

A

	Healthy	I	II	III	IV	NA	*Row sum*
**Cross-validation**	325	57	85	40	310	420	1237
**Held-out test**	133	13	35	11	114	147	453
**Unseen test**	173	49	32	50	58	11	373
Column sum	*631*	*119*	*152*	*101*	*482*	*578*	*2063*

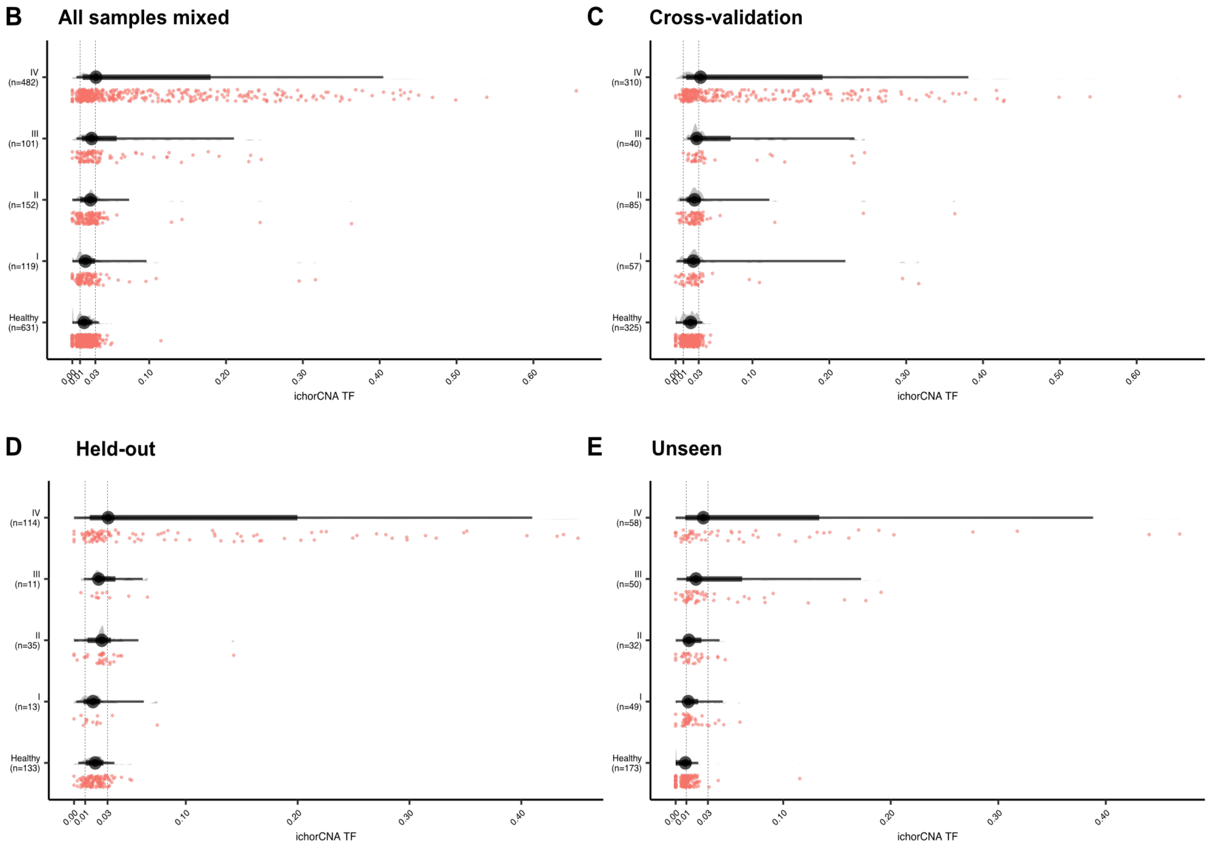

Fig. S1. Sample size and tumor fraction distribution across stages and data divisions. (**A**) The number of samples across stages allocated to cross-validation, held-out test and unseen test. (**B-E**) The ichorCNA TF distribution in different cancer stages across all, cross-validation, held-out test, and unseen test, respectively.

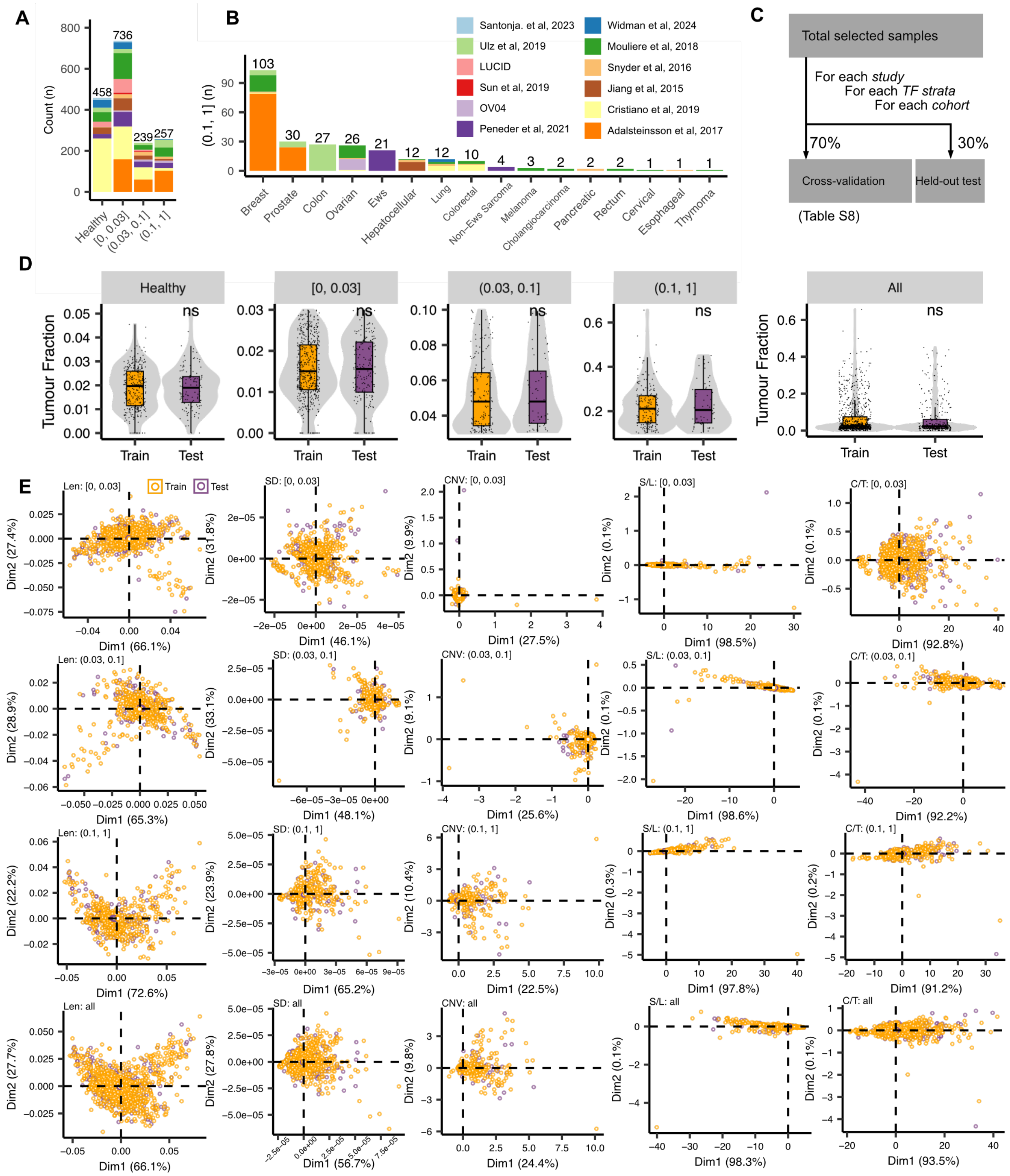

Fig. S2. Sample stratification and data splitting for cross-validation and held-out test. (A-B) Sample stratification by TF. **(C)** Stratified data division into training and testing groups. **(D)** Comparison of tumor fraction (TF) distributions between training and testing datasets across ichorCNA TF categories. **(E)** Principal component analysis (PCA) of batch effects between training and testing data across all features and TF categories. Rows show TF categories; columns show marginal features. Group means are represented by larger symbols, with 95% confidence ellipses around each cluster. No significant domain-wise differences were observed between training and testing datasets. For visualization clarity, one outlier sample (sample ID: EE87123) with extreme values in the C/T

feature was excluded from the PCA plots; inclusion did not affect conclusions. Statistical significance: ns (not significant); *p ≤ 0.05; **p ≤ 0.01; ***p ≤ 0.001; ****p ≤ 0.0001.

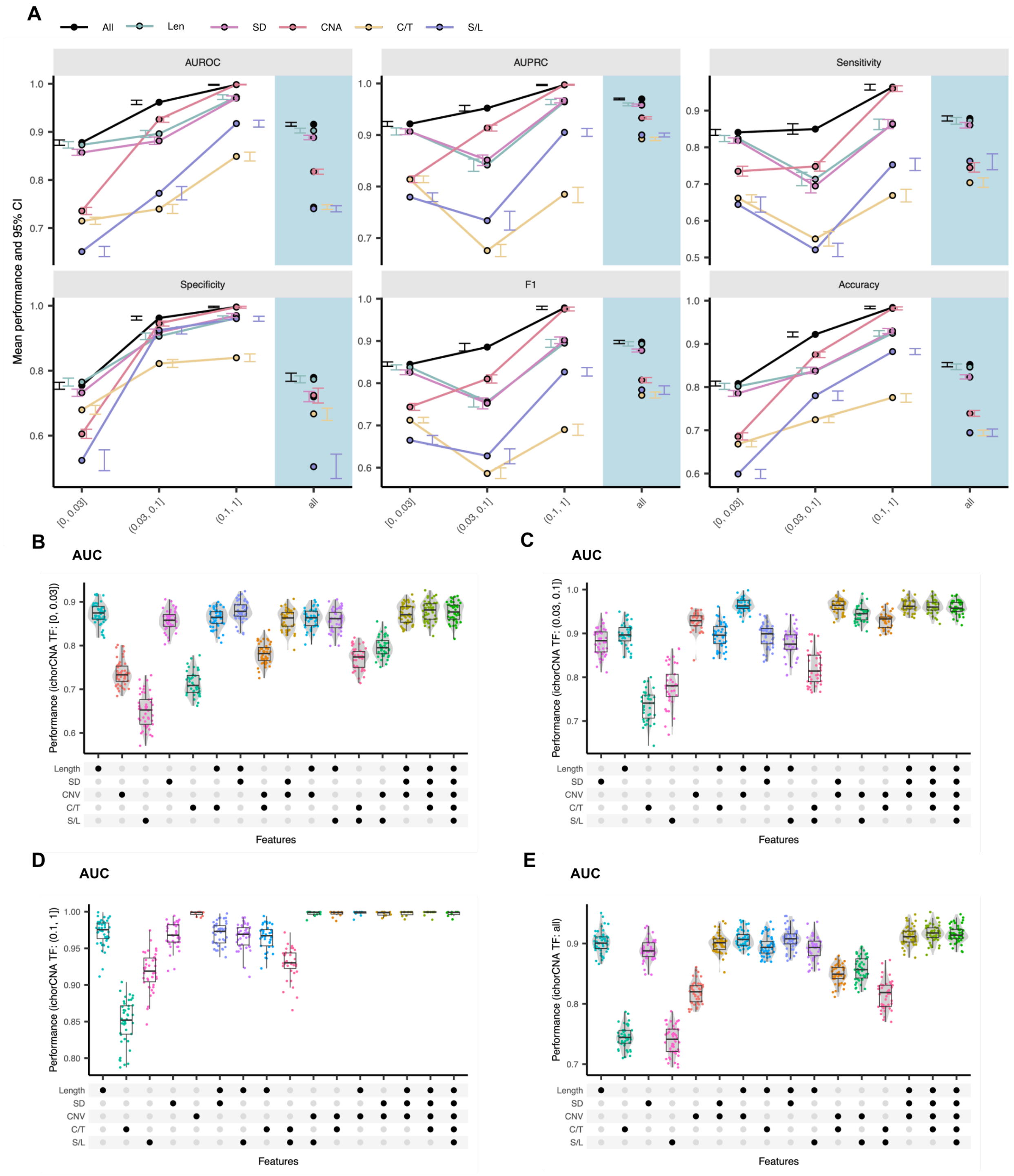

Fig. S3. Extended feature combinations of XGBoost models and performances. (A) AUC, AUPRC, Sensitivity, Specificity, F1 and Accuracy of XGBoost models with independent feature or ensemble feature spaces. **(B-E)** The AUC of XGBoost models with various combinations of features as input for the ichorCNA TF strata of "[0, 0.03]", "(0.03, 0.1]", "(0.1, 1]" and "all" (i.e., "[0, 1]"), respectively.

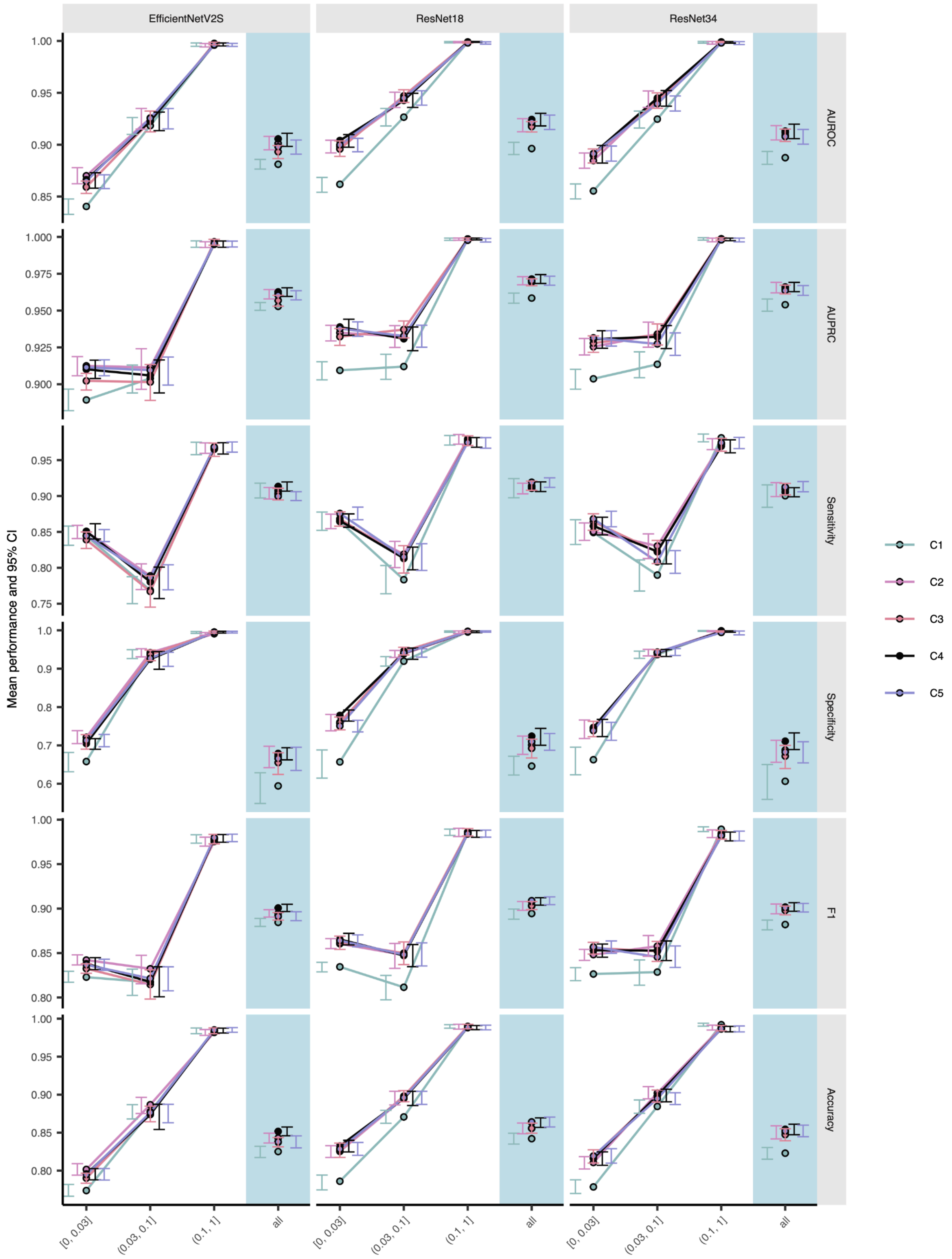

Fig. S4. Performances of various CNN architectures. Various metrics obtained from EfficientNetV2S, ResNet18 and ResNet34 were shown in the figure.

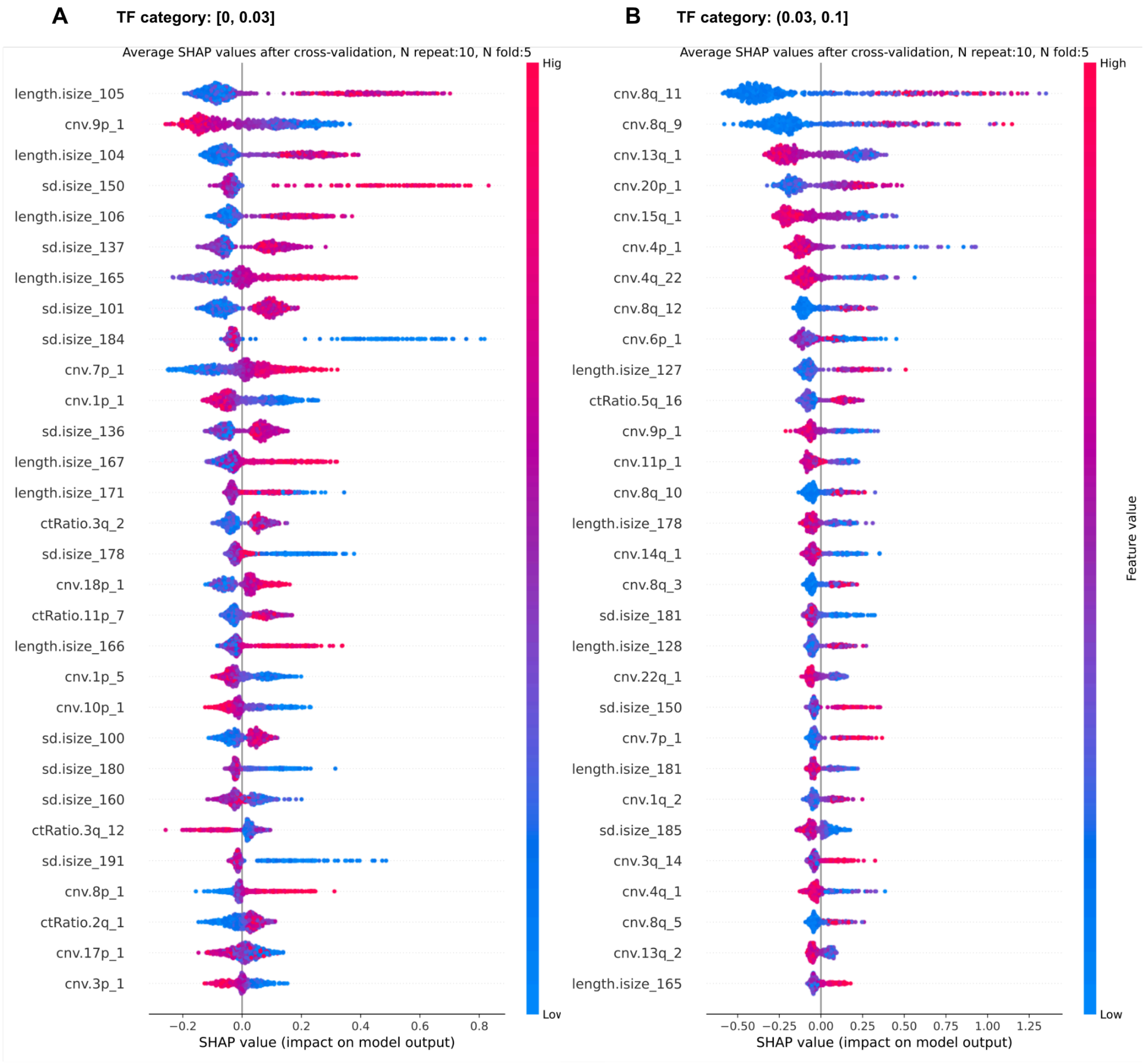

Fig. S5. Average SHAP values of features in XGBoost X6 models (i.e., UNITE-XGB) for TF [0, 0.03] and (0.03, 0.1] categories. Values are averaged through 5-fold cross-validation, 10 repeats. (**A**) Results for the ichorCNA TF strata of "[0, 0.03]". (**B**) Results for ichorCNA TF strata of "(0.03, 0.1]". The variable names contain feature types, e.g., length.isize_105 denotes 105bp fragment length; ctRatio.4q_22 means the C/T ratio of the 22nd bin on Chr 4.

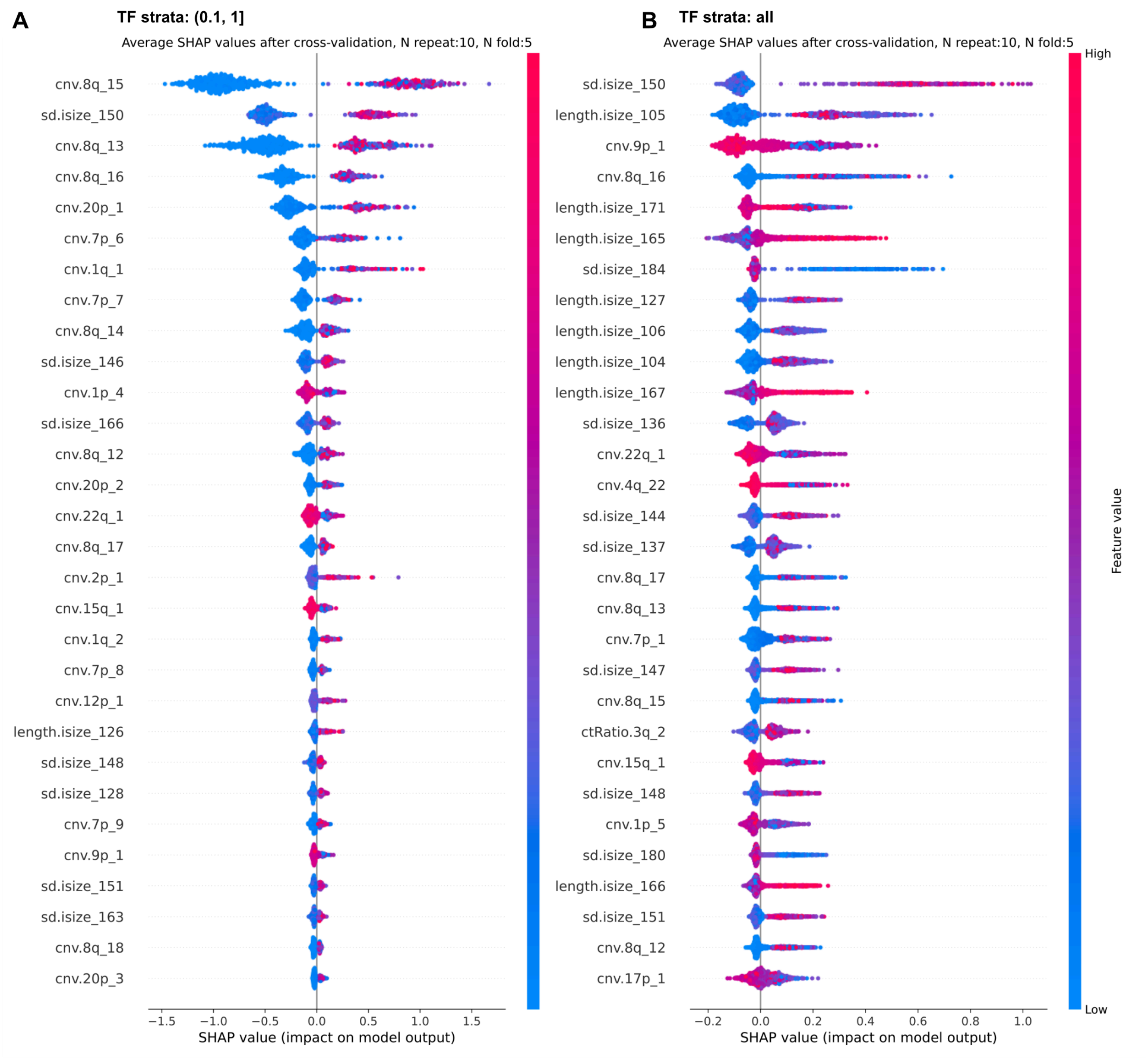

Fig. S6. Average SHAP values of features in XGBoost X6 model for TF (0.1, 1] and all categories. Values are averaged through 5-fold cross-validation, 10 repeats. (**A**) Results for the TF stratum of "(0.1, 1]". (**B**) Results for the TF stratum of "all".

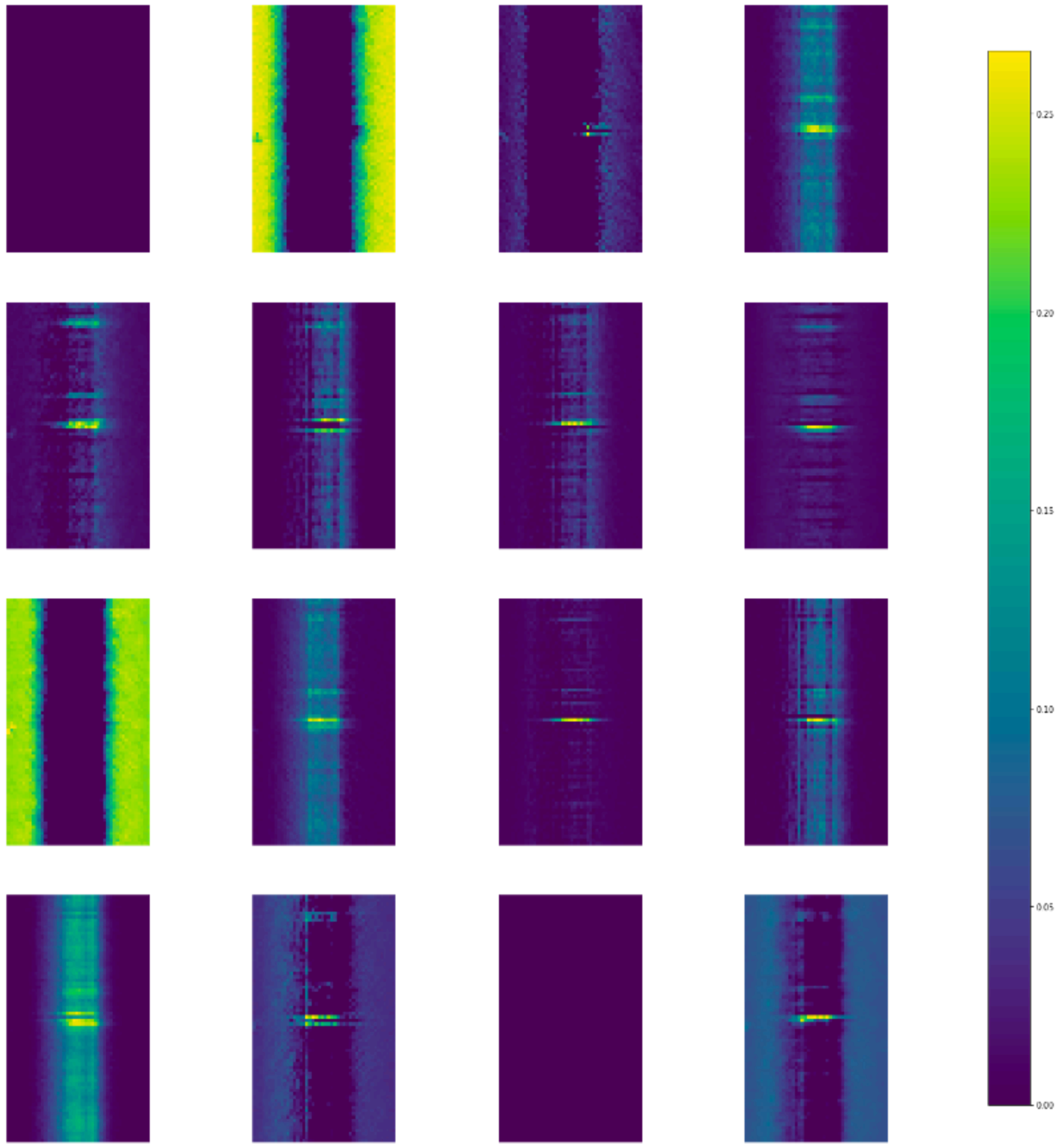

Fig. S7. An example of activation maps of a cancer sample. Heatmaps denote the results from various filters in the final convolutional layers. Brighter colors indicate the attention of the model, highlighting bins with aberrant copy number and fragment lengths. The heatmap here is only for demonstration purposes.

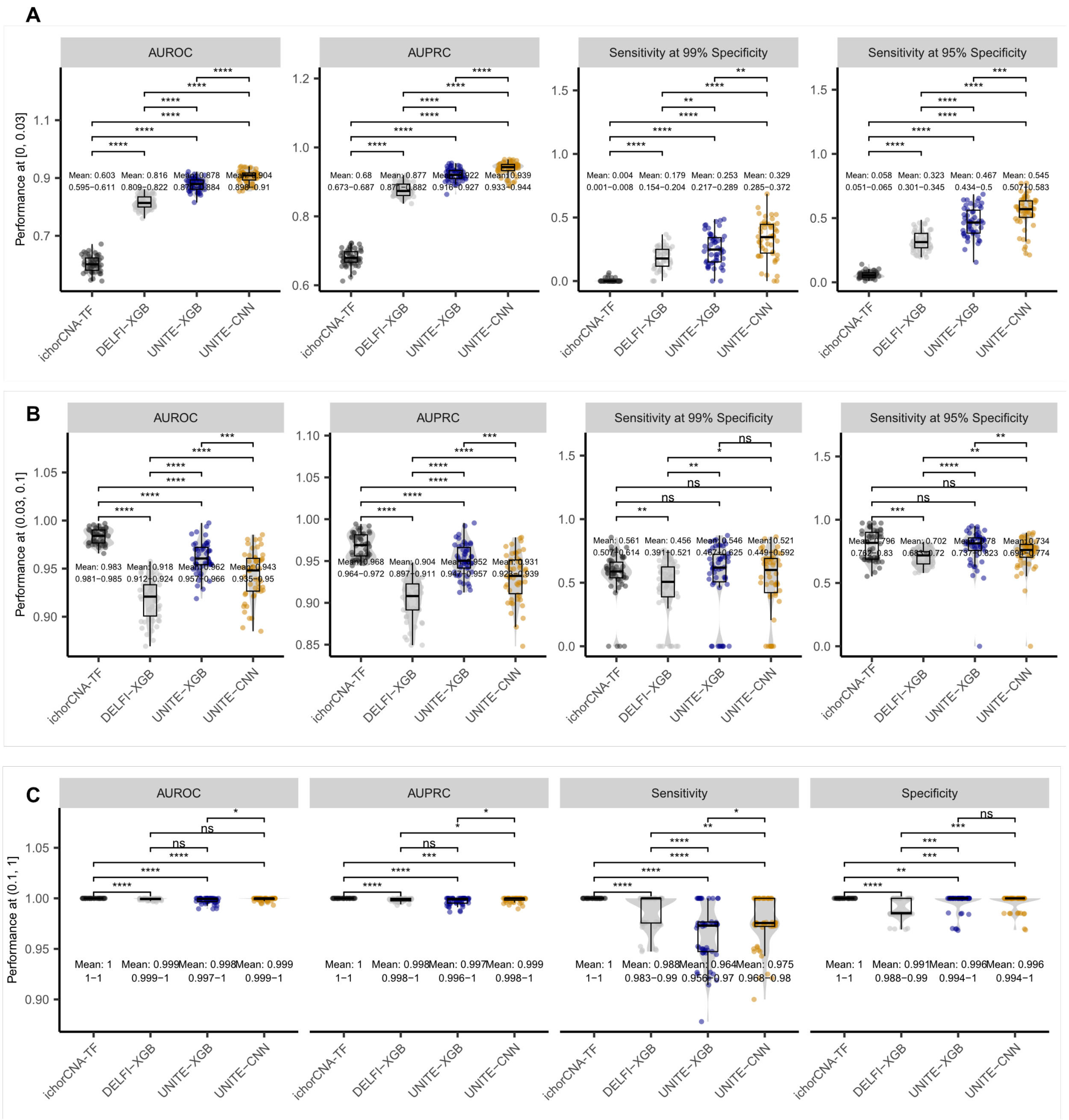

Fig. S8. Metrics comparison between models trained and tested in various TF categories. Results are those from cross-validation. (**A**), (**B**) and (**C**) shows the metrics of [0, 0.03], (0.03, 0.1] and (0.1, 1], respectively. Source data are provided as Data file S2. ns: Nonsignificant; *: p ≤ 0.05; **: p ≤ 0.01; ***: p ≤ 0.001; ****: p ≤ 0.0001.

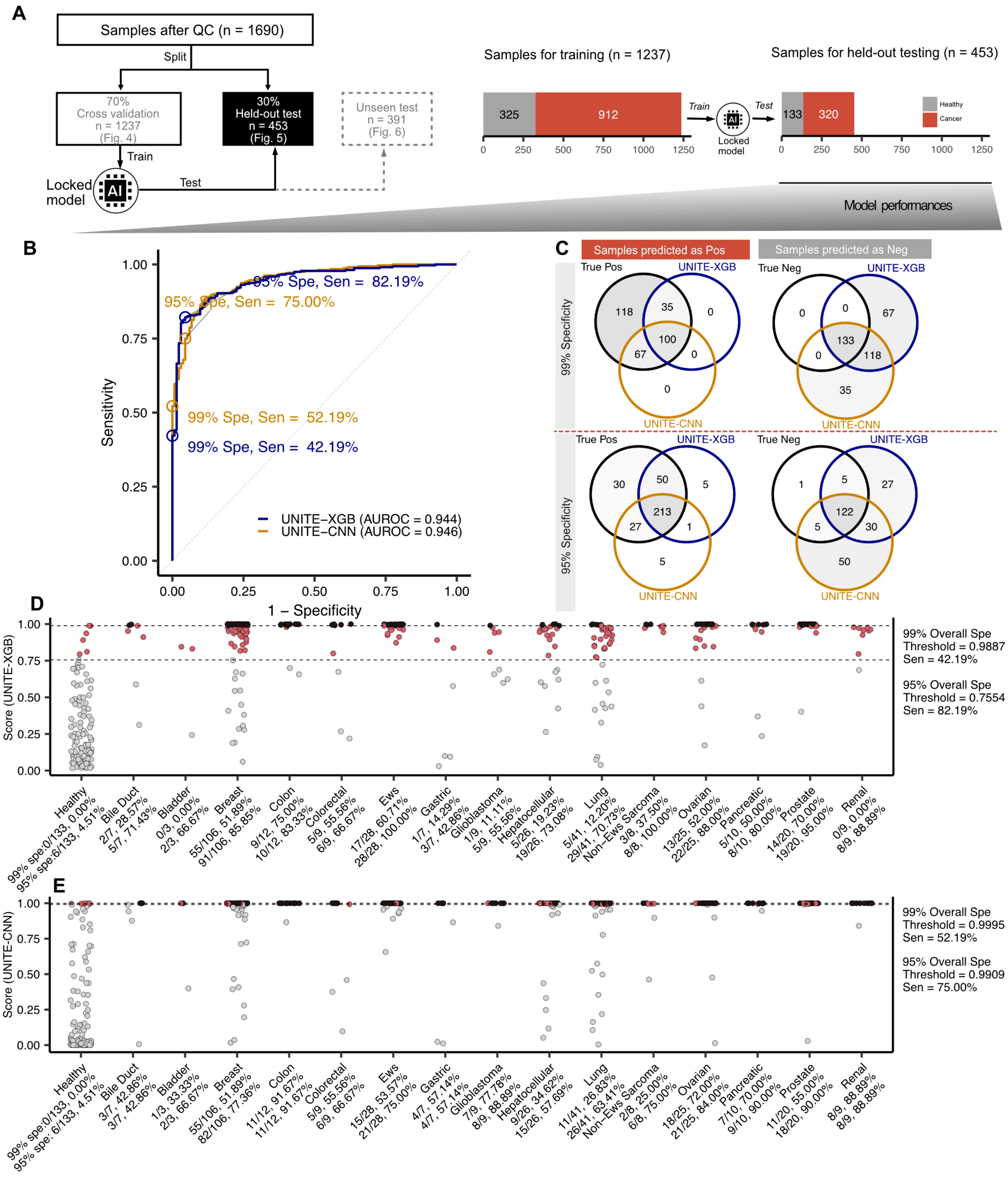

Fig. S9. The comparison between the performance of UNITE-XGB and UNITE-CNN model in held-out samples. (A) Models were trained using the entire cross-validation set and tested on the entire held-out test set. (**B**) ROC of UNITE-XGB and UNITE-CNN model in the 30% held-out test set. (**C**) Venn diagram showing the overlaps between samples predicted by UNITE-XGB and UNITE-CNN models. (**D**) Scores of each sample in the testing dataset predicted by the UNITE-XGB model. (**E**) Scores of each sample in the testing dataset predicted by the ResNet18 model. Mann-Whitney U test was conducted. Source data are provided as Data file S2.

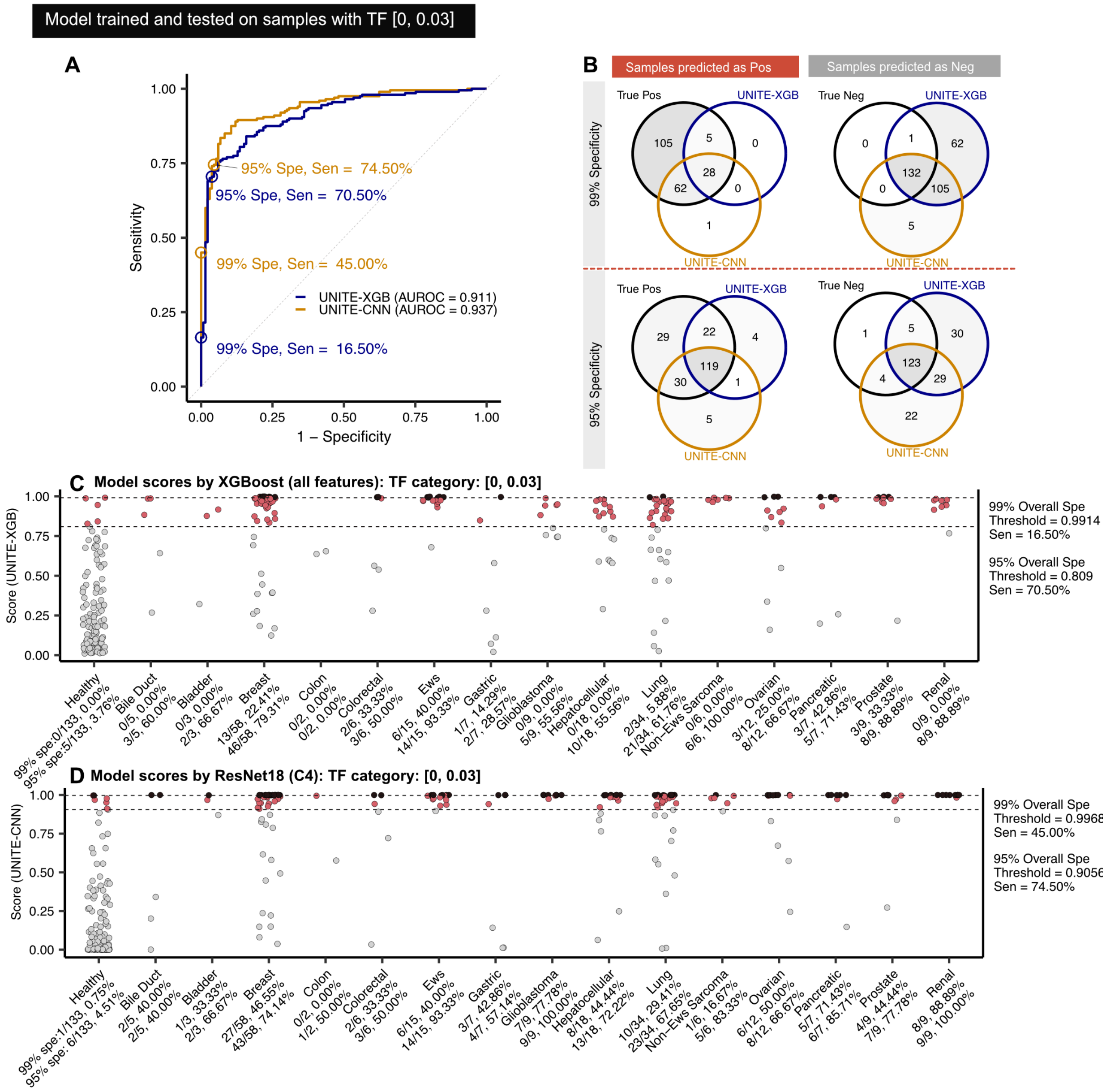

Fig. S10. The comparison between UNITE-XGB and UNITE-CNN in samples with [0, 0.03] TF in held-out samples. (**A**) ROC of UNITE-XGB and UNITE-CNN model in the 30% testing dataset. (**B**) Venn diagram showing the overlaps between samples predicted by UNITE-XGB and UNITE-CNN models. (**C**) Scores of each sample in the testing dataset predicted by the UNITE-XGB model. (**D**) Scores of each sample in the testing dataset predicted by the UNITE-CNN model. Mann-Whitney U test was conducted. Source data are provided as Data file S2.

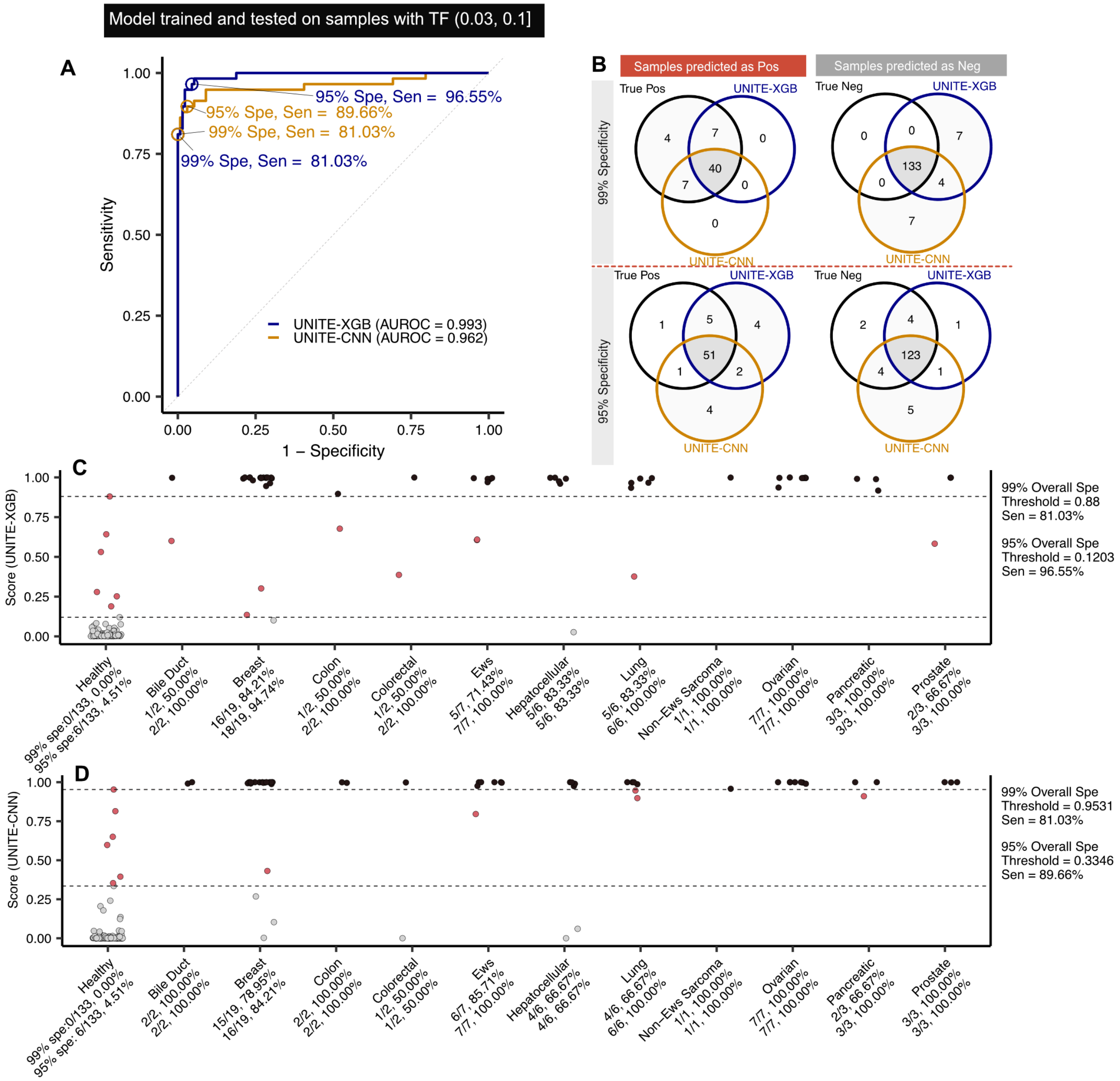

Fig. S11. The comparison between UNITE-XGB and UNITE-CNN model in samples with (0.03, 0.1] TF in held-out samples. (**A**) The performance in the 5-fold cross-validation (10 repeats) using 70% training data. (**B**) ROC of UNITE-XGB and UNITE-CNN model in the 30% testing dataset. (**C**) Venn diagram showing the overlaps between samples predicted by UNITE-XGB and UNITE-CNN models. (**D**-**E**) Scores of each sample in the testing dataset predicted by the UNITE-XGB and UNITE-CNN model. Mann-Whitney U test was conducted. Source data are provided as Data file S2.

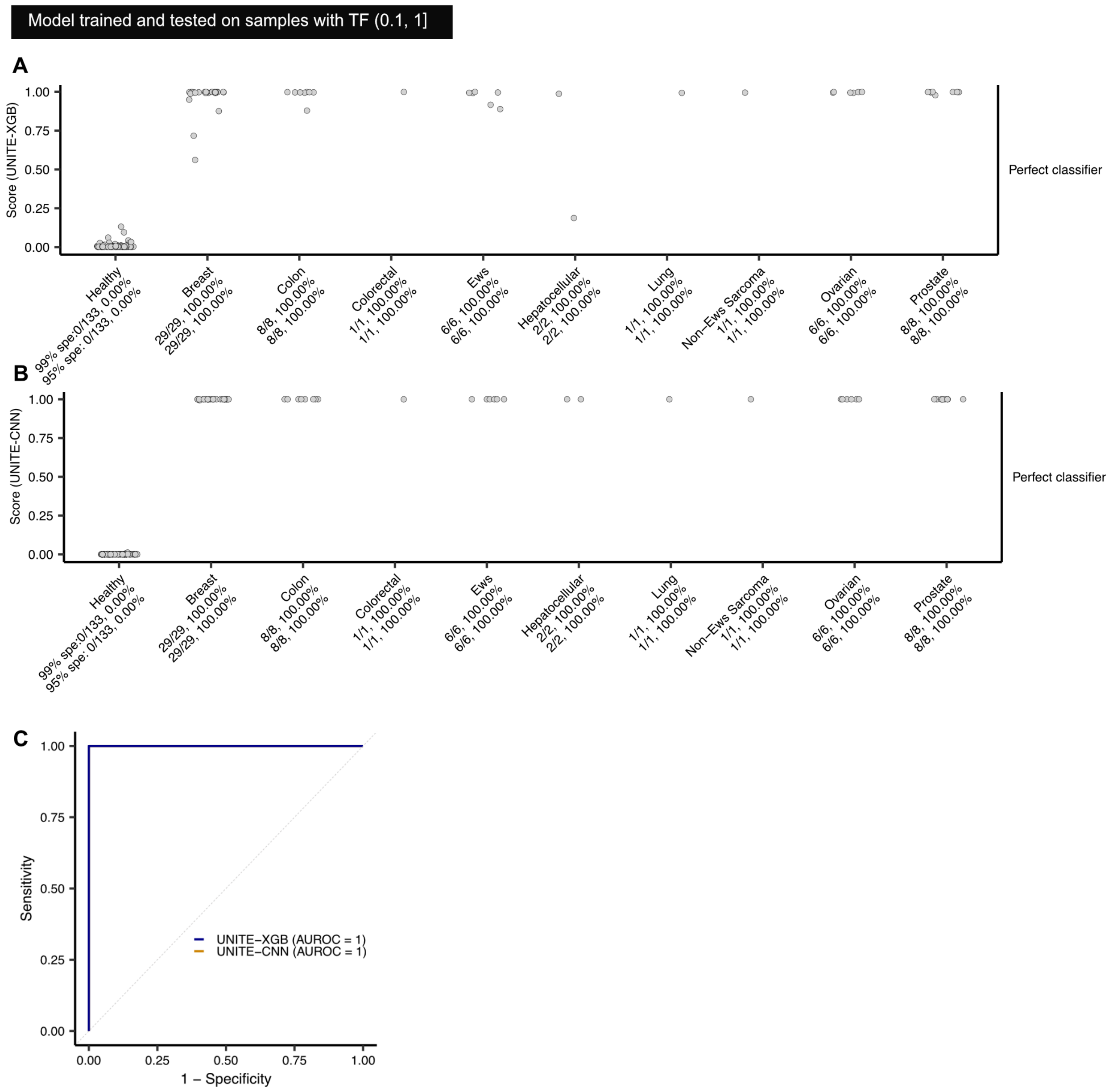

Fig. S12. The comparison between UNITE-XGB and UNITE-CNN model in samples with (0.1, 1] TF in held-out test set. (**A-B**) As expected, the UNITE-XGB and UNITE-CNN achieved 100% accuracy. (**C**) The AUC of models.

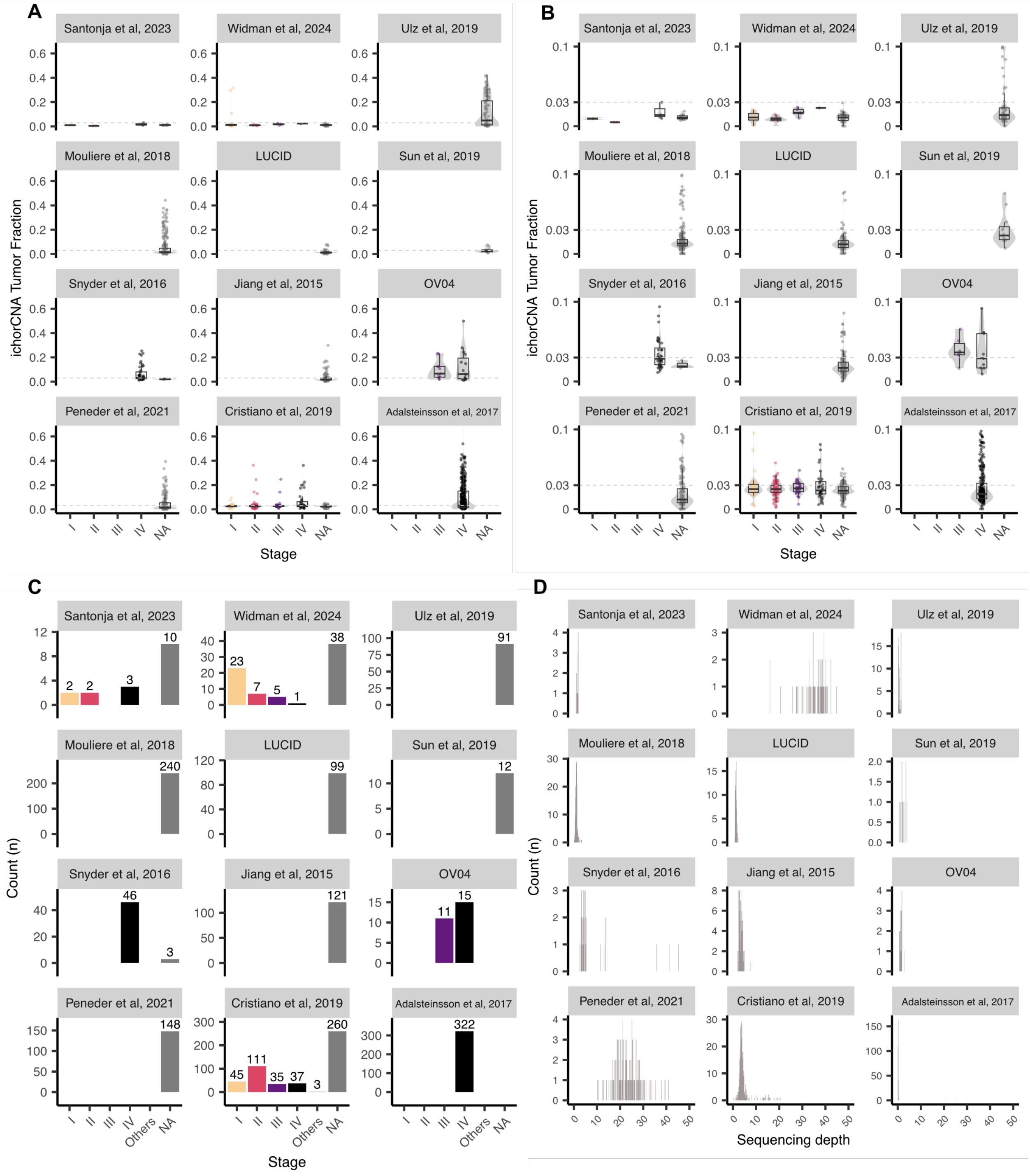

Fig. S13. Cancer stage, TF and sequencing depth of those selected samples for cross-validation and held-out test. (**A**) The ichorCNA TF of samples from various stages stratified by author. (**B**) The same data as panel A but zoom-in y-axis between 0 and 0.1. (**C**) The number of samples attributed to various stages across studies. (**D**) The raw data sequencing depth.

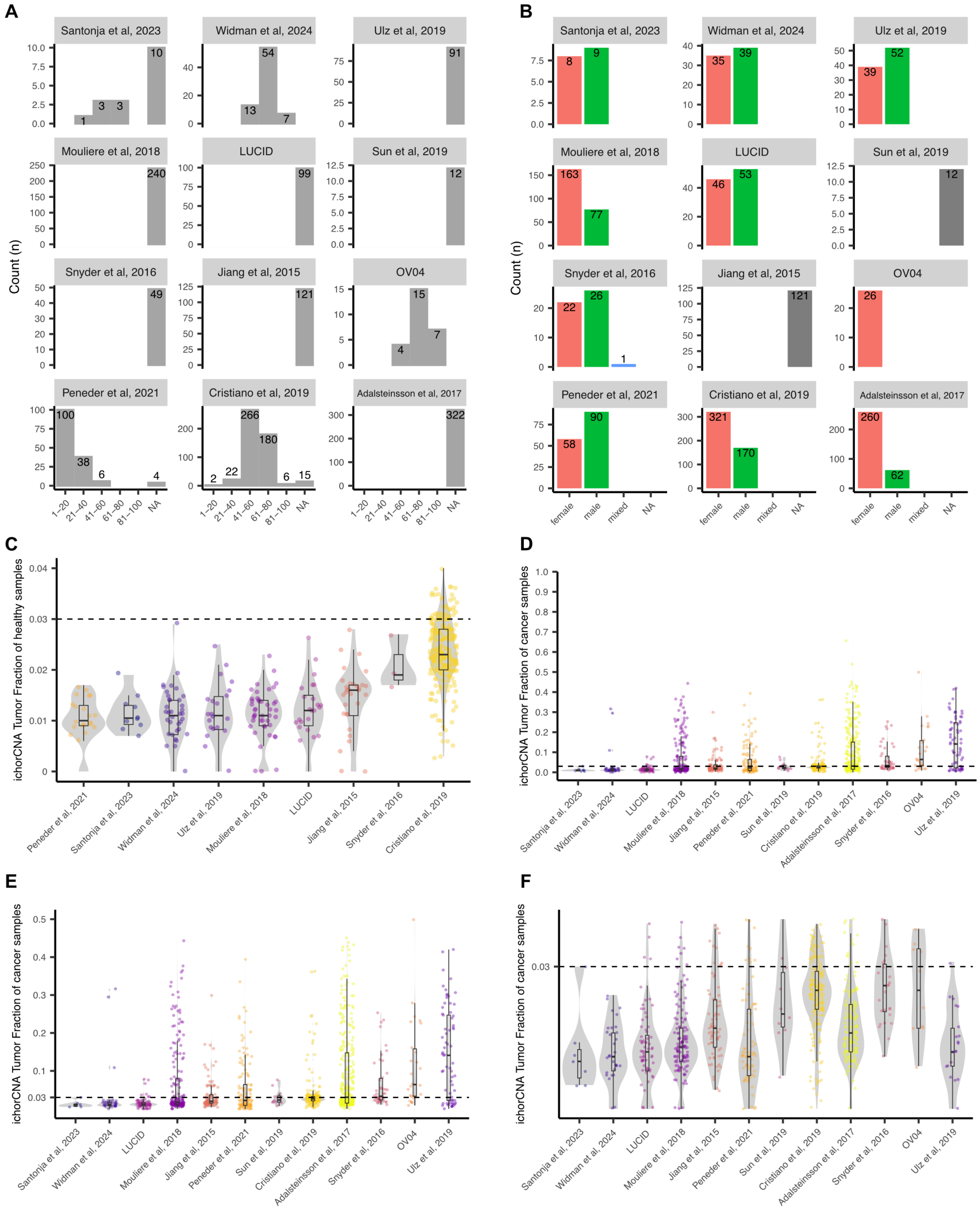

Fig. S14. Age, gender and TF of those selected samples for cross-validation and held-out test. (**A**) Age information of samples. (**B**) Sex information of samples. (**C**) ichorCNA TF of all healthy samples grouped by author. The x-axis is sorted by the median TF of datasets. (**D**) ichorCNA TF of all cancer samples faceted by author. (**E**) The same data as (**D**) but only showing samples with TF between 0 and 0.5. (**F**) The same data as (**D**) but only showing the samples with TF between 0 and 0.04.

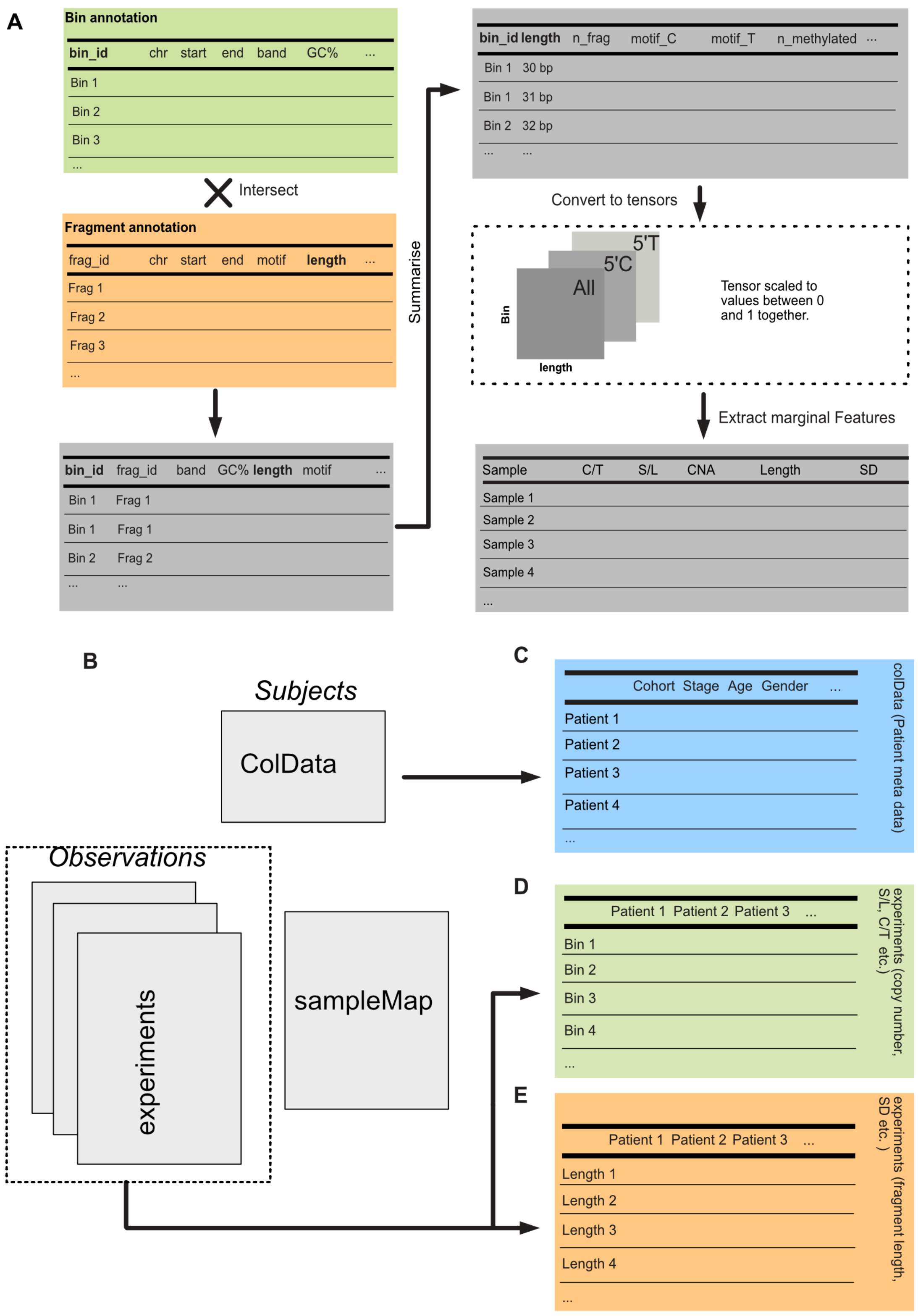

Fig. S15. Bin-fragment annotation intersection and feature extraction. (**A**) A schema showing the bin-fragment annotations and the intersection as well as downstream feature extraction and reshaping. (**B**) A schema showing the feature integration eco-system implemented in the MultiAssayExperiment R package for best practices in

reproducible science. (**C**) The tabular data prepared for patient meta-information. (**D**) and (**E**) Examples of individual assays (i.e., cfDNA "features" in the context of this study).

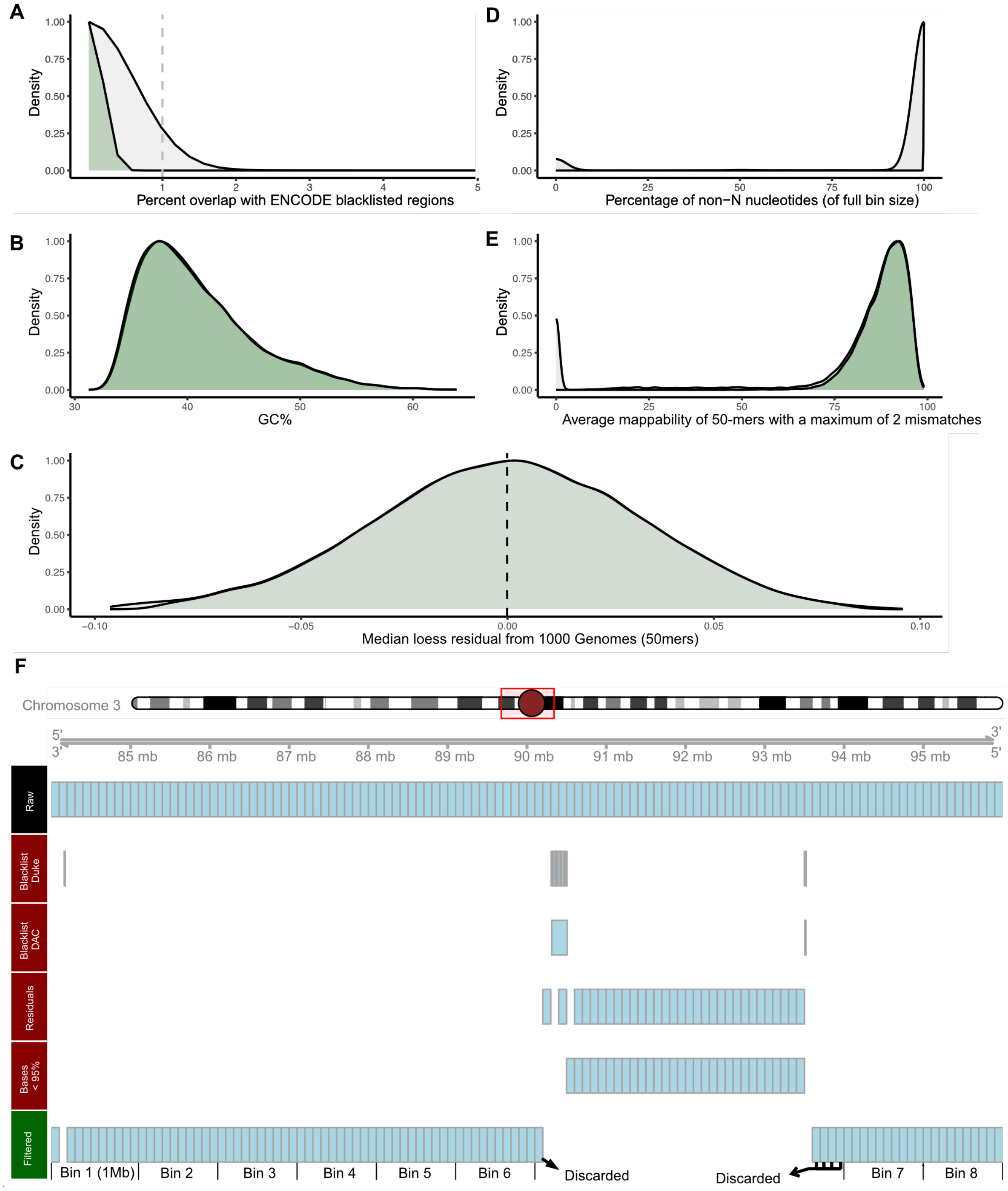

Fig. S16. Bin quality control and merging. (**A-E**) A comparison between raw and filtered bins. Grey and green areas indicate the bin statistics before and after filtering: (**A**) Percent overlap with ENCODE blacklisted regions. The

vertical dashed line indicates a 1% overlap. (**B**) Percentage of non-N nucleotides. (**C**) GC content. (**D**) Average mappability of 50-mers with a maximum of 2 mismatches. (**E**) The distribution of residuals. (**F**) Bin filtering and merging. An area surrounding the centromere was highlighted using a red rectangle located between 84 Mb and 96 Mb on chromosome 3. The black track indicated the raw (i.e., unfiltered bins); Red tracks illustrated the bins discarded, which are Duke and DAC blacklist regions, bins with extreme residual values, and bins with less than 95% characterized nucleotides (i.e., A/C/T/G). The green track showed the finalized filtered bins used in the downstream analysis.

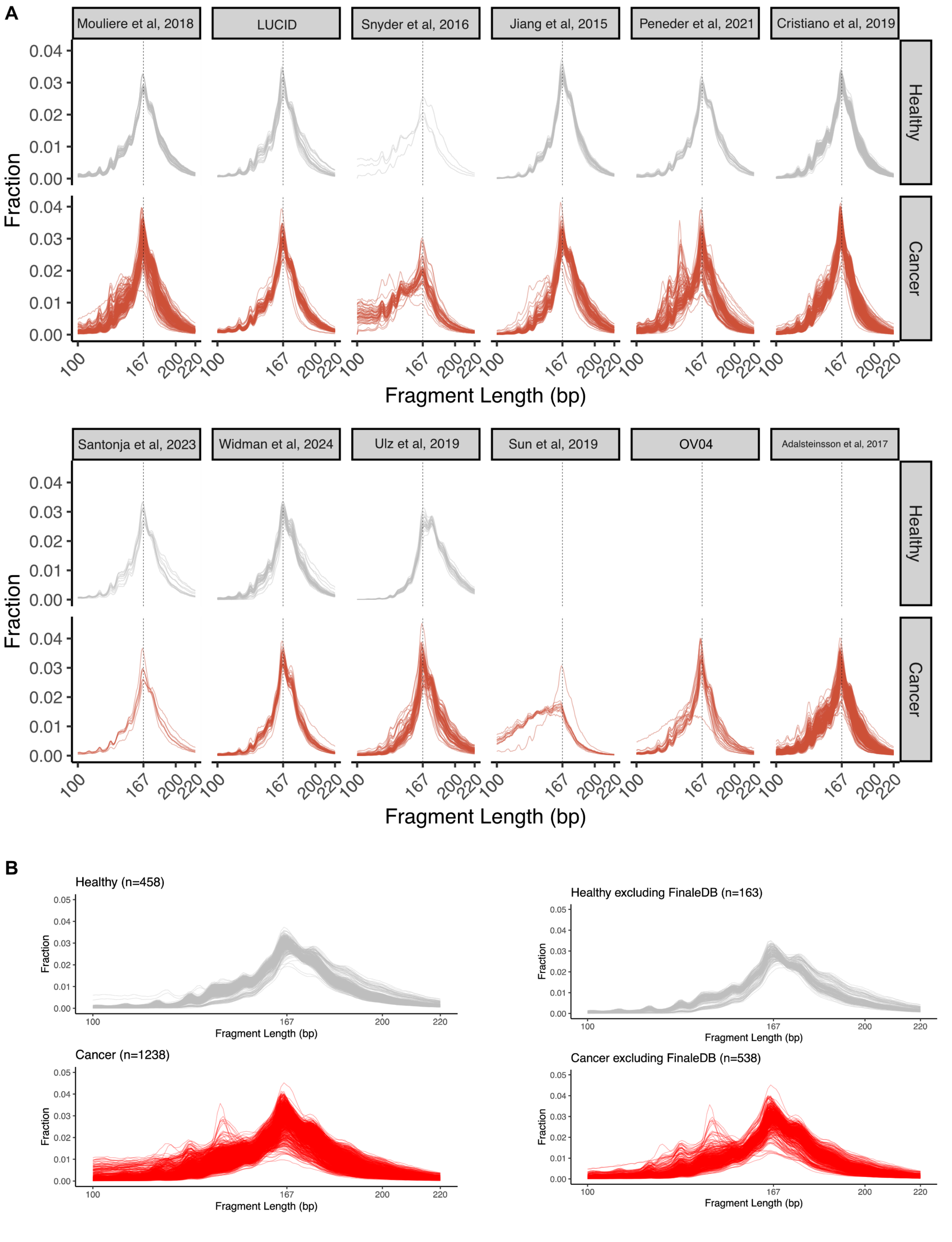

Fig. S17. Fragment length distribution of cross-validation and held-out test samples. (**A**) Distributions grouped by authors. (**B**) Distributions stratified by binary classes and database.

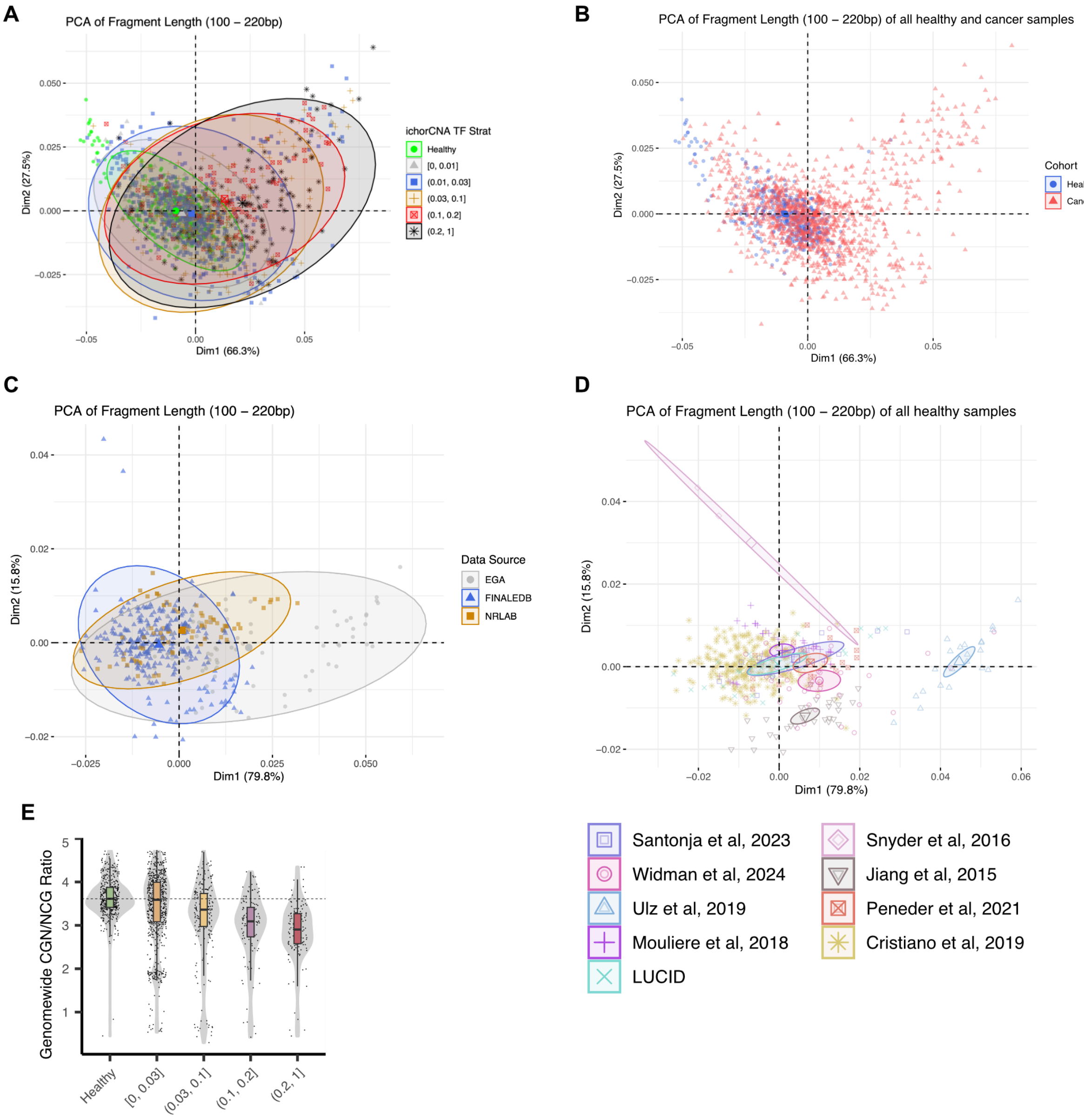

Fig. S18. Principal component analysis (PCA) of 100-220bp fragment lengths. (**A**) PCA of all samples labelled by ichorCNA TF strata. (**B**) PCA of all samples labelled by binary classes. (**C**) PCA of all healthy samples labelled by data source. (**D**) PCA of all healthy samples labelled by author. Group mean points are shown, and ellipses surrounding each cluster indicate 95% confidence area around group mean points. (**E**) Genome-wide CGN/NCG motif ratios (counting from the 5' end of each fragment). "N" denotes A, C, G or T.

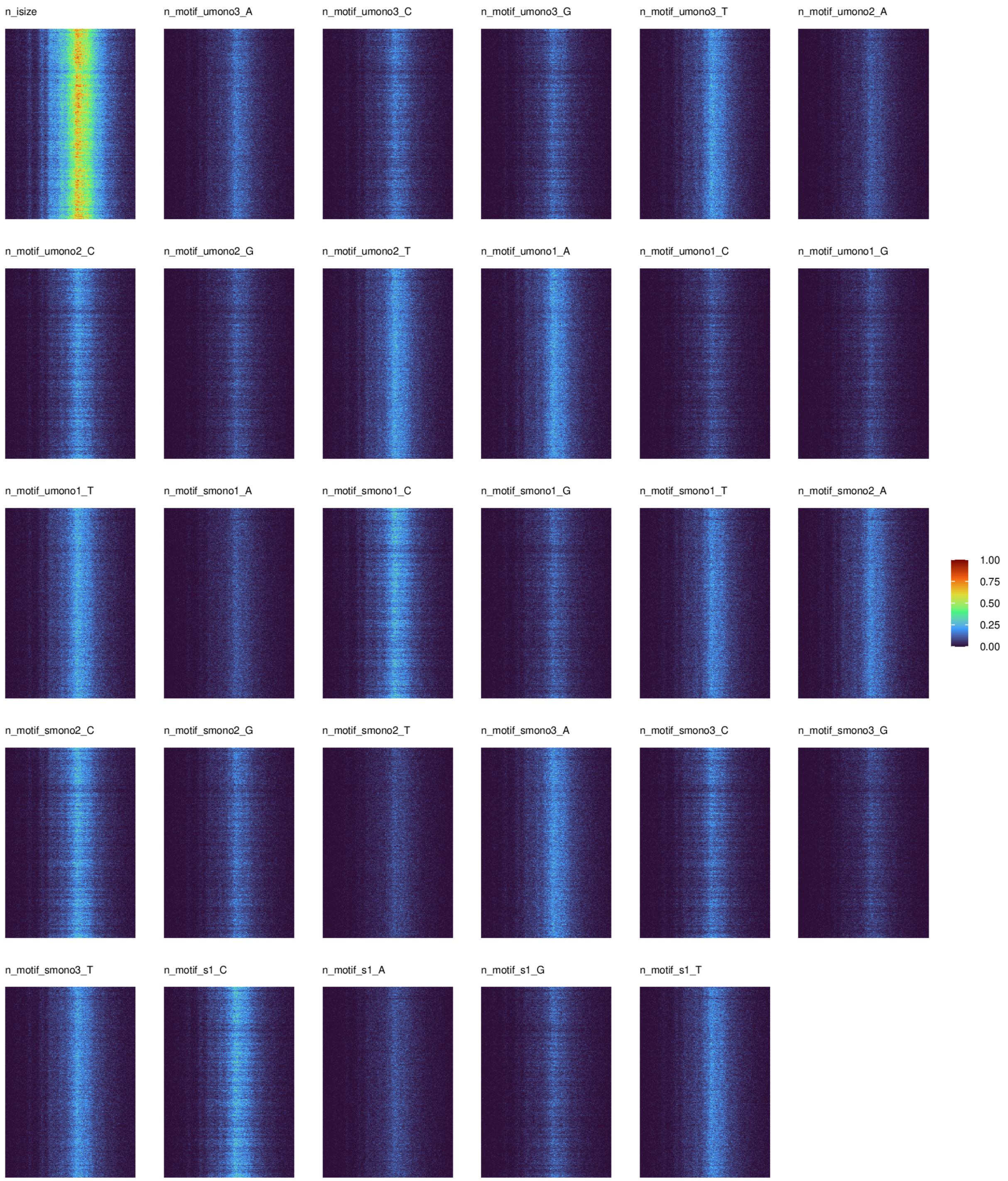

Fig. S19. Visualization of feature channels of a healthy sample. The feature channels were shown as individual heatmaps. These channels were scaled to the range of 0–1 jointly. The sample is the same as the one shown in Fig. 3B.

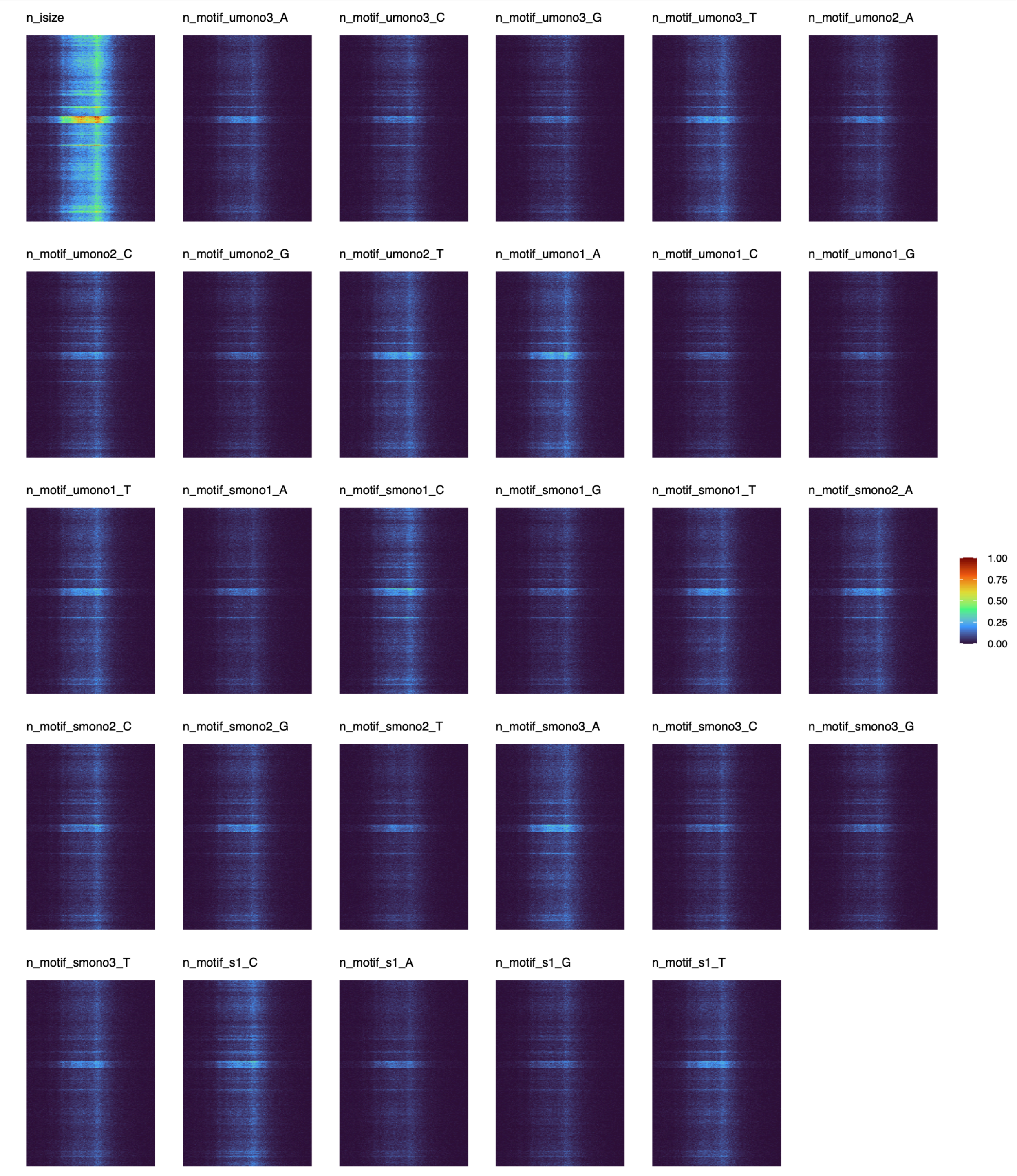

Fig. S20. Visualization of feature channels of a cancer sample. The feature channels were shown as individual heatmaps. These channels were scaled to the range of 0–1 jointly. The sample is the same as the one shown in **Fig. 3C**.

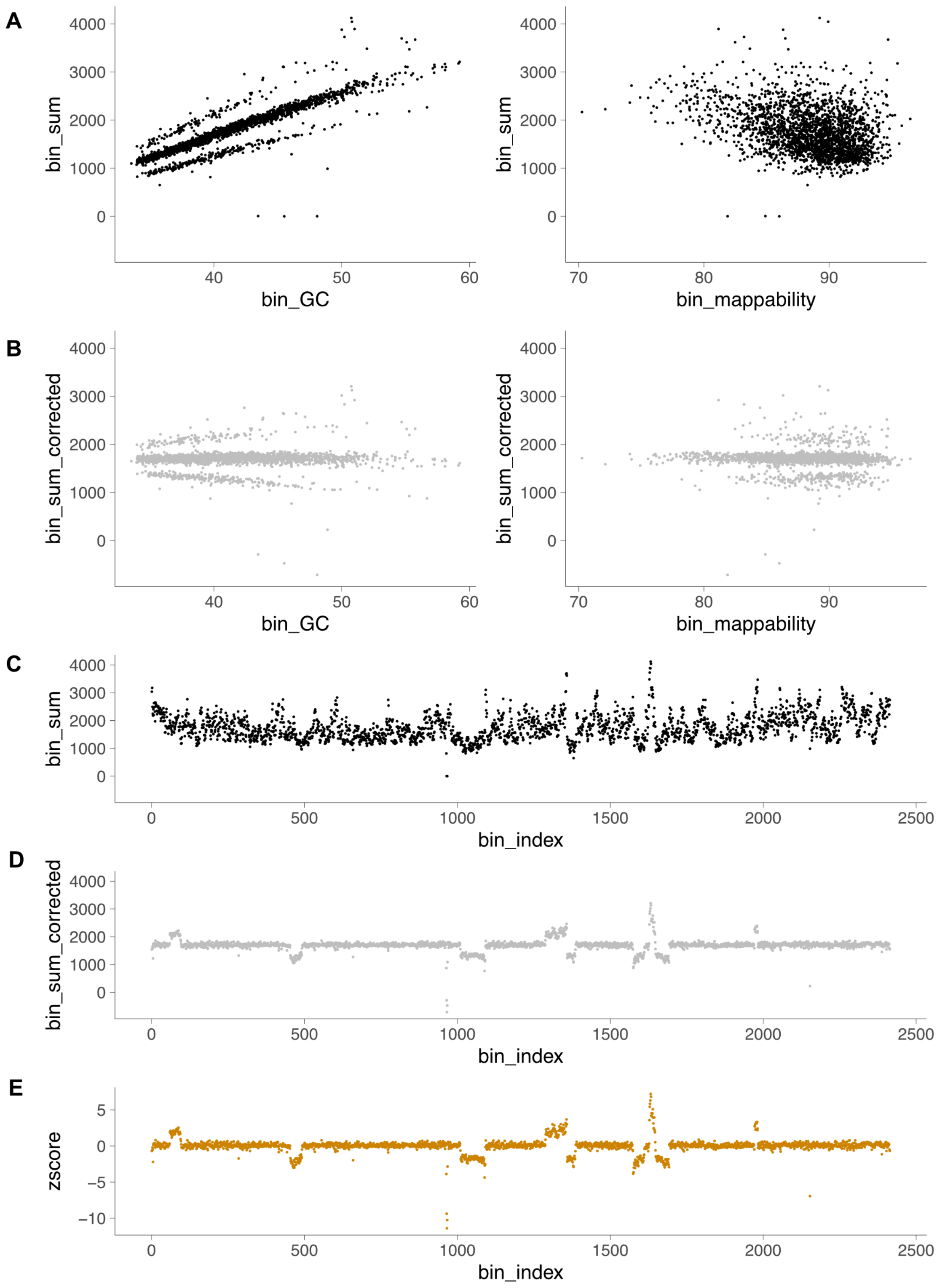

Fig. S21. An example for GC and mappability correction. (**A**) The correlation between GC content and mappability and the raw count of fragments in each bin. (**B**) The normalized count of fragments in each bin after LOESS correction for GC and mappability. (**C**) Raw count of fragments in each bin before correction. (**D**)

Normalized count of fragments in each bin after LOESS correction. (**E**) The z-score of normalized count in each bin.

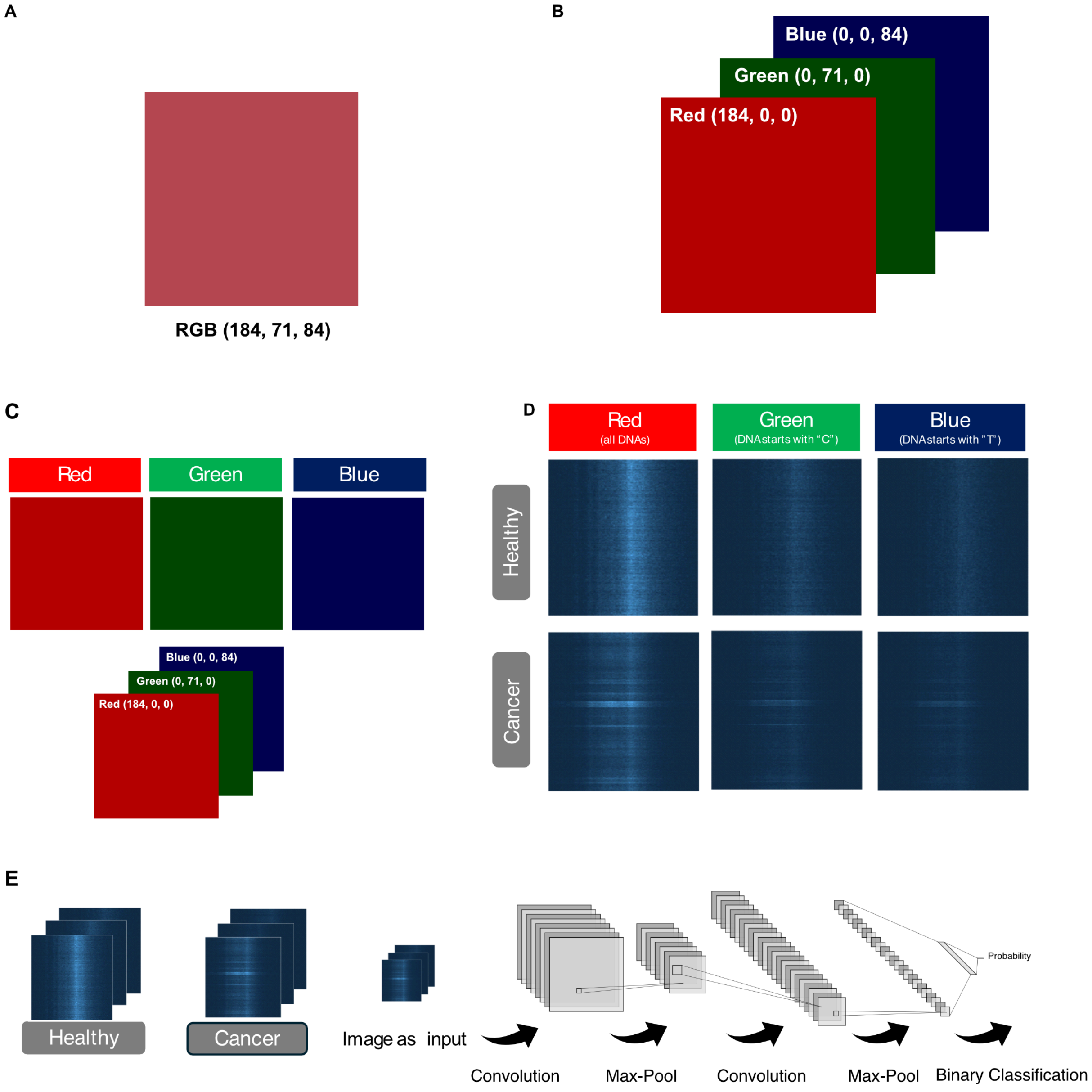

Fig. S22. Lay-style explanation of using CNN to detect cancer signal in sWGS data. (**A**) An image filled with color of RGB (184, 71, 84). (**B**) Image comprises three channels: red, green, and blue. (**C–D**) Following the same logic, the fragment length–genomic bin matrix can be visualized as an image. Each channel can be highly customized, such as the total count of fragments, only the count of fragments starting with cytosine, and only the count of fragments starting with thymine. (**E**) The matrix can then be used as input to CNN models.

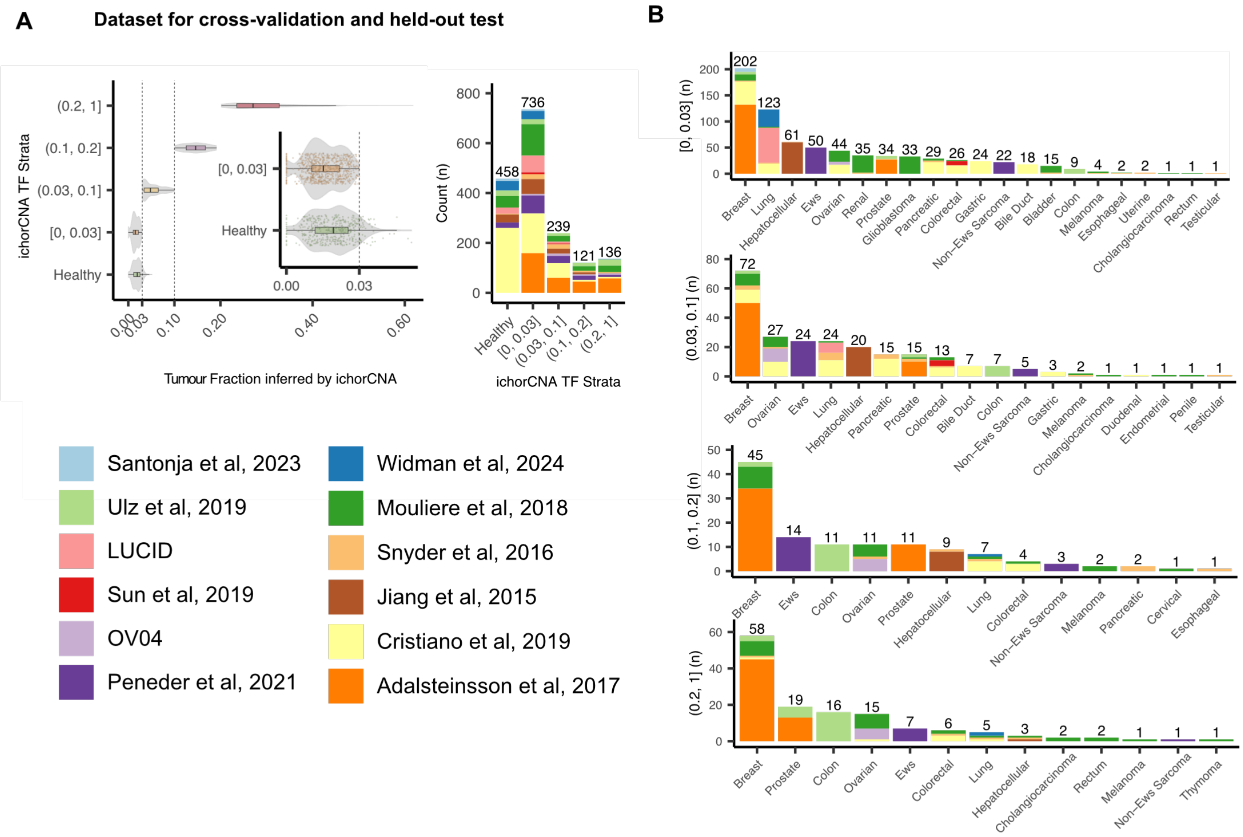

Fig. S23. Number of samples included for cross-validation and independent test. (**A**) To demonstrate the feature variation across different tumor burdens, ichorCNA Tumor Fraction (TF) is used to stratify the samples into four categories. The feature visualization is shown in **Fig. S24** and **Fig. S25**. (**B**) Cancer types in each TF category. Of note, splitting the datasets into four TF categories is only for visualization of individuals features across various level of disease severity.

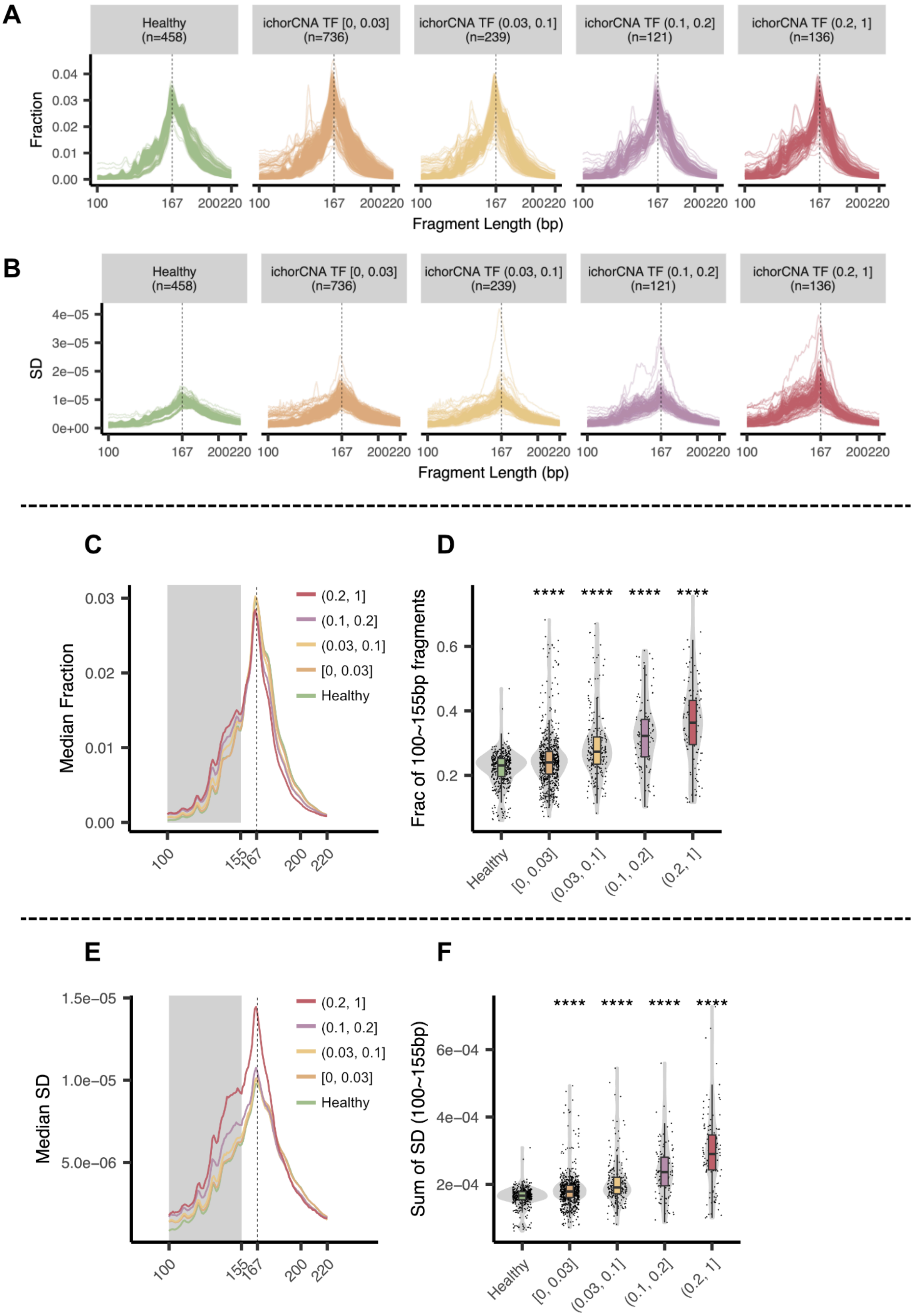

A
Healthy (n=458)
ichorCNA TF [0, 0.03] (n=736)
ichorCNA TF (0.03, 0.1] (n=239)
ichorCNA TF (0.1, 0.2] (n=121)
ichorCNA TF (0.2, 1] (n=136)
Fraction
Fragment Length (bp)
B
SD
Fragment Length (bp)
C
Median Fraction
(0.2, 1]
(0.1, 0.2]
(0.03, 0.1]
[0, 0.03]
Healthy
D
Frac of 100~155bp fragments
****
E
Median SD
F
Sum of SD (100~155bp)

Fig. S24. Comparison of Len and SD between TF categories. (**A**) Comparison between healthy and cancer classes regarding the fraction of 100–220bp fragments in different TF categories. (**B**) SD of fragment length count across genomic bins in different TF categories. (**C**) and (**D**) Median length distribution of different TF categories and the correlation of the fraction of 100–155bp fragments between individual samples and healthy median. ctDNAs are shorter than cfDNAs. (**E**) and (**F**) Median SD distribution of TF categories and the correlation of the sum of 100–155bp SD values between individual samples and healthy median. ctDNA length profiles have more variability than cfDNAs. The statistical test is based on the Mann–Whitney U test. ns: Non-significant; *: p ≤ 0.05; **: p ≤ 0.01; ***: p ≤ 0.001; ****: p ≤ 0.0001.

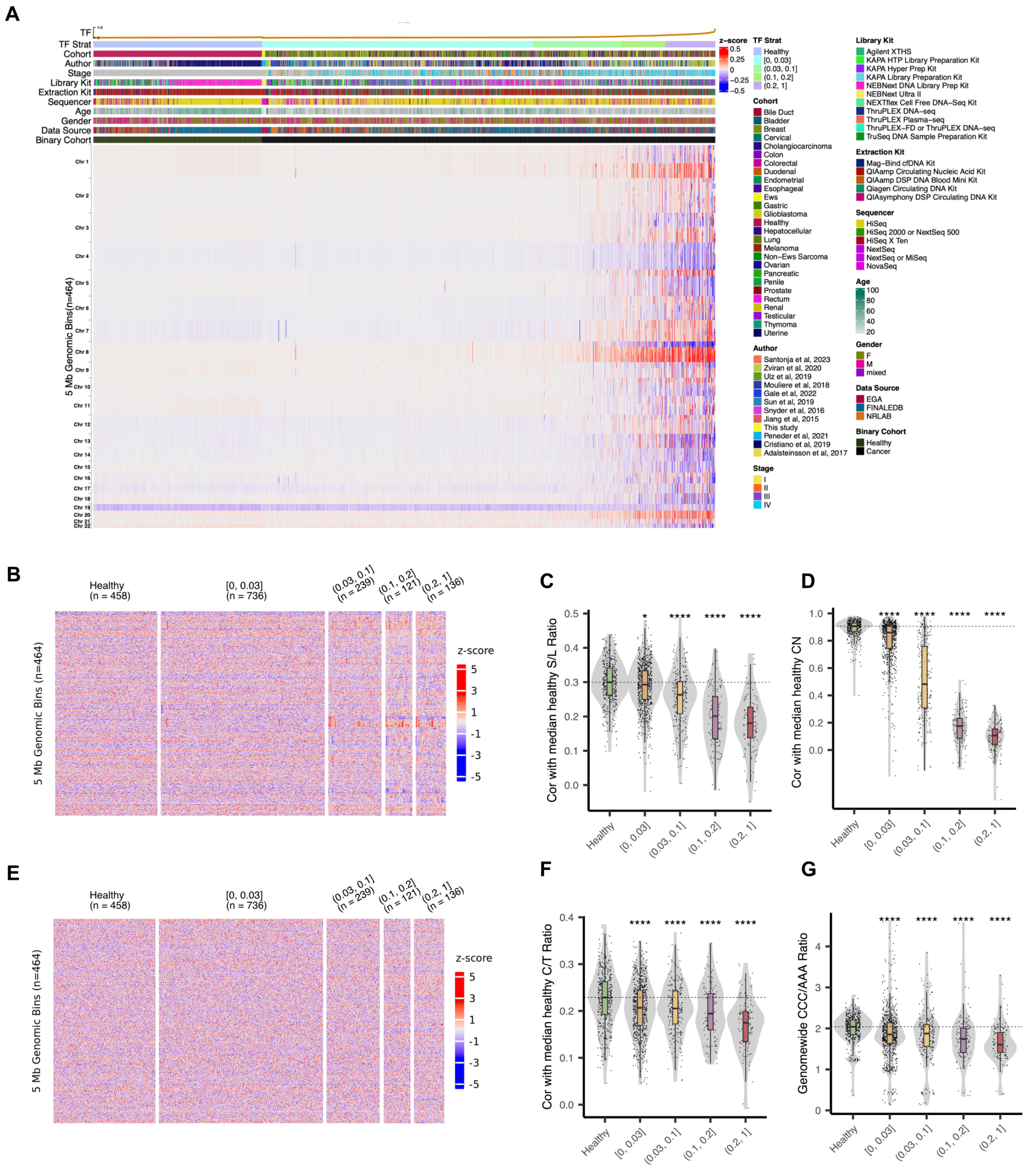

Fig. S25. Comparison of CNA, S/L and C/T between TF categories. (**A**) A heatmap showing Copy Number (CN) of all samples selected. Samples with high tumor burden have more heterogeneous CN profiles. (**B**) S/L in 5Mb genomic bins. Values in the heatmap are z-scores. (**C**) The correlation (Pearson's) with median healthy S/L. Samples with high TF exhibit more short fragments. (**D**) The correlation with median healthy Log2Ratio shown in panel (**A**). (**E**) C/T in genomic bins. (**F**) Pearson's correlation coefficients with median healthy C/T. Samples with high TF show elevated fraction of fragments starting with A or T. (**G**) Whole genome level CCC/AAA ratio. Genomic bins are 5Mb non-overlapping bins. Features derived from Chr19 were excluded from the model input. The statistical test is based on Mann–Whitney U test. ns: Non-significant; *: $p \leq 0.05$; **: $p \leq 0.01$; ***: $p \leq 0.001$; ****: $p \leq 0.0001$.

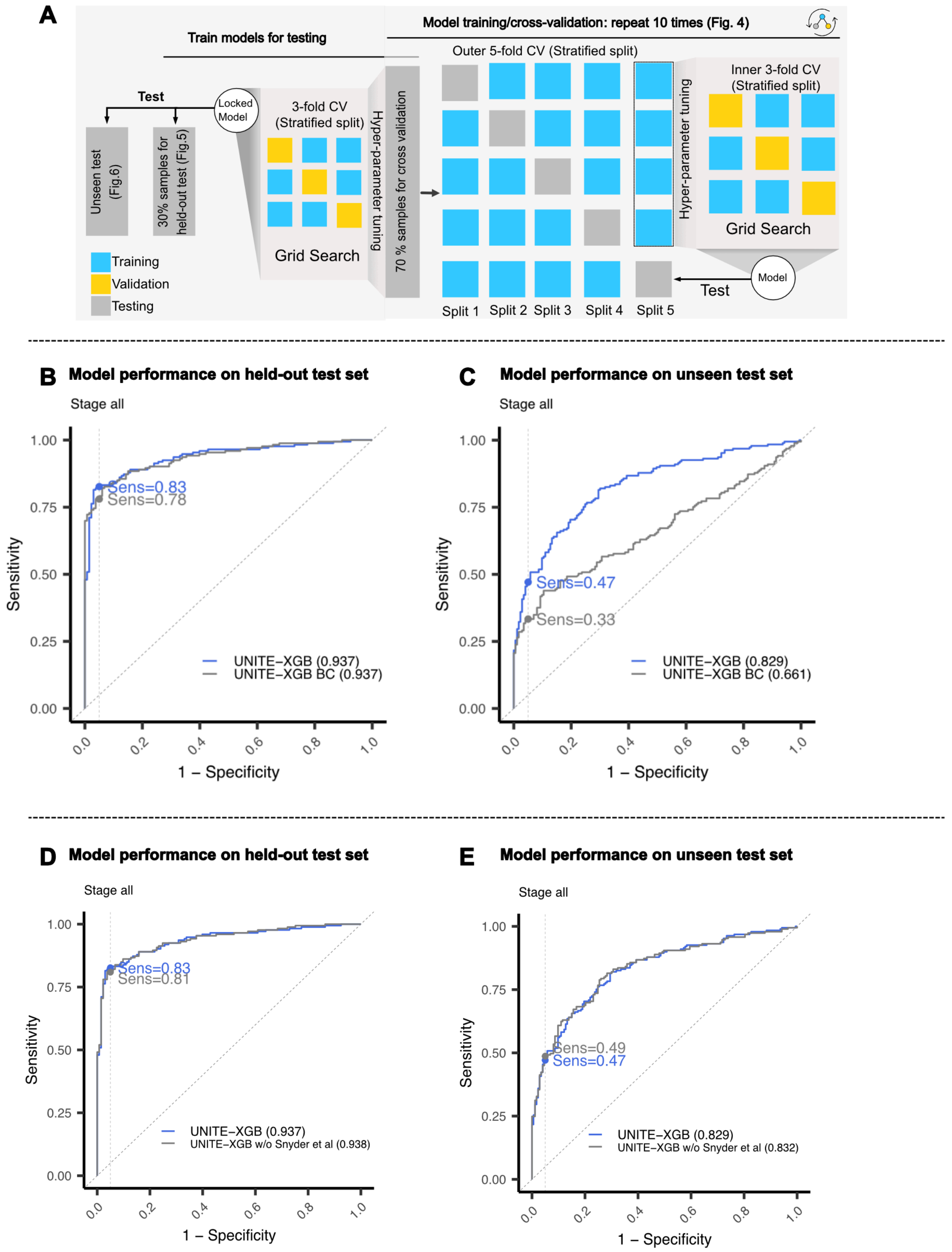

Fig. S26 Caption. Training schema and the comparison of UNITE-XGB model performances.
(**A**) The model training and testing schema. (**B–C**) Comparison of UNITE-XGB model performances with and without feature bias correction. UNITE-XGB BC denotes the UNITE-XGB model with bias correction. (**D-E**) Comparison of UNITE-XGB models trained with all samples versus models trained without samples from Snyder et al. Vertical dashed lines indicate 95% specificity.

A

Lung

Stage	I	II	III	IV	NA	Total
n	45	21	35	53	9	163

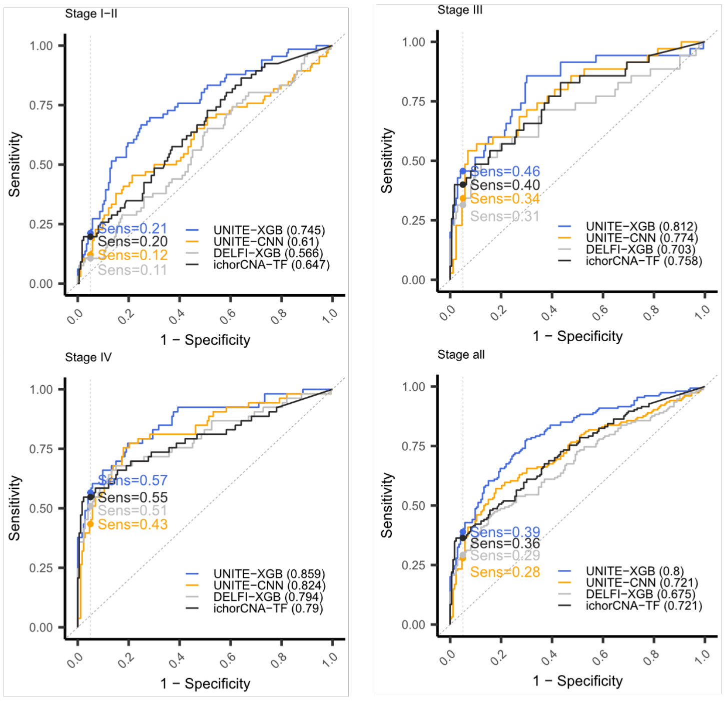

B

Breast

Stage	I	II	III	IV	NA	Total
n	4	11	5	2	2	24

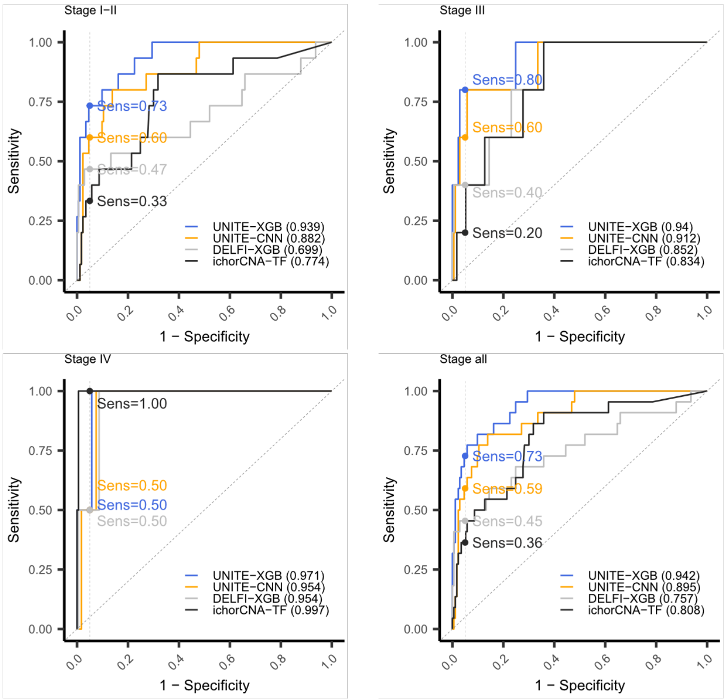

Fig. S27. Model performances in lung and breast cancer across stages in the unseen test set. (A) Lung cancer. **(B)** Breast cancer. The normal control samples used in AUC calculations are the same as in Fig. 6. Sensitivity at 95% specificity was marked on the AUC plots.

Melanoma

Stage	III	IV	Total
n	10	3	13

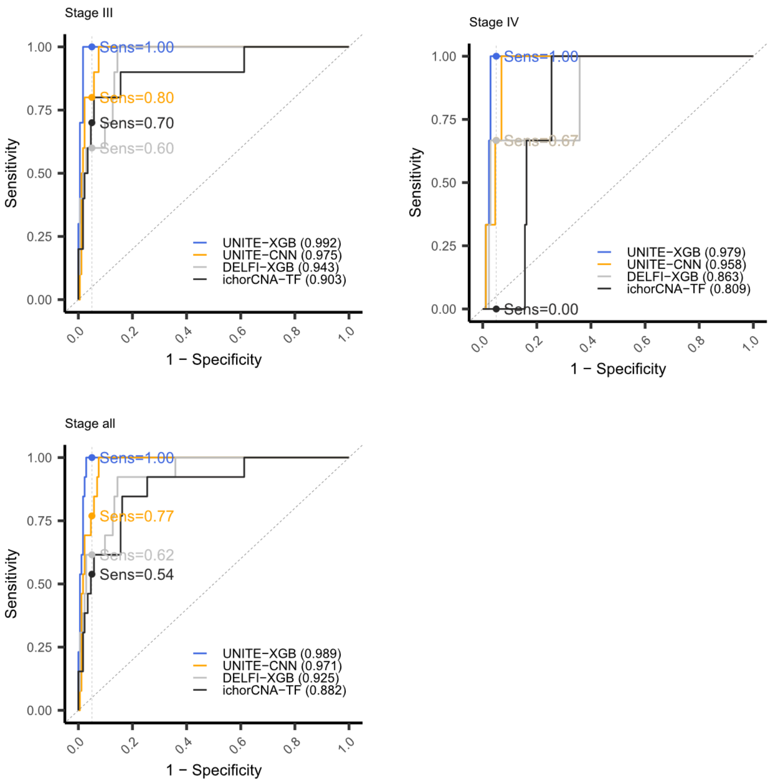

Fig. S28. Model performances in melanoma across stages in the unseen test set. The sample size in various cancer stages and the corresponding AUC curves were shown. The normal control samples used in AUC calculations are the same as in Fig. 6. Sensitivity at 95% specificity was marked on the AUC plots.

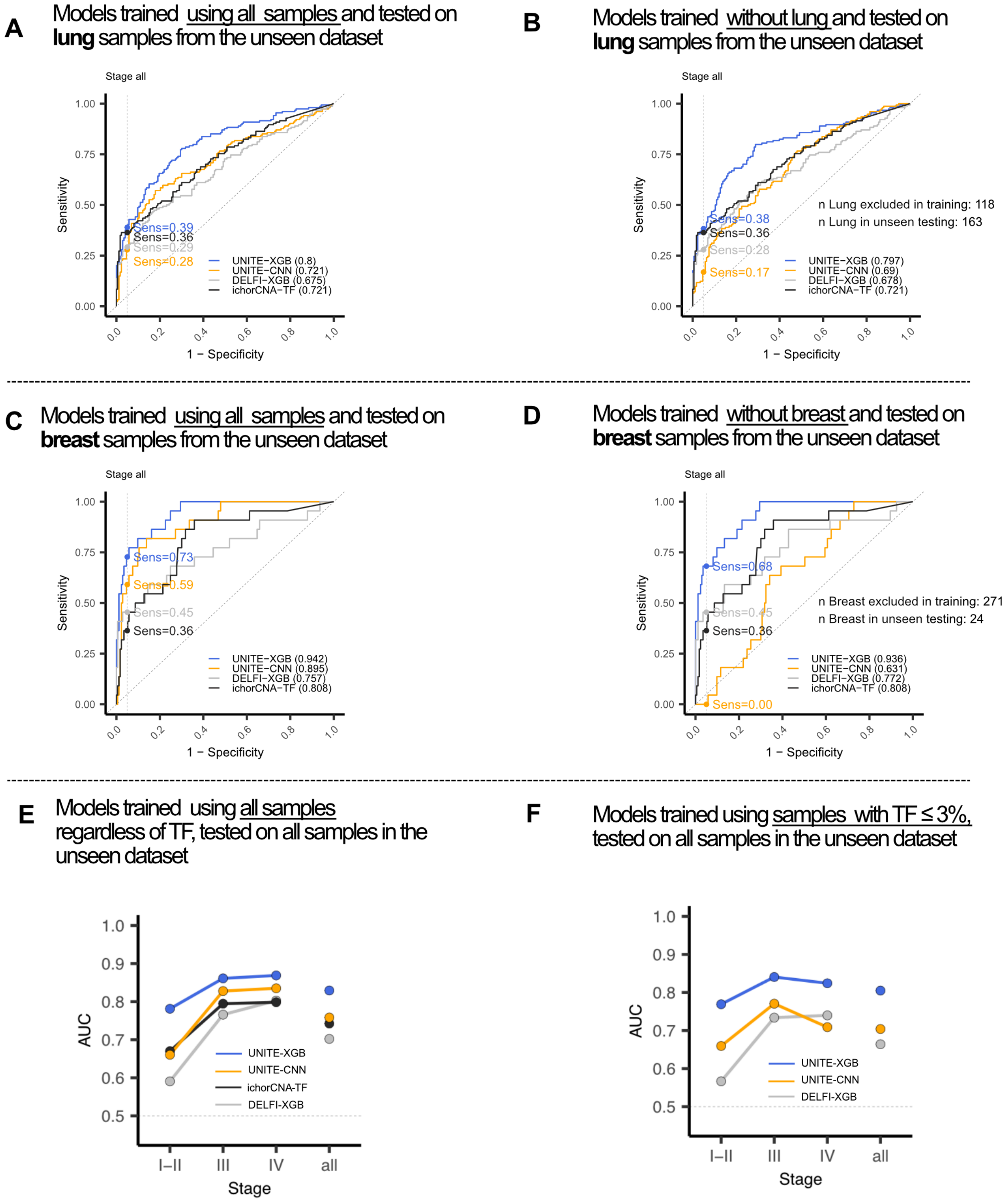

Fig. S29. Challenge the models using varying tumor fractions and cancer types between training and unseen testing. (**A**) Models trained using all cross-validation samples but tested only on lung cancer samples from the unseen test set. (**B**) Models trained using all cross-validation samples excluding lung cancer samples but tested only on lung cancer samples from the unseen test set. (**C**) Models trained using all cross-validation samples but tested only on breast cancer samples from the unseen test set. (**D**) Models

trained using all cross-validation samples excluding breast cancer samples but tested only on breast cancer samples from the unseen test set. (**E**) Models trained using all samples in the cross-validation set and tested on the entire unseen test set. (**F**) Models trained only using samples with ≤ 3 % TF and tested on the entire unseen test set. As expected, ichorCNA-TF model failed due to TF shifting, its AUCs across stages are: I-II (0.330), III (0.205), IV (0.202), all (0.258).

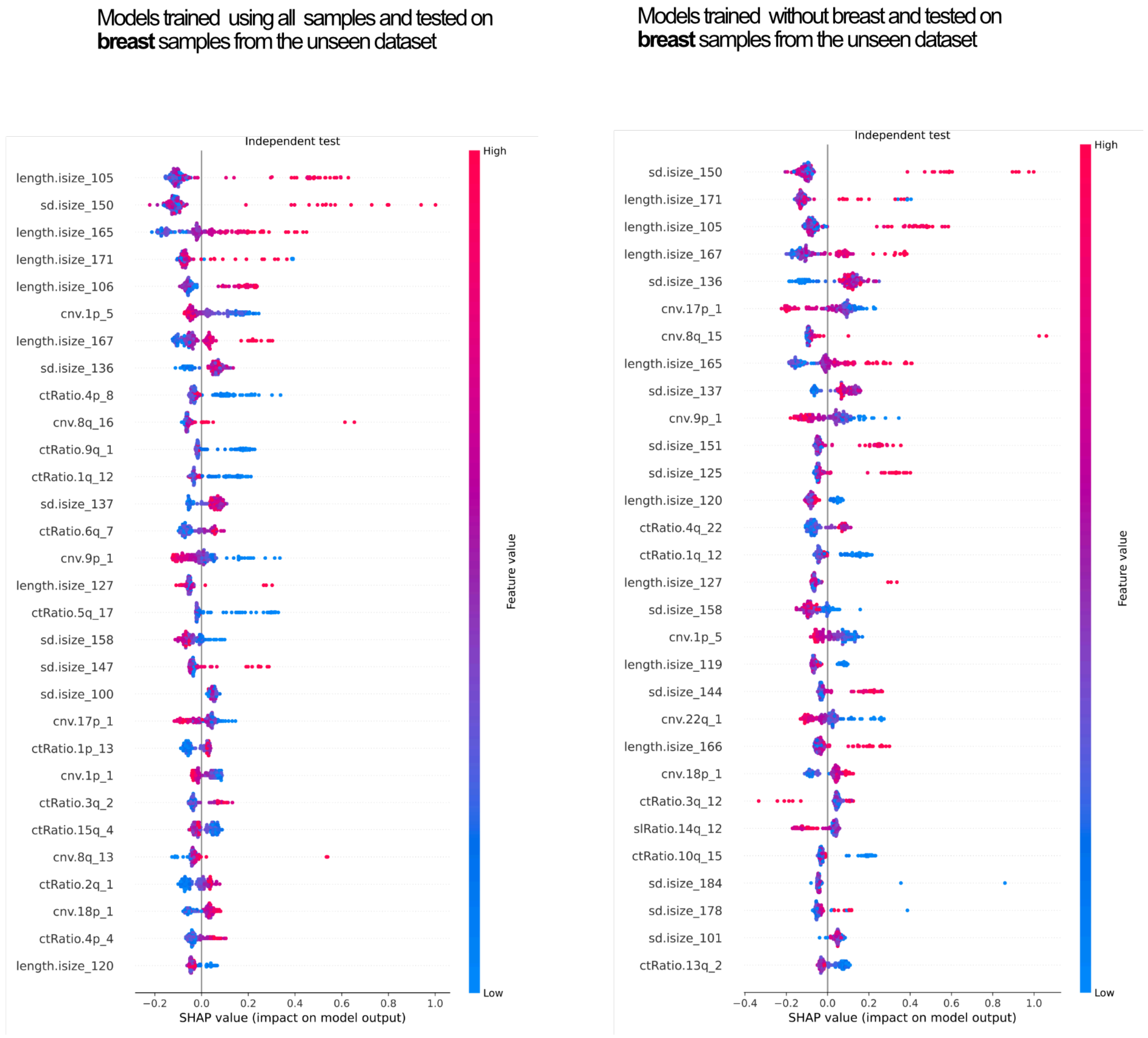

Fig. S30. Average SHAP values of model features. Models were tested on the breast samples from the unseen test set.

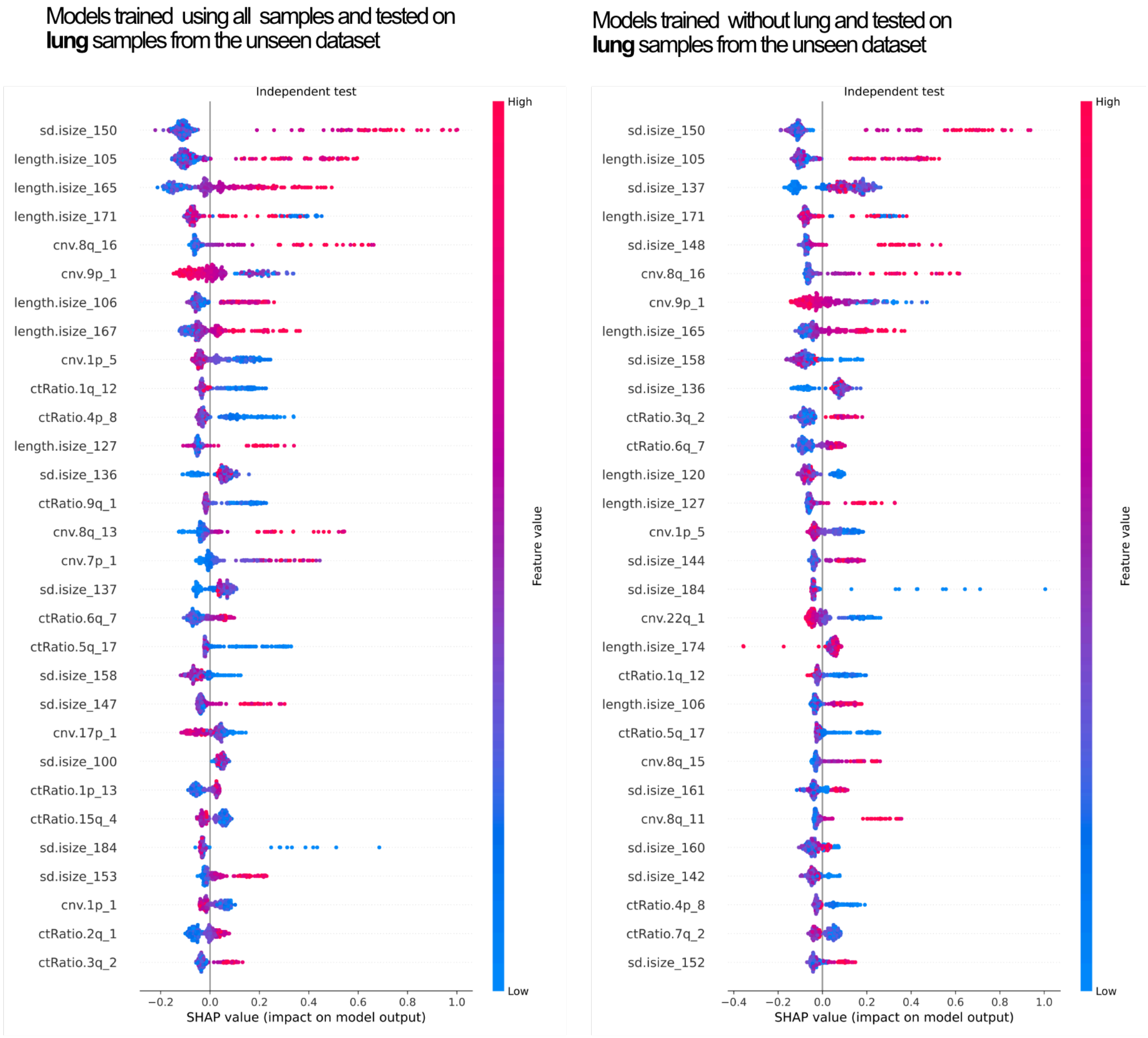

Fig. S31. Average SHAP values of model features. Models were tested on the lung samples from the unseen test set.

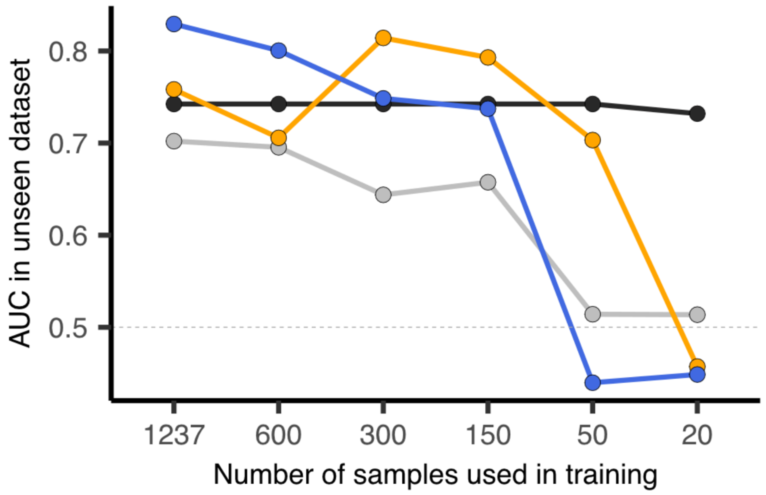

Fig. S32 Impact of reducing the number of training samples. Models were tested on the unstratified unseen test set, showing that larger training sample sizes yielded higher AUCs.

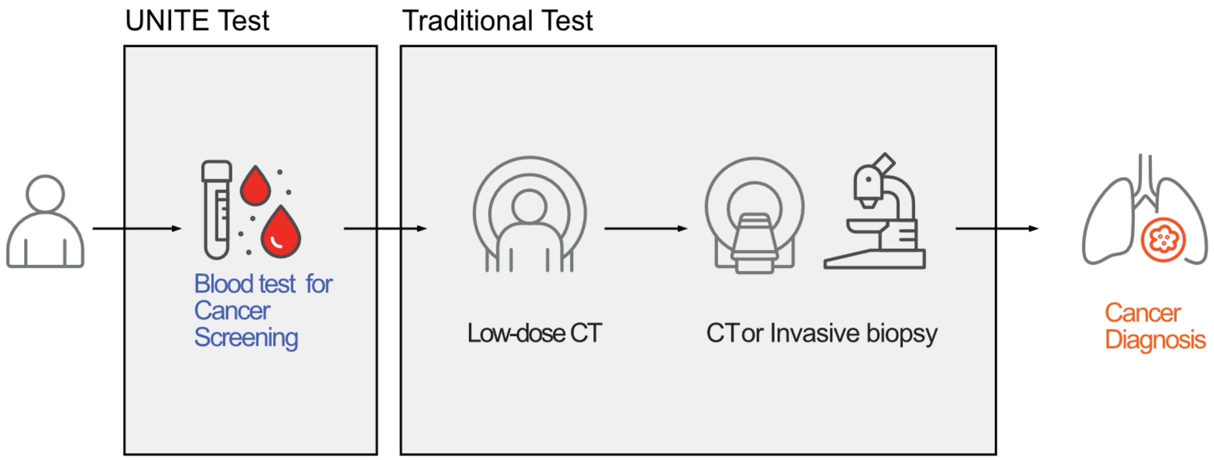

Fig. S33 Clinical pathways following a blood test result. Using lung cancer as an example, a positive blood test (e.g., UNITE test) signal would prompt further evaluations (e.g., low-dose CT) and, if indicated, diagnostic CT or biopsy to establish a diagnosis**.**

A The performance of UNITE on unseen test set at 99.6% specificity (Stage all)

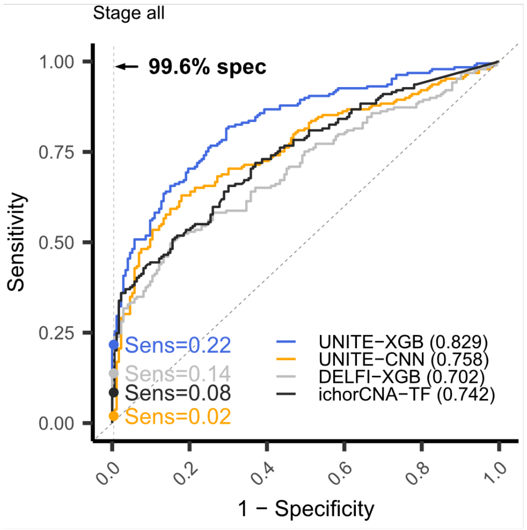

Study type: retrospective, observational

Input data: whole-genome sequencing at 0.1x

	I	II	III	IV	NA	*Row sum*
**Healthy**					173	*173*
**Lung**	45	21	35	53	9	*163*
**Breast**	4	11	5	2	2	*24*
**Melanoma**			10	3		*13*
Column sum	*49*	*32*	*50*	*58*	*184*	*373*

Fig. S34. Model performances at 99.6% specificity across all cancer stages. By fixing specificity at 99.6%, the UNITE-XGB model achieved 22% sensitivity across all stages.

A The performance of UNITE on unseen test set at 98% specificity (Stage I-II)

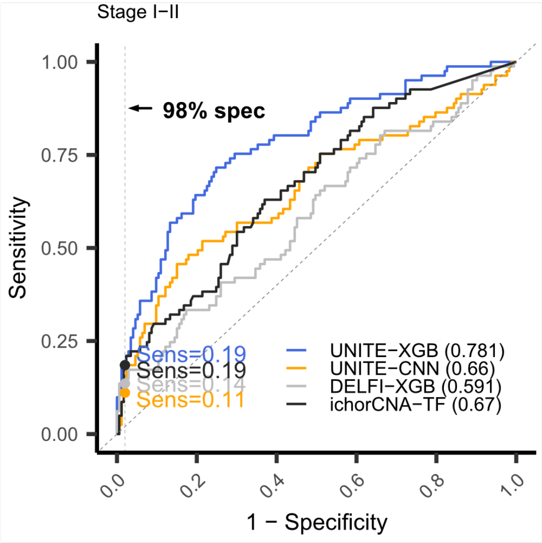

	I	II	III	IV	NA	*Row sum*
**Healthy**					173	*173*
**Lung**	45	21	35	53	9	*163*
**Breast**	4	11	5	2	2	*24*
**Melanoma**			10	3		*13*
Column sum	*49*	*32*	*50*	*58*	*184*	*373*

B The performance of other assays in the research area

	**Lung CLiP[1]**	**Galleri[2]**	**DELFI[3]**
**Features**	Mutation	Methylation	Fragmentomics
**Specificity**	0.98	0.99	0.98
**Stage I**	0.20	0.07	0.16
**Stage II**	0.29	0.30	0.17

1. Chabon et al. Nat. 2020
2. Chen et al. Clin. Cancer Res. 2021
3. Mathios et al. Nat. Commun. 2021

Fig. S35. Comparison between the performance of UNITE and other tools reported in previous studies for stage I-II cancer. (**A**) By fixing specificity at 98%, the UNITE-XGB model achieved 19% sensitivity in stage I–II cancers. (**B**) Other models' performances derived from original publications.

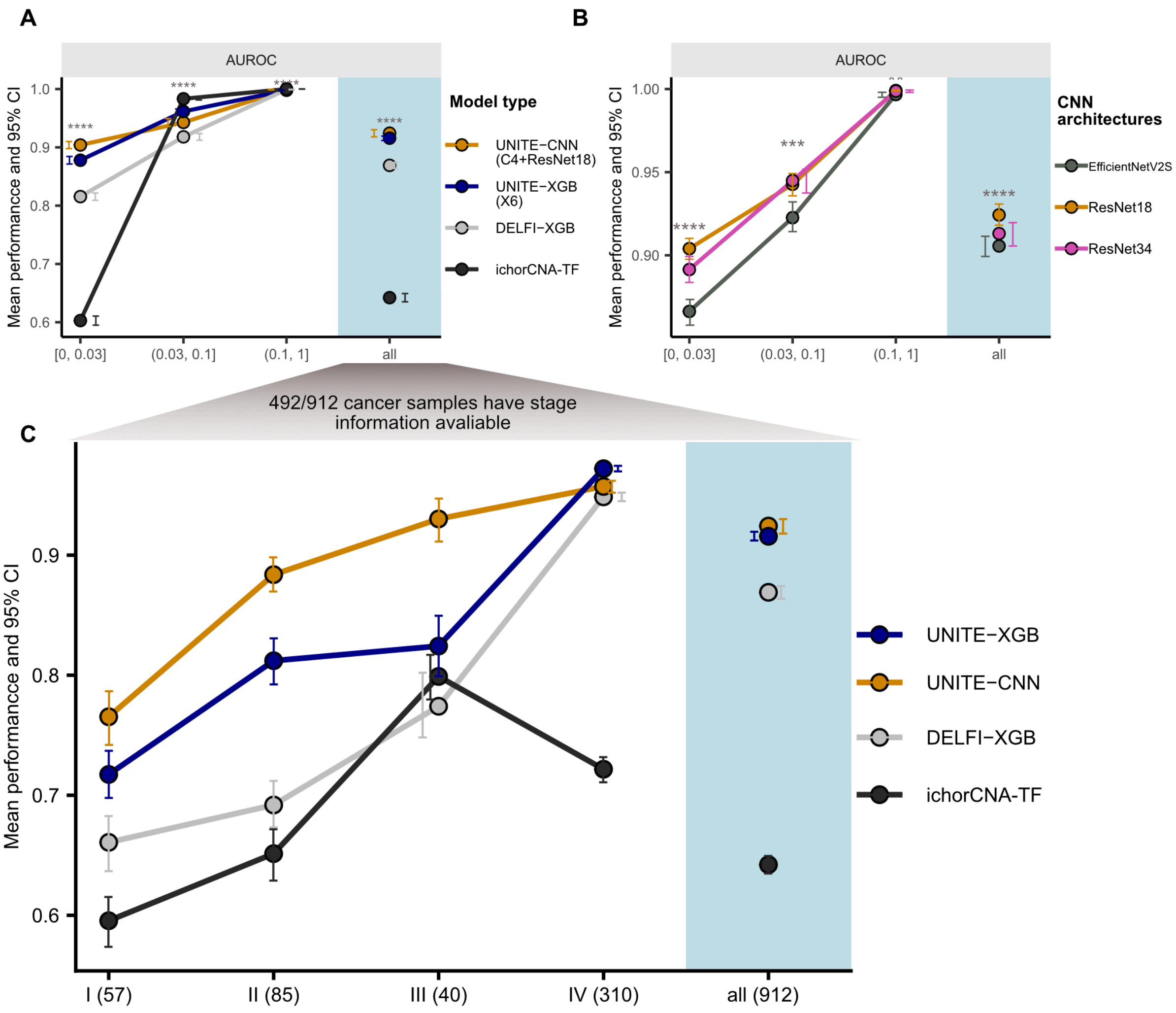

	Healthy	I	II	III	IV	NA	*Row sum*
**Cross-validation**	325	57	85	40	310	420	1237

Fig. S36. Model benchmarking results in cross-validation set (A) Comparison between XGBoost X6, ResNet18-C4 and the ichorCNA-TF model. **(B)** Performance comparison of different CNN architectures evaluated in this study. **(C)** Model performances evaluated by stratifying samples into various stages. For example, the AUC of stage I was calculated by comparing the stage I sample against healthy controls within the test fold during the 5-fold cross-validation (10 repeats). The 95%CI was calculated based on the 50 tests in total.

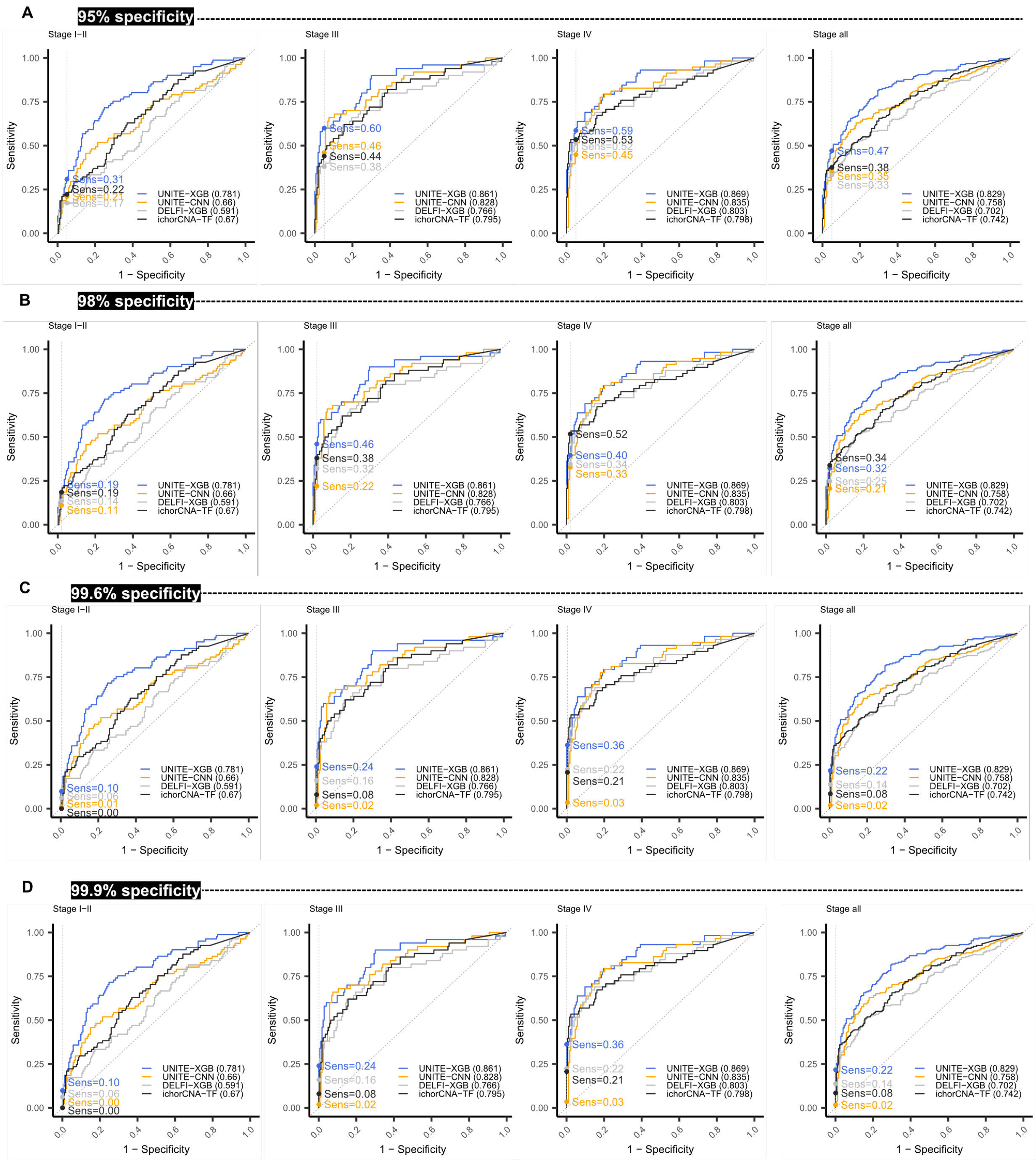

Fig. S37. Model performances at various specificity. Model performances at 95% (**A**), 98% (**B**), 99.6% (**C**), and 99.9% (**D**) specificity.

A

	I	II	III	IV	NA	*Row sum*
**Healthy**					169	*169*
**Lung**	45	21	35	53	9	*163*
**Breast**	1	5	2	2	2	*13*
**Melanoma**			10	3		*12*
Column sum	*46*	*26*	*47*	*58*	*180*	*357*

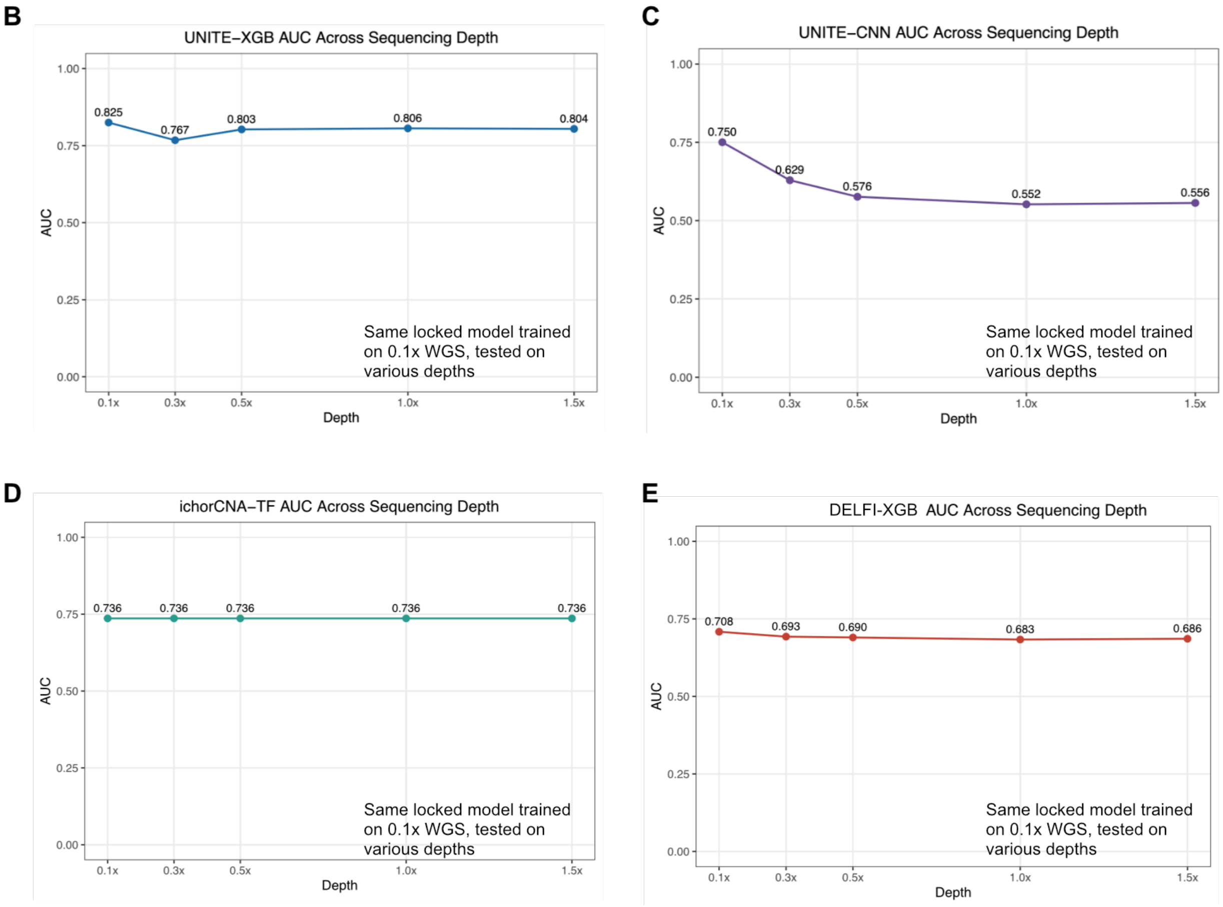

Fig. S38. Performances of the locked models trained using 0.1x data on the test set across various sequencing depths. (A) Samples used for AUC calculation. All samples were pooled for binary classification. Note that sample sizes differ from those in Figure 6 due to depth filtering and matching across different depth groups. **(B–E)** Comparison of model performance across varying sequencing depths.

Supplementary Tables

Table S1. Meta-variables of samples.

Meta variable	Explanation
cohort	The name of an abnormal condition/healthy control.

patient_id	Individual identifiers.
specimen_id	The ID of specimens (same patient can have multiple specimens).
timepoint	The time point of each specimen collection.
bam_id	The name of bam files.
study	The names of studies/authors of the datasets in Fig. 2A.
sample_type	The type of body fluids, only plasma samples were included in this study
stage	stage information.
library_kit	Library preparation kit.
extraction_kit	The DNA extraction kit.
seq_platform	Model name of the sequencer.
age	The age of the individual at specimen collection.
gender	Sex of the individual.
clinical_tf	The ground truth tumor fraction of the sample reported in publications.
data_source	The data origin.
seq_depth	The original haploid sequencing depth.
mito_frac	The fraction of mitochondrial reads in the bam file.
ichorcna_tf	The tumor fraction inferred by ichorCNA software.
ichorcna_ploidy	The ploidy inferred by ichorCNA software.
ichorcna_gender	The sex inferred by ichorCNA software.

Table S2. Metadata of cross-validation and held-out samples before quality control. Samples (n = 3430) are those shown in **Fig. 2A**. This table is provided as a separate worksheet in the Data file S1.

Table S3. The number of plasma samples selected for each cancer types from each of the studies. "Selected samples" is also shown in Fig. 2C. The number of samples allocated to cross-validation and held-out test is shown. This table is provided as a separate worksheet in the Data file S1.

Table S4. Bin annotation variables.

Columns	Description
id	Bin index number.
chromosome	The name of the chromosome to which the bin is located.
start	Bin start coordinate.
end	Bin end coordinate.
bases	Percentage of non-"N" nucleotides in this genomic bin.
gc content	Percentage of C and G nucleotides.
mappability	Average mappability of 50-mers with a maximum of 2 mismatches.
blacklist	Percent overlap with ENCODE blacklisted regions.
residual	Median LOESS residual from 1000 Genomes (50-mers).
arm	The chromosome arm name.
band	The chromosome band name.
ab	Open/Close status.

arm_bin_index	Each 100kb bin to be merged will have the same index within each arm.

Table S5. An example for 100kb bin annotations. This table is provided as a separate worksheet in the Data file S1.

Table S6. Fragment annotation variables.

Columns	Description
frag_name	The name of the fragment, i.e. read name generated by sequencer
chromosome	The name of the chromosome the fragment aligned to
start	Start coordinate of fragment
end	End coordinate of fragment
strand	Strand to which the fragment aligned to
pos_nm	The minimal distance between positive strand sequence and reference
neg_nm	The minimal distance between negative strand sequence and reference
pos_mapq	Map quality of positive-strand sequence
neg_mapq	Map quality of negative-strand sequence
pos_width	The length of sequence aligned to positive strand
neg_width	The length of sequence aligned to negative strand
pos_cigar	CIGAR of positive-strand sequence
neg_cigar	CIGAR of negative-strand sequence
frag_len	Length of fragment
frag_len_seen	Value is 1 if the fragment exists
motif_u1	Upstream 1-base motif
motif_u2	Upstream 2-base motif
motif_u3	Upstream 3-base motif
motif_s1	Start 1-base motif
motif_s2	Start 2-base motif
motif_s3	Start 3-base motif
motif_umono1	Upstream mono-base motif at position 1
motif_umono2	Upstream mono-base motif at position 2
motif_umono3	Upstream mono-base motif at position 3
motif_smono1	Start mono-base motif at position 1
motif_smono2	Start mono-base motif at position 2
motif_smono3	Start mono-base motif at position 3

Table S7. Overlap of bin and fragment annotation. Due to the large number of rows in the table (i.e., around 1 million fragments per sample with a depth of 0.1x), a truncated version is provided as a separate worksheet in the Data file S1 for simplicity.

Table S8. Sample size of control and each cancer type in the data split. Square brackets indicate inclusion of boundaries. The EWS: Ewing Sarcoma.

	**[0, 0.03]**		**(0.03, 0.1]**		**(0.1, 1]**		**all**	
	Cross-validation	Held-out Test	Cross-validation	Held-out Test	Cross-validation	Held-out Test	Cross-validation	Held-out Test
Healthy	325	133	325	133	325	133	325	133
Uterine	2	0	0	0	0	0	2	0
Thymoma	0	0	0	0	1	0	1	0
Testicular	1	0	1	0	0	0	2	0
Renal	26	9	0	0	0	0	26	9
Rectum	1	0	0	0	2	0	3	0
Prostate	25	9	12	3	22	8	59	20
Penile	0	0	1	0	0	0	1	0
Pancreatic	22	7	12	3	2	0	36	10
Ovarian	32	12	20	7	20	6	72	25
Non-EWS Sarcoma	16	6	4	1	3	1	23	8
Melanoma	4	0	2	0	3	0	9	0
Lung	89	34	18	6	11	1	118	41
Hepatocellular	43	18	14	6	10	2	67	26
Glioblastoma	24	9	0	0	0	0	24	9
Gastric	17	7	3	0	0	0	20	7
EWS	35	15	17	7	15	6	67	28
Esophageal	2	0	0	0	1	0	3	0
Endometrial	0	0	1	0	0	0	1	0
Duodenal	0	0	1	0	0	0	1	0
Colorectal	20	6	11	2	9	1	40	9
Colon	7	2	5	2	19	8	31	12
Cholangiocarcinoma	1	0	1	0	2	0	4	0
Cervical	0	0	0	0	1	0	1	0
Breast	144	58	53	19	74	29	271	106
Bladder	12	3	0	0	0	0	12	3
Bile Duct	13	5	5	2	0	0	18	7
Total	861	333	506	191	520	195	1237	453

Table S9. The version numbers of software, packages, modules, and tools used in the data pre-processing steps. The upper section details the software employed by the Trim Align Pipeline (TAP), developed by NRLAB, for sequencing data trimming and alignment, while the lower section outlines the R packages used in subsequent analysis steps, including bin and fragment filtering, annotation, and visualisation.

**Software/Package**	**Version**	**Source**
Software used in TAP		
miniconda	py39_4.12.0	anaconda.com
python	3.6	conda-forge
mysql-connector-python	8	conda-forge
bamtools	2.5.2	bioconda
bedtools	2.30.0	bioconda
biopython	1.7	bioconda
bowtie2	2.5.1	bioconda
bwa-mem2	2.2.1	bioconda
bwameth	0.2.6	bioconda
fastqc	0.11.9	bioconda
gatk4	4.2.6.1	bioconda
hmmcopy	0.1.1	bioconda
picard	2.27.4	bioconda
pysam	0.19	bioconda
samtools	1.15.1	bioconda
trim-galore	0.6.7	bioconda
trimmomatic	0.39	bioconda
r-base	4.1.3	conda-forge
r-optparse	1.7.3	conda-forge
r-tidyverse	1.3.2	conda-forge
r-ichorcna	0.3.2	bioconda
groovy	4.0.15	groovy.jfrog.io
AGeNT	3.0.5	agilent.com
connor	0.6.1	bioconda
pysam	0.11.2.2	bioconda
**R Package**		
R	4.2.3	CRAN
cfDNAPro	1.7.1	github.com/nrlab-CRUK/cfDNAPro
factoextra	1.0.7	CRAN
ggalluvial	0.12.5	CRAN
parallel	4.2.3	CRAN
future	1.33.2	CRAN
furrr	0.3.1	CRAN
progressr	0.14.0	CRAN
parallelly	1.37.1	CRAN
tidyverse	1.3.2	CRAN
Biobase	2.58.0	Bioconductor
QDNAseq	1.30.0	Bioconductor
Homo.sapiens	1.3.1	Bioconductor
plyranges	1.18.0	Bioconductor
GenomicRanges	1.50.0	Bioconductor
GenomicAlignments	1.34.0	Bioconductor
matrixStats	0.63.0	CRAN
AnnotationHub	3.6.0	Bioconductor
BSgenome.Hsapiens.UCSC.hg19	1.4.3	Bioconductor
Rsamtools	2.14.0	Bioconductor
rslurm	0.6.2	CRAN
abind	1.4-5	CRAN

Table S10. Software and Python modules used in modelling.

Software/Python module	Version	Source
singularity-ce	3.11.5-1.el8	github.com/sylabs/singularity
Python	3.11.0rc1	Docker image obtained on Wednesday, 15th May 2024 at 22:13:28 (UTC), version number of 22.04: hub.docker.com
cuda	12.3.0	Python Package Index
sklearn	1.4.2	Python Package Index
keras	3.3.3	Python Package Index
numpy	1.26.4	Python Package Index
pandas	2.2.2	Python Package Index
skorch	1.0.7	Python Package Index
timm	1.0.0	Python Package Index

Table S11. **Running the pipeline.** Step-by-step guidance of preprocessing, model training, and testing. Code snippets for each step were shown on GitHub: https://github.com/nrlab-CRUK/UNITE-paper. CPU used in the study: EPYC™ 7452 CPU processors (32 cores) @ 2.2 GHz. GPU: NVIDIA® L40S GPU cards. The running time refers to single-sample processing time unless otherwise specified.

Step	Explanation	Hardware	Running time
1. Index BAM files.	Generate BAM index files if needed	CPU	< 1 min per BAM
2. Calculate descriptive statistics of BAMs	e.g., sequencing depth, fraction of mitochondrial fragments	CPU	< 10 min per BAM (2–5× depth)
3. Downsample BAM to target depth	Target depth = 0.1× in this study	CPU	< 10 min per BAM (2–5× depth, target 0.1×)
4. Run UNITE on each BAM file	BAMs split into 2 million-read chunks and processed in parallel.	CPU	≈ 1 h per 0.1× BAM on single CPU
5. Generate UNITE plots	e.g., plot shown in Fig. S19.	CPU	< 1 min
6. Quality-control UNITE results	Quality-check the generated files	CPU	< 1 min
7. Build MAE object and extract model inputs from MAE	For reproducible science	CPU	Building MAE for 600 samples ≈ 25 min (CPU); extracting CNN/XGBoost features ≈ 30 min / < 1 min
8. Model training	Training ≈ 1200 samples	XGBoost on CPU; CNN on GPU	UNITE-CNN ≈ 20 min (GPU, fine-tuning); UNITE-XGBoost ≈ 20 h (3-fold cross-validation tuning)
9. Model testing	Test on independent unseen dataset	XGBoost on CPU; CNN on GPU	UNITE-XGB (~2 MB): < 10 s for 400 samples; UNITE-CNN (~260 MB): < 30 s for 400 samples

Table 12. Sample sizes of various types allocated to cross-validation, held-out and unseen test set. The entire cross-validation set was used for training the locked models, which were then tested on the held-out and unseen test set.

	**Cross-validation**	**Held-out test**	**Unseen test**
Healthy	325	133	173
Breast	271	106	24
Lung	118	41	163
Ovarian	72	25	
Hepatocellular	67	26	
EWS	67	28	
Prostate	59	20	
Colorectal	40	9	
Pancreatic	36	10	
Colon	31	12	
Renal	26	9	
Glioblastoma	24	9	
Non-EWS Sarcoma	23	8	
Gastric	20	7	
Bile Duct	18	7	
Bladder	12	3	
Melanoma	9		13
Cholangiocarcinoma	4		
Rectum	3		
Esophageal	3		
Uterine	2		
Testicular	2		
Thymoma	1		
Penile	1		
Endometrial	1		
Duodenal	1		
Cervical	1		
**Total**	**1237**	**453**	**373**

Table S13. Metadata of the finalized cross-validation, held-out and unseen test samples. Samples (n = 2063) are those shown in **Fig. 2C-D** and **Table S12**. This table is provided as a separate worksheet in the **Data file S1**. Note that the metadata for samples collated for cross-validation and held-out test before quality control (n = 3430, Fig. 2A) are recorded in **Table S2**.

Data file S1: **Excel spread sheets containing the following tables: Table S2, S3, S5, S7 and S13.**
Table S2 shows metadata of cross-validation and held-out samples before quality control. Samples (n = 3430) are those shown in Fig. 2A. Table S3 shows the number of plasma samples selected for each cancer types from each of the studies. "Selected samples" is also shown in Fig. 2C. The number of samples allocated to cross-validation and held-out test is shown. Table S5 shows an example for 100kb bin annotations. Each column represents names, coordinates or other annotations of genomic bins. Table S7 shows overlap of bin and fragment annotation. Due to the large number of rows in the table (i.e., around 1 million fragments per sample with a depth of 0.1x), a truncated version is provided for simplicity. Table S13 shows metadata of the finalized cross-validation, held-out and unseen test samples. Samples (n = 2063) are those shown in Fig. 2C-D and Table S12.

Data file S2: **Excel spread sheets containing the raw model scores or summary statistics for held-out test and unseen test.**
Sheet "Table of Contents" summarizes the contents of each worksheet in this file. Sheet "STATS_lr" shows the summary statistics of ichorCNA-TF models trained in different tumour fraction categories, while sheet "RAW_lr" provides the raw performance results of the ichorCNA-TF model. Sheet "STATS_xgb_x1-x6" summarizes the performance of XGBoost models x1 to x6 during cross-validation (5-fold cross-validation with 10 repeats), and sheet "RAW_xgb_x1-x6" provides the corresponding raw cross-validation results. Sheet "STATS_xgb_all_feat" shows the summary statistics of the UNITE-XGB model during cross-validation, while sheet "RAW_xgb_all_feat" reports the corresponding UNITE-XGB cross-validation performance. Sheet "STATS_cnn_c1-c5" summarizes the performance of different CNN models and architectures, and sheet "RAW_cnn_c1-c5" provides the raw cross-validation performance of CNN models c1 to c6. Sheet "locked model scores on held-out and unseen test set" contains the original prediction scores generated by the locked models on the held-out and unseen test samples used in Fig. 5 and Fig. 6.

REFERENCES

1. J. C. M. Wan, C. Massie, J. Garcia-Corbacho, F. Mouliere, J. D. Brenton, C. Caldas, S. Pacey, R. Baird, N. Rosenfeld, Liquid biopsies come of age: Towards implementation of circulating tumour DNA. *Nat. Rev. Cancer* **17**, 223–238 (2017).

2. A. R. Thierry, S. El Messaoudi, P. B. Gahan, P. Anker, M. Stroun, Origins, structures, and functions of circulating DNA in oncology. *Cancer Metastasis Rev.* **35**, 347–376 (2016).

3. F. Mouliere, C. G. Smith, K. Heider, J. Su, Y. van der Pol, M. Thompson, J. Morris, J. C. M. Wan, D. Chandrananda, J. Hadfield, M. Grzelak, I. Hudecova, D. Couturier, W. Cooper, H. Zhao, D. Gale, M. Eldridge, C. Watts, K. Brindle, N. Rosenfeld, R. Mair, Fragmentation patterns and personalized sequencing of cell-free DNA in urine and plasma of glioma patients. *EMBO Mol. Med.* **13**, e12881 (2021).

4. F. Mouliere, A hitchhiker's guide to cell-free DNA biology. *Neurooncol. Adv.* **4**, ii6–ii14 (2022).

5. P. Jiang, Y. M. D. Lo, The long and short of circulating cell-free DNA and the ins and outs of molecular diagnostics. *Trends Genet.* **32**, 360–371 (2016).

6. Y. M. Dennis Lo, N. Corbetta, P. F. Chamberlain, V. Rai, I. L. Sargent, C. W. G. Redman, J. S. Wainscoat, Presence of fetal DNA in maternal plasma and serum. *Lancet* **350**, 485–487 (1997).

7. P. Burnham, D. Dadhania, M. Heyang, F. Chen, L. F. Westblade, M. Suthanthiran, J. R. Lee, I. De Vlaminck, Urinary cell-free DNA is a versatile analyte for monitoring infections of the urinary tract. *Nat. Commun.* **9**, 2412 (2018).

8. A. M. Newman, S. V. Bratman, J. Tong, J. F. Wynne, N. C. W. Eclov, L. A. Modlin, C. L. Liu, J. W. Neal, H. A. Wakelee, R. E. Merritt, J. B. Shrager, B. W. Loo, A. A. Alizadeh, M. Diehn, An ultrasensitive method for quantitating circulating tumor DNA with broad patient coverage. *Nat. Med.* **20**, 548–554 (2014).

9. B. R. McDonald, T. Contente-Cuomo, S.-J. Sammut, A. Odenheimer-Bergman, B. Ernst, N. Perdigones, S.-F. Chin, M. Farooq, R. Mejia, P. A. Cronin, K. S. Anderson, H. E. Kosiorek, D. W. Northfelt, A. E. McCullough, B. K. Patel, J. N. Weitzel, T. P. Slavin, C. Caldas, B. A.

Pockaj, M. Murtaza, Personalized circulating tumor DNA analysis to detect residual disease after neoadjuvant therapy in breast cancer. *Sci. Transl. Med.* **11**, eaax7392 (2019).

10. J. C. M. Wan, K. Heider, D. Gale, S. Murphy, E. Fisher, F. Mouliere, A. Ruiz-Valdepenas, A. Santonja, J. Morris, D. Chandrananda, A. Marshall, A. B. Gill, P. Y. Chan, E. Barker, G. Young, W. N. Cooper, I. Hudecova, F. Marass, R. Mair, K. M. Brindle, G. D. Stewart, J. E. Abraham, C. Caldas, D. M. Rassl, R. C. Rintoul, C. Alifrangis, M. R. Middleton, F. A. Gallagher, C. Parkinson, A. Durrani, U. McDermott, C. G. Smith, C. Massie, P. G. Corrie, N. Rosenfeld, ctDNA monitoring using patient-specific sequencing and integration of variant reads. *Sci. Transl. Med.* **12**, eaaz8084 (2020).

11. A. Zviran, R. C. Schulman, M. Shah, S. T. K. Hill, S. Deochand, C. C. Khamnei, D. Maloney, K. Patel, W. Liao, A. J. Widman, P. Wong, M. K. Callahan, G. Ha, S. Reed, D. Rotem, D. Frederick, T. Sharova, B. Miao, T. Kim, G. Gydush, J. Rhoades, K. Y. Huang, N. D. Omans, P. O. Bolan, A. H. Lipsky, C. Ang, M. Malbari, C. F. Spinelli, S. Kazancioglu, A. M. Runnels, S. Fennessey, C. Stolte, F. Gaiti, G. G. Inghirami, V. Adalsteinsson, B. Houck-Loomis, J. Ishii, J. D. Wolchok, G. Boland, N. Robine, N. K. Altorki, D. A. Landau, Genome-wide cell-free DNA mutational integration enables ultra-sensitive cancer monitoring. *Nat. Med.* **26**, 1114–1124 (2020).

12. A. J. Widman, M. Shah, A. Frydendahl, D. Halmos, C. C. Khamnei, N. Øgaard, S. Rajagopalan, A. Arora, A. Deshpande, W. F. Hooper, J. Quentin, J. Bass, M. Zhang, T. Langanay, L. Andersen, Z. Steinsnyder, W. Liao, M. H. Rasmussen, T. V. Henriksen, S. Ø. Jensen, J. Nors, C. Therkildsen, J. Sotelo, R. Brand, J. S. Schiffman, R. H. Shah, A. P. Cheng, C. Maher, L. Spain, K. Krause, D. T. Frederick, W. den Brok, C. Lohrisch, T. Shenkier, C. Simmons, D. Villa, A. J. Mungall, R. Moore, E. Zaikova, V. Cerda, E. Kong, D. Lai, M. S. Malbari, M. Marton, D. Manaa, L. Winterkorn, K. Gelmon, M. K. Callahan, G. Boland, C. Potenski, J. D. Wolchok, A. Saxena, S. Turajlic, M. Imielinski, M. F. Berger, S. Aparicio, N. K. Altorki, M. A. Postow, N. Robine, C. L. Andersen, D. A. Landau, Ultrasensitive plasma-based monitoring of tumor burden using machine-learning-guided signal enrichment. *Nat. Med.* **30**, 1655–1666 (2024).

13. J. R. M. Black, T. Karasaki, C. W. Abbott, B. Li, S. Veeriah, M. Al Bakir, W. K. Liu, A. Huebner, C. Martínez-Ruiz, P. Pawlik, D. A. Moore, D. Marinelli, O. Shutkever, C. Murphy, L. Y. Liu, C. Grieco, K. Grimes, F. C. P. Navarro, R. M. Pyke, G. Bartha, K. C. Keough, S. Dea, N. Ravi, J. Lyle, J. Harris, K. D. Brown, F. H. Blackhall, F. Hassani, D. A. Fennell, N. McGranahan, J. A. Shaw, C. Abbosh, A. Hackshaw, M. Jamal-Hanjani, A. M. Frankell, S. M. Boyle, R. O. Chen, C. Swanton, Longitudinal ultrasensitive ctDNA monitoring for high-resolution lung cancer risk prediction. *Cell* **118**, 7083–7398.e18 (2025).

14. P. Jiang, C. W. M. Chan, K. C. A. Chan, S. H. Cheng, J. Wong, V. W. S. Wong, G. L. H. Wong, S. L. Chan, T. S. K. Mok, H. L. Y. Chan, P. B. S. Lai, R. W. K. Chiu, Y. M. D. Lo, Lengthening and shortening of plasma DNA in hepatocellular carcinoma patients. *Proc. Natl. Acad. Sci. U.S.A.* **112**, E1317–E1325 (2015).

15. F. Mouliere, D. Chandrananda, A. M. Piskorz, E. K. Moore, J. Morris, L. B. Ahlborn, R. Mair, T. Goranova, F. Marass, K. Heider, J. C. M. Wan, A. Supernat, I. Hudecova, I. Gounaris, S. Ros, M. Jimenez-Linan, J. Garcia-Corbacho, K. Patel, O. Østrup, S. Murphy, M. D. Eldridge, D. Gale, G. D. Stewart, J. Burge, W. N. Cooper, M. S. van der Heijden, C. E. Massie, C. Watts, P. Corrie, S. Pacey, K. M. Brindle, R. D. Baird, M. Mau-Sørensen, C. A. Parkinson, C. G. Smith, J. D. Brenton, N. Rosenfeld, Enhanced detection of circulating tumor DNA by fragment size analysis. *Sci. Transl. Med.* **10**, eaat4921 (2018).

16. K. Sun, P. Jiang, S. H. Cheng, T. H. T. Cheng, J. Wong, V. W. S. Wong, S. S. M. Ng, B. B. Y. Ma, T. Y. Leung, S. L. Chan, T. S. K. Mok, P. B. S. Lai, H. L. Y. Chan, H. Sun, K. C. A. Chan, R. W. K. Chiu, Y. M. D. Lo, Orientation-aware plasma cell-free DNA fragmentation analysis in open chromatin regions informs tissue of origin. *Genome Res.* **29**, 418–427 (2019).

17. S. Cristiano, A. Leal, J. Phallen, J. Fiksel, V. Adleff, D. C. Bruhm, S. Ø. Jensen, J. E. Medina, C. Hruban, J. R. White, D. N. Palsgrove, N. Niknafs, V. Anagnostou, P. Forde, J. Naidoo, K. Marrone, J. Brahmer, B. D. Woodward, H. Husain, K. L. van Rooijen, M.-B. W. Ørntoft, A. H. Madsen, C. J. H. van de Velde, M. Verheij, A. Cats, C. J. A. Punt, G. R. Vink, N. C. T. van Grieken, M. Koopman, R. J. A. Fijneman, J. S. Johansen, H. J. Nielsen, G. A. Meijer, C. L.

Andersen, R. B. Scharpf, V. E. Velculescu, Genome-wide cell-free DNA fragmentation in patients with cancer. *Nature* **570**, 385–389 (2019).

18. P. Jiang, K. Sun, W. Peng, S. H. Cheng, M. Ni, P. C. Yeung, M. M. S. Heung, T. Xie, H. Shang, Z. Zhou, R. W. Y. Chan, J. Wong, V. W. S. Wong, L. C. Poon, T. Y. Leung, W. K. J. Lam, J. Y. K. Chan, H. L. Y. Chan, K. C. A. Chan, R. W. K. Chiu, Y. M. D. Lo, Plasma DNA end-motif profiling as a fragmentomic marker in cancer, pregnancy, and transplantation. *Cancer Discov.* **10**, 664–673 (2020).

19. Q. Zhou, G. Kang, P. Jiang, R. Qiao, W. K. J. Lam, S. C. Y. Yu, M. L. Ma, L. Ji, S. H. Cheng, W. Gai, W. Peng, H. Shang, R. W. Y. Chan, S. L. Chan, G. L. H. Wong, L. T. Hiraki, S. Volpi, V. W. S. Wong, J. Wong, R. W. K. Chiu, K. C. A. Chan, Y. M. D. Lo, Epigenetic analysis of cell-free DNA by fragmentomic profiling. *Proc. Natl. Acad. Sci. U.S.A.* **119**, e2209852119 (2022).

20. K. K. Budhraja, B. R. McDonald, M. D. Stephens, T. Contente-Cuomo, H. Markus, M. Farooq, P. F. Favaro, S. Connor, S. A. Byron, J. B. Egan, B. Ernst, T. K. McDaniel, A. Sekulic, N. L. Tran, M. D. Prados, M. J. Borad, M. E. Berens, B. A. Pockaj, P. M. LoRusso, A. Bryce, J. M. Trent, M. Murtaza, Genome-wide analysis of aberrant position and sequence of plasma DNA fragment ends in patients with cancer. *Sci. Transl. Med.* **15**, eabm6863 (2023).

21. N. Moldovan, Y. van der Pol, T. van den Ende, D. Boers, S. Verkuijlen, A. Creemers, J. Ramaker, T. Vu, S. Bootsma, K. J. Lenos, L. Vermeulen, M. F. Fransen, M. Pegtel, I. Bahce, H. van Laarhoven, F. Mouliere, Multi-modal cell-free DNA genomic and fragmentomic patterns enhance cancer survival and recurrence analysis. *Cell Rep. Med.* **5**, 101349 (2024).

22. M.-J. L. Ma, H. Zhang, P. Jiang, S. T. K. Sin, W. K. J. Lam, S. H. Cheng, W.-S. Lee, W. Gai, O. Y. O. Tse, W. Peng, J. Wong, R. Raghupathy, R. S. M. Wong, D. Sahota, T. Y. Leung, K. C. A. Chan, R. W. K. Chiu, Y. M. D. Lo, Topologic analysis of plasma mitochondrial DNA reveals the coexistence of both linear and circular molecules. *Clin. Chem.* **65**, 1161–1170 (2019).

23. P. Burnham, M. S. Kim, S. Agbor-Enoh, H. Luikart, H. A. Valantine, K. K. Khush, I. De Vlaminck, Single-stranded DNA library preparation uncovers the origin and diversity of ultrashort cell-free DNA in plasma. *Sci. Rep.* **6**, 27859 (2016).

24. M. W. Snyder, M. Kircher, A. J. Hill, R. M. Daza, J. Shendure, Cell-free DNA comprises an in vivo nucleosome footprint that informs its tissues-of-origin. *Cell* **164**, 57–68 (2016).

25. P. Ulz, G. G. Thallinger, M. Auer, R. Graf, K. Kashofer, S. W. Jahn, L. Abete, G. Pristauz, E. Petru, J. B. Geigl, E. Heitzer, M. R. Speicher, Inferring expressed genes by whole-genome sequencing of plasma DNA. *Nat. Genet.* **48**, 1273–1278 (2016).

26. P. Ulz, S. Perakis, Q. Zhou, T. Moser, J. Belic, I. Lazzeri, A. Wölfler, A. Zebisch, A. Gerger, G. Pristauz, E. Petru, B. White, C. E. S. Roberts, J. St. John, M. G. Schimek, J. B. Geigl, T. Bauernhofer, H. Sill, C. Bock, E. Heitzer, M. R. Speicher, Inference of transcription factor binding from cell-free DNA enables tumor subtype prediction and early detection. *Nat. Commun.* **10**, 4666 (2019).

27. P. Peneder, A. M. Stutz, D. Surdez, M. Krumbholz, S. Semper, M. Chicard, N. C. Sheffield, G. Pierron, E. Lapouble, M. Totzl, B. Erguner, D. Barreca, A. F. Rendeiro, A. Agaimy, H. Boztug, G. Engstler, M. Dworzak, M. Bernkopf, S. Taschner-Mandl, I. M. Ambros, O. Myklebost, P. Marec-Berard, S. A. Burchill, B. Brennan, S. J. Strauss, J. Whelan, G. Schleiermacher, C. Schaefer, U. Dirksen, C. Hutter, K. Boye, P. F. Ambros, O. Delattre, M. Metzler, C. Bock, E. M. Tomazou, Multimodal analysis of cell-free DNA whole-genome sequencing for pediatric cancers with low mutational burden. *Nat. Commun.* **12**, 3230 (2021).

28. M. S. Esfahani, E. G. Hamilton, M. Mehrmohamadi, B. Y. Nabet, S. K. Alig, D. A. King, C. B. Steen, C. W. Macaulay, A. Schultz, M. C. Nesselbush, J. Soo, J. G. Schroers-Martin, B. Chen, M. S. Binkley, H. Stehr, J. J. Chabon, B. J. Sworder, A. B.-Y. Hui, M. J. Frank, E. J. Moding, C. L. Liu, A. M. Newman, J. M. Isbell, C. M. Rudin, B. T. Li, D. M. Kurtz, M. Diehn, A. A. Alizadeh, Inferring gene expression from cell-free DNA fragmentation profiles. *Nat. Biotechnol.* **40**, 585–597 (2022).

29. L. R. Olsen, D. Odinokov, J. Q. Holsting, K. Kondrup, L. Iisager, M. Rusan, S. Buus, B. E. Laursen, M. Borre, M. R. Jochumsen, K. Bouchelouche, A. Frydendahl, M. H. Rasmussen, T.

V. Henriksen, M. Nesic, C. Demuth, S. V. Lindskrog, I. Nordentoft, P. Lamy, C. Therkildsen, L. Dyrskjøt, K. D. Sørensen, C. L. Andersen, A. Jakobsen Skanderup, S. Besenbacher, Cross-dataset pan-cancer detection by correlating cell-free DNA fragment coverage with open chromatin sites across cell types. *Nat. Commun.* **16**, 11522 (2025).

30. C. Douville, J. D. Cohen, J. Ptak, M. Popoli, J. Schaefer, N. Silliman, L. Dobbyn, R. E. Schoen, J. Tie, P. Gibbs, M. Goggins, C. L. Wolfgang, T. L. Wang, I. M. Shih, R. Karchin, A. M. Lennon, R. H. Hruban, C. Tomasetti, C. Bettegowda, K. W. Kinzler, N. Papadopoulos, B. Vogelstein, Assessing aneuploidy with repetitive element sequencing. *Proc. Natl. Acad. Sci. U.S.A.* **117**, 4858–4863 (2020).

31. C. Douville, K. Lahouel, A. Kuo, H. Grant, B. E. Avigdor, S. D. Curtis, M. Summers, J. D. Cohen, Y. Wang, A. Mattox, J. Dudley, L. Dobbyn, M. Popoli, J. Ptak, N. Nehme, N. Silliman, C. Blair, K. Romans, C. Thoburn, J. Gizzi, R. E. Schoen, J. Tie, P. Gibbs, L. T. Ho-Pham, B. N. H. Tran, T. S. Tran, T. V. Nguyen, M. Goggins, C. L. Wolfgang, T.-L. Wang, I.-M. Shih, A. M. Lennon, R. H. Hruban, C. Bettegowda, K. W. Kinzler, N. Papadopoulos, B. Vogelstein, C. Tomasetti, Machine learning to detect the SINEs of cancer. *Sci. Transl. Med.* **16**, eadi3883 (2024).

32. A. V. Annapragada, N. Niknafs, J. R. White, D. C. Bruhm, C. Cherry, J. E. Medina, V. Adleff, C. Hruban, D. Mathios, Z. H. Foda, J. Phallen, R. B. Scharpf, V. E. Velculescu, Genome-wide repeat landscapes in cancer and cell-free DNA. *Sci. Transl. Med.* **16**, eadj9283 (2024).

33. Y. M. D. Lo, D. S. C. Han, P. Jiang, R. W. K. Chiu, Epigenetics, fragmentomics, and topology of cell-free DNA in liquid biopsies. *Science* **372**, eaaw3616 (2021).

34. A. R. Thierry, Circulating DNA fragmentomics and cancer screening. *Cell Genom.* **3**, 100242 (2023).

35. K. T. Helzer, M. N. Sharifi, J. M. Sperger, Y. Shi, M. Annala, M. L. Bootsma, S. R. Reese, A. Taylor, K. R. Kaufmann, H. K. Krause, J. L. Schehr, N. Sethakorn, D. Kosoff, C. Kyriakopoulos, M. E. Burkard, N. R. Rydzewski, M. Yu, P. M. Harari, M. Bassetti, G. Blitzer, J. Floberg, M. Sjöström, D. A. Quigley, S. M. Dehm, A. J. Armstrong, H. Beltran, R. R. McKay, F. Y. Feng, R. O'Regan, K. B. Wisinski, H. Emamekhoo, A. W. Wyatt, J. M. Lang,

S. G. Zhao, Fragmentomic analysis of circulating tumor DNA-targeted cancer panels. *Ann. Oncol.* **34**, 813–825 (2023).

36. Y. Liu, At the dawn: Cell-free DNA fragmentomics and gene regulation. *Br. J. Cancer* **126**, 379–390 (2022).

37. Y. V. Zhitnyuk, A. P. Koval, A. A. Alferov, Y. A. Shtykova, I. Z. Mamedov, N. E. Kushlinskii, D. M. Chudakov, D. S. Shcherbo, Deep cfDNA fragment end profiling enables cancer detection. *Mol. Cancer* **21**, 26 (2022).

38. R. W. K. Chiu, E. Heitzer, Y. M. D. Lo, F. Mouliere, D. W. Y. Tsui, Cell-free DNA fragmentomics: The new "omics" on the block. *Clin. Chem.* **66**, 1480–1484 (2020).

39. D. S. C. Han, M. Ni, R. W. Y. Chan, V. W. H. Chan, K. O. Lui, R. W. K. Chiu, Y. M. D. Lo, The biology of cell-free DNA fragmentation and the roles of DNASE1, DNASE1L3, and DFFB. *Am. J. Hum. Genet.* **106**, 202–214 (2020).

40. Z. Zhou, M.-J. L. Ma, R. W. Y. Chan, W. K. J. Lam, W. Peng, W. Gai, X. Hu, S. C. Ding, L. Ji, Q. Zhou, P. P. H. Cheung, S. C. Y. Yu, J. Y. C. Teoh, C.-C. Szeto, J. Wong, V. W. S. Wong, G. L. H. Wong, S. L. Chan, E. P. Hui, B. B. Y. Ma, A. T. C. Chan, R. W. K. Chiu, K. C. A. Chan, Y. M. D. Lo, P. Jiang, Fragmentation landscape of cell-free DNA revealed by deconvolutional analysis of end motifs. *Proc. Natl. Acad. Sci.* **120**, e2220982120 (2023).

41. M. Noë, D. Mathios, A. V. Annapragada, S. Koul, Z. H. Foda, J. E. Medina, S. Cristiano, C. Cherry, D. C. Bruhm, N. Niknafs, V. Adleff, L. Ferreira, H. Easwaran, S. Baylin, J. Phallen, R. B. Scharpf, V. E. Velculescu, DNA methylation and gene expression as determinants of genome-wide cell-free DNA fragmentation. *Nat. Commun.* **15**, 6690 (2024).

42. E. Heitzer, I. S. Haque, C. E. S. Roberts, M. R. Speicher, Current and future perspectives of liquid biopsies in genomics-driven oncology. *Nat. Rev. Genet.* **20**, 71–88 (2019).

43. S. Steyaert, M. Pizurica, D. Nagaraj, P. Khandelwal, T. Hernandez-Boussard, A. J. Gentles, O. Gevaert, Multimodal data fusion for cancer biomarker discovery with deep learning. *Nat. Mach. Intell.* **5**, 351–362 (2023).

44. A. Tivey, R. J. Lee, A. Clipson, S. M. Hill, P. Lorigan, D. G. Rothwell, C. Dive, F. Mouliere, Mining nucleic acid "omics" to boost liquid biopsy in cancer. *Cell Rep. Med.* **5**, 101736 (2024).

45. Y. LeCun, Y. Bengio, G. Hinton, Deep learning. *Nature* **521**, 436–444 (2015).

46. K. Simonyan, A. Zisserman, Very deep convolutional networks for large-scale image recognition. arXiv arXiv:1409.1556 [cs.CV] (2015). https://doi.org/10.48550/arXiv.1409.1556.

47. K. He, X. Zhang, S. Ren, J. Sun, Deep residual learning for image recognition. arXiv:1512.03385 [cs.CV] (2015); https://doi.org/10.48550/arXiv.1512.03385.

48. A. Vaswani, N. Shazeer, N. Parmar, J. Uszkoreit, L. Jones, A. N. Gomez, L. Kaiser, I. Polosukhin, Attention is all you need. arXiv:1706.03762 [cs.CL] (2023); https://doi.org/10.48550/arXiv.1706.03762.

49. A. Dosovitskiy, L. Beyer, A. Kolesnikov, D. Weissenborn, X. Zhai, T. Unterthiner, M. Dehghani, M. Minderer, G. Heigold, S. Gelly, J. Uszkoreit, N. Houlsby, An image is worth 16x16 words: Transformers for image recognition at scale. arXiv:2010.11929 [cs.CV] (2021); https://doi.org/10.48550/arXiv.2010.11929.

50. R. Perez-Lopez, N. Ghaffari Laleh, F. Mahmood, J. N. Kather, A guide to artificial intelligence for cancer researchers. *Nat. Rev. Cancer* **24**, 427–441 (2024).

51. M. Gehrung, M. Crispin-Ortuzar, A. G. Berman, M. O'Donovan, R. C. Fitzgerald, F. Markowetz, Triage-driven diagnosis of Barrett's esophagus for early detection of esophageal adenocarcinoma using deep learning. *Nat. Med.* **27**, 833–841 (2021).

52. H. Dalla-Torre, L. Gonzalez, J. Mendoza-Revilla, A. H. Grzywaczewski, F. Oteri, C. Dallago, E. Trop, B. P. de Almeida, H. Sirelkhatim, G. Richard, M. Skwark, K. Beguir, M. Lopez, T. Pierrot, Nucleotide transformer: Building and evaluating robust foundation models for human genomics. *Nat. Methods* **22**, 287–297 (2025).

53. J. Li, Z. Guan, J. Wang, C. Y. Cheung, Y. Zheng, L.-L. Lim, C. C. Lim, P. Ruamviboonsuk, R. Raman, L. Corsino, J. B. Echouffo-Tcheugui, A. O. Y. Luk, L. J. Chen, X. Sun, H.

Hamzah, Q. Wu, X. Wang, R. Liu, Y. X. Wang, T. Chen, X. Zhang, X. Yang, J. Yin, J. Wan, W. Du, T. C. Quek, J. H. L. Goh, D. Yang, X. Hu, T. X. Nguyen, S. K. H. Szeto, P. Chotcomwongse, R. Malek, N. Normatova, N. Ibragimova, R. Srinivasan, P. Zhong, W. Huang, C. Deng, L. Ruan, C. Zhang, C. Zhang, Y. Zhou, C. Wu, R. Dai, S. W. C. Koh, A. Abdullah, N. K. Y. Hee, H. C. Tan, Z. H. Liew, C. S.-Y. Tien, S. L. Kao, A. Y. L. Lim, S. F. Mok, L. Sun, J. Gu, L. Wu, T. Li, D. Cheng, Z. Wang, Y. Qin, L. Dai, Z. Meng, J. Shu, Y. Lu, N. Jiang, T. Hu, S. Huang, G. Huang, S. Yu, D. Liu, W. Ma, M. Guo, X. Guan, X. Yang, C. Bascaran, C. R. Cleland, Y. Bao, E. I. Ekinci, A. Jenkins, J. C. N. Chan, Y. M. Bee, S. Sivaprasad, J. E. Shaw, R. Simó, P. A. Keane, C.-Y. Cheng, G. S. W. Tan, W. Jia, Y.-C. Tham, H. Li, B. Sheng, T. Y. Wong, Integrated image-based deep learning and language models for primary diabetes care. *Nat. Med.* **30**, 2886–2896 (2024).

54. M. W. Mullowney, K. R. Duncan, S. S. Elsayed, N. Garg, J. J. J. van der Hooft, N. I. Martin, D. Meijer, B. R. Terlouw, F. Biermann, K. Blin, J. Durairaj, M. Gorostiola González, E. J. N. Helfrich, F. Huber, S. Leopold-Messer, K. Rajan, T. de Rond, J. A. van Santen, M. Sorokina, M. J. Balunas, M. A. Beniddir, D. A. van Bergeijk, L. M. Carroll, C. M. Clark, D.-A. Clevert, C. A. Dejong, C. Du, S. Ferrinho, F. Grisoni, A. Hofstetter, W. Jespers, O. V. Kalinina, S. A. Kautsar, H. Kim, T. F. Leao, J. Masschelein, E. R. Rees, R. Reher, D. Reker, P. Schwaller, M. Segler, M. A. Skinnider, A. S. Walker, E. L. Willighagen, B. Zdrazil, N. Ziemert, R. J. M. Goss, P. Guyomard, A. Volkamer, W. H. Gerwick, H. U. Kim, R. Müller, G. P. van Wezel, G. J. P. van Westen, A. K. H. Hirsch, R. G. Linington, S. L. Robinson, M. H. Medema, Artificial intelligence for natural product drug discovery. *Nat. Rev. Drug Discov.* **22**, 895–916 (2023).

55. C. Cortes, V. Vapnik, Support-vector networks. *Mach. Learn.* **20**, 273–297 (1995).

56. D. R. Cox, The regression analysis of binary sequences. *J. R. Stat. Soc. B. Methodol.* **20**, 215–232 (1958).

57. H. Zheng, M. S. Zhu, Y. Liu, FinaleDB: A browser and database of cell-free DNA fragmentation patterns. *Bioinformatics* **37**, 2502–2503 (2021).

58. V. A. Adalsteinsson, G. Ha, S. S. Freeman, A. D. Choudhury, D. G. Stover, H. A. Parsons, G. Gydush, S. C. Reed, D. Rotem, J. Rhoades, D. Loginov, D. Livitz, D. Rosebrock, I. Leshchiner, J. Kim, C. Stewart, M. Rosenberg, J. M. Francis, C. Z. Zhang, O. Cohen, C. Oh,

H. Ding, P. Polak, M. Lloyd, S. Mahmud, K. Helvie, M. S. Merrill, R. A. Santiago, E. P. O'Connor, S. H. Jeong, R. Leeson, R. M. Barry, J. F. Kramkowski, Z. Zhang, L. Polacek, J. G. Lohr, M. Schleicher, E. Lipscomb, A. Saltzman, N. M. Oliver, L. Marini, A. G. Waks, L. C. Harshman, S. M. Tolaney, E. M. Van Allen, E. P. Winer, N. U. Lin, M. Nakabayashi, M. E. Taplin, C. M. Johannessen, L. A. Garraway, T. R. Golub, J. S. Boehm, N. Wagle, G. Getz, J. C. Love, M. Meyerson, Scalable whole-exome sequencing of cell-free DNA reveals high concordance with metastatic tumors. *Nat. Commun.* **8**, 1324 (2017).

59. S. M. Lundberg, S.-I. Lee, "A unified approach to interpreting model predictions" in *Advances in Neural Information Processing Systems*, I. Guyon, U. V. Luxburg, S. Bengio, H. Wallach, R. Fergus, S. Vishwanathan, R. Garnett, Eds. (Curran Associates Inc., 2017), vol. 30; arXiv:1705.07874 [cs.AI] https://doi.org/10.48550/arXiv.1705.07874.

60. M. Tan, Q. V. Le, EfficientNet: Rethinking model scaling for convolutional neural networks. arXiv:1905.11946 [cs.LG] (2020); https://doi.org/10.48550/arXiv.1905.11946.

61. J. J. Chabon, E. G. Hamilton, D. M. Kurtz, M. S. Esfahani, E. J. Moding, H. Stehr, J. Schroers-Martin, B. Y. Nabet, B. Chen, A. A. Chaudhuri, C. L. Liu, A. B. Hui, M. C. Jin, T. D. Azad, D. Almanza, Y.-J. Jeon, M. C. Nesselbush, L. Co Ting Keh, R. F. Bonilla, C. H. Yoo, R. B. Ko, E. L. Chen, D. J. Merriott, P. P. Massion, A. S. Mansfield, J. Jen, H. Z. Ren, S. H. Lin, C. L. Costantino, R. Burr, R. Tibshirani, S. S. Gambhir, G. J. Berry, K. C. Jensen, R. B. West, J. W. Neal, H. A. Wakelee, B. W. Loo, C. A. Kunder, A. N. Leung, N. S. Lui, M. F. Berry, J. B. Shrager, V. S. Nair, D. A. Haber, L. V. Sequist, A. A. Alizadeh, M. Diehn, Integrating genomic features for non-invasive early lung cancer detection. *Nature* **580**, 245–251 (2020).

62. D. Mathios, J. S. Johansen, S. Cristiano, J. E. Medina, J. Phallen, K. R. Larsen, D. C. Bruhm, N. Niknafs, L. Ferreira, V. Adleff, J. Y. Chiao, A. Leal, M. Noe, J. R. White, A. S. Arun, C. Hruban, A. V. Annapragada, S. Ø. Jensen, M.-B. W. Ørntoft, A. H. Madsen, B. Carvalho, M. de Wit, J. Carey, N. C. Dracopoli, T. Maddala, K. C. Fang, A.-R. Hartman, P. M. Forde, V. Anagnostou, J. R. Brahmer, R. J. A. Fijneman, H. J. Nielsen, G. A. Meijer, C. L. Andersen, A. Mellemgaard, S. E. Bojesen, R. B. Scharpf, V. E. Velculescu, Detection and characterization of lung cancer using cell-free DNA fragmentomes. *Nat. Commun.* **12**, 5060 (2021).

63. B. Budnik, H. Amirkhani, M. H. Forouzanfar, A. Afshin, Novel proteomics-based plasma test for early detection of multiple cancers in the general population. *BMJ Oncol.* **3**, e000073 (2024).

64. N. Loyfer, J. Magenheim, A. Peretz, G. Cann, J. Bredno, A. Klochendler, I. Fox-Fisher, S. Shabi-Porat, M. Hecht, T. Pelet, J. Moss, Z. Drawshy, H. Amini, P. Moradi, S. Nagaraju, D. Bauman, D. Shveiky, S. Porat, U. Dior, G. Rivkin, O. Or, N. Hirshoren, E. Carmon, A. Pikarsky, A. Khalaileh, G. Zamir, R. Grinbaum, M. Abu Gazala, I. Mizrahi, N. Shussman, A. Korach, O. Wald, U. Izhar, E. Erez, V. Yutkin, Y. Samet, D. Rotnemer Golinkin, K. L. Spalding, H. Druid, P. Arner, A. M. J. Shapiro, M. Grompe, A. Aravanis, O. Venn, A. Jamshidi, R. Shemer, Y. Dor, B. Glaser, T. Kaplan, A DNA methylation atlas of normal human cell types. *Nature* **613**, 355–364 (2023).

65. A.-M. Conway, S. P. Pearce, A. Clipson, S. M. Hill, F. Chemi, D. Slane-Tan, S. Ferdous, A. S. M. M. Hossain, K. Kamieniecka, D. J. White, C. Mitchell, A. Kerr, M. G. Krebs, G. Brady, C. Dive, N. Cook, D. G. Rothwell, A cfDNA methylation-based tissue-of-origin classifier for cancers of unknown primary. *Nat. Commun.* **15**, 3292 (2024).

66. S. J. Dawson, D. W. Tsui, M. Murtaza, H. Biggs, O. M. Rueda, S. F. Chin, M. J. Dunning, D. Gale, T. Forshew, B. Mahler-Araujo, S. Rajan, S. Humphray, J. Becq, D. Halsall, M. Wallis, D. Bentley, C. Caldas, N. Rosenfeld, Analysis of circulating tumor DNA to monitor metastatic breast cancer. *N. Engl. J. Med.* **368**, 1199–1209 (2013).

67. J. C. M. Wan, D. Stephens, L. Luo, J. R. White, C. M. Stewart, B. Rousseau, D. W. Y. Tsui, L. A. Diaz, Genome-wide mutational signatures in low-coverage whole genome sequencing of cell-free DNA. *Nat. Commun.* **13**, 4953 (2022).

68. D. C. Bruhm, D. Mathios, Z. H. Foda, A. V. Annapragada, J. E. Medina, V. Adleff, E. J. Chiao, L. Ferreira, S. Cristiano, J. R. White, S. A. Mazzilli, E. Billatos, A. Spira, A. H. Zaidi, J. Mueller, A. K. Kim, V. Anagnostou, J. Phallen, R. B. Scharpf, V. E. Velculescu, Single-molecule genome-wide mutation profiles of cell-free DNA for non-invasive detection of cancer. *Nat. Genet.* **55**, 1301–1310 (2023).

69. M. Kowarsky, J. Camunas-Soler, M. Kertesz, I. De Vlaminck, W. Koh, W. Pan, L. Martin, N. F. Neff, J. Okamoto, R. J. Wong, S. Kharbanda, Y. El-Sayed, Y. Blumenfeld, D. K. Stevenson, G. M. Shaw, N. D. Wolfe, S. R. Quake, Numerous uncharacterized and highly divergent microbes which colonize humans are revealed by circulating cell-free DNA. *Proc. Natl. Acad. Sci. U.S.A.* **114**, 9623–9628 (2017).

70. K. Heider, J. C. M. Wan, J. Hall, J. Belic, S. Boyle, I. Hudecova, D. Gale, W. N. Cooper, P. G. Corrie, J. D. Brenton, C. G. Smith, N. Rosenfeld, Detection of ctDNA from dried blood spots after DNA size selection. *Clin. Chem.* **66**, 697–705 (2020).

71. L. Raman, M. Van der Linden, K. Van der Eecken, K. Vermaelen, I. Demedts, V. Surmont, U. Himpe, F. Dedeurwaerdere, L. Ferdinande, Y. Lievens, K. Claes, B. Menten, J. Van Dorpe, Shallow whole-genome sequencing of plasma cell-free DNA accurately differentiates small from non-small cell lung carcinoma. *Genome Med.* **12**, 35 (2020).

72. R. M. Drews, B. Hernando, M. Tarabichi, K. Haase, T. Lesluyes, P. S. Smith, L. Morrill Gavarró, D.-L. Couturier, L. Liu, M. Schneider, J. D. Brenton, P. Van Loo, G. Macintyre, F. Markowetz, A pan-cancer compendium of chromosomal instability. *Nature* **606**, 976–983 (2022).

73. A. Hallermayr, T. Wohlfrom, V. Steinke-Lange, A. Benet-Pagès, F. Scharf, E. Heitzer, U. Mansmann, C. Haberl, M. de Wit, H. Vogelsang, M. Rentsch, E. Holinski-Feder, J. M. A. Pickl, Somatic copy number alteration and fragmentation analysis in circulating tumor DNA for cancer screening and treatment monitoring in colorectal cancer patients. *J. Hematol. Oncol.* **15**, 125 (2022).

74. W. Qiang, L. Jin, T. Luo, Y. Jia, J. Lu, J. Liu, H. He, Z. Qian, S. Mithraprabhu, Y. Liang, R. P. Gale, X. Tao, D. Wu, J. Du, Cell-free DNA chromosome copy number variations predict outcomes in plasma cell myeloma. *Blood Cancer J.* **13**, 136 (2023).

75. Y. Wang, K. Zhou, X. Wang, Y. Liu, D. Guo, Z. Bian, L. Su, K. Liu, X. Gu, X. Guo, L. Wang, H. Zhang, K. Tao, J. Xing, Multiple-level copy number variations in cell-free DNA for prognostic prediction of HCC with radical treatments. *Cancer Sci.* **112**, 4772–4784 (2021).

76. F. Mouliere, R. Mair, D. Chandrananda, F. Marass, C. G. Smith, J. Su, J. Morris, C. Watts, K. M. Brindle, N. Rosenfeld, Detection of cell-free DNA fragmentation and copy number alterations in cerebrospinal fluid from glioma patients. *EMBO Mol. Med.* **10**, (2018).

77. J. C. M. Wan, P. Sasieni, N. Rosenfeld, Promises and pitfalls of multi-cancer early detection using liquid biopsy tests. *Nat. Rev. Clin. Oncol.* **22**, 566–580 (2025).

78. Y. Xu, S. Zhu, C. Xia, H. Yu, S. Shi, K. Chen, Y. He, C. Deng, H. Jin, J. Liu, R. Fitzgerald, P. Basu, W. Chen, Liquid biopsy-based multi-cancer early detection: An exploration road from evidence to implementation. *Sci. Bull.* **70**, 2852–2867 (2025).

79. L. C. Kahwati, M. Avenarius, L. Brouwer, N. L. Crossnohere, C. A. Doubeni, C. Miller, M. Siddiqui, C. Voisin, R. C. Wines, D. E. Jonas, Multicancer detection tests for screening. *Ann. Intern. Med.* **178**, 1591–1604 (2025).

80. C. M. Sauer, M. D. Eldridge, M. Vias, J. A. Hall, S. Boyle, G. Macintyre, T. Bradley, F. Markowetz, J. D. Brenton, Absolute copy number fitting from shallow whole genome sequencing data. bioRxiv, 2021.07.19.452658 [Preprint] (2021); https://doi.org/10.1101/2021.07.19.452658.

81. C. M. Sauer, K. Heider, J. Belic, S. E. Boyle, J. A. Hall, D. Couturier, A. An, A. Vijayaraghavan, M. A. Reinius, K. Hosking, M. Vias, N. Rosenfeld, J. D. Brenton, Longitudinal monitoring of disease burden and response using ctDNA from dried blood spots in xenograft models. *EMBO Mol. Med.* **14**, e15729 (2022).

82. D. Gale, K. Heider, A. Ruiz-Valdepenas, S. Hackinger, M. Perry, G. Marsico, V. Rundell, J. Wulff, G. Sharma, H. Knock, J. Castedo, W. Cooper, H. Zhao, C. G. Smith, S. Garg, S. Anand, K. Howarth, D. Gilligan, S. V. Harden, D. M. Rassl, R. C. Rintoul, N. Rosenfeld, Residual ctDNA after treatment predicts early relapse in patients with early-stage non-small cell lung cancer. *Ann. Oncol.* **33**, 500–510 (2022).

83. M. M. F. Schuurbiers, C. G. Smith, K. J. Hartemink, R. C. Rintoul, D. Gale, K. Monkhorst, B. L. R. Mandos, A. L. Paterson, D. van den Broek, N. Rosenfeld, M. M. van den Heuvel, LEMA Study Group and the LUCID Study Group, Recurrence prediction using circulating

tumor DNA in patients with early-stage non-small cell lung cancer after treatment with curative intent: A retrospective validation study. *PLOS Med.* **22**, e1004574 (2025).

84. S. V. Lightowlers, A. Machin, R. Woitek, E. Provenzano, I. Allajbeu, W. A. Sarakbi, N. Demiris, P. Forouhi, F. J. Gilbert, A. M. Kirby, C. Towns, N. Somaiah, C. E. Coles, Neoadjuvant radiotherapy and endocrine therapy for oestrogen receptor positive breast cancers: The Neo-RT feasibility study. *Clin. Oncol.* **37**, 103669 (2025).

85. A. Pearson, P. Proszek, J. Pascual, C. Fribbens, M. K. Shamsher, B. Kingston, B. O'Leary, M. T. Herrera-Abreu, R. J. Cutts, I. Garcia-Murillas, H. Bye, B. A. Walker, D. Gonzalez De Castro, L. Yuan, S. Jamal, M. Hubank, E. Lopez-Knowles, E. F. Schuster, M. Dowsett, P. Osin, A. Nerurkar, M. Parton, A. F. C. Okines, S. R. D. Johnston, A. Ring, N. C. Turner, Inactivating NF1 mutations are enriched in advanced breast cancer and contribute to endocrine therapy resistance. *Clin. Cancer Res.* **26**, 608–622 (2020).

86. I. Garcia-Murillas, N. Chopra, I. Comino-Méndez, M. Beaney, H. Tovey, R. J. Cutts, C. Swift, D. Kriplani, M. Afentakis, S. Hrebien, G. Walsh-Crestani, P. Barry, S. R. D. Johnston, A. Ring, J. Bliss, S. Russell, A. Evans, A. Skene, D. Wheatley, M. Dowsett, I. E. Smith, N. C. Turner, Assessment of molecular relapse detection in early-stage breast cancer. *JAMA Oncol.* **5**, 1473–1478 (2019).

87. H. Wang, P. D. Mennea, Y. K. E. Chan, Z. Cheng, M. C. Neofytou, A. A. Surani, A. Vijayaraghavan, E.-J. Ditter, R. Bowers, M. D. Eldridge, D. S. Shcherbo, C. G. Smith, F. Markowetz, W. N. Cooper, T. Kaplan, N. Rosenfeld, H. Zhao, A standardized framework for robust fragmentomic feature extraction from cell-free DNA sequencing data. *Genome Biol.* **26**, 141 (2025).

88. A. Santonja, W. N. Cooper, M. D. Eldridge, P. A. W. Edwards, J. A. Morris, A. R. Edwards, H. Zhao, K. Heider, D.-L. Couturier, A. Vijayaraghavan, P. Mennea, E.-J. Ditter, C. G. Smith, C. Boursnell, R. M. García, O. M. Rueda, E. Beddowes, H. Biggs, S.-J. Sammut, N. Rosenfeld, C. Caldas, J. E. Abraham, D. Gale, Comparison of tumor-informed and tumor-naïve sequencing assays for ctDNA detection in breast cancer. *EMBO Mol. Med.* **15**, e16505 (2023).

89. S. Lee, D. Cook, M. Lawrence, Plyranges: A grammar of genomic data transformation. *Genome Biol.* **20**, 4 (2019).

90. I. Scheinin, D. Sie, H. Bengtsson, M. A. van de Wiel, A. B. Olshen, H. F. van Thuijl, H. F. van Essen, P. P. Eijk, F. Rustenburg, G. A. Meijer, J. C. Reijneveld, P. Wesseling, D. Pinkel, D. G. Albertson, B. Ylstra, DNA copy number analysis of fresh and formalin-fixed specimens by shallow whole-genome sequencing with identification and exclusion of problematic regions in the genome assembly. *Genome Res.* **24**, 2022–2032 (2014).

91. K. Xiong, J. Ma, Revealing Hi-C subcompartments by imputing inter-chromosomal chromatin interactions. *Nat. Commun.* **10**, 5069 (2019).

92. T. Derrien, J. Estellé, S. M. Sola, D. G. Knowles, E. Raineri, R. Guigó, P. Ribeca, Fast computation and applications of genome mappability. *PLOS ONE* **7**, e30377 (2012).

93. H. M. Amemiya, A. Kundaje, A. P. Boyle, The ENCODE blacklist: Identification of problematic regions of the genome. *Sci. Rep.* **9**, 9354 (2019).

94. M. Ramos, L. Schiffer, A. Re, R. Azhar, A. Basunia, C. Rodriguez, T. Chan, P. Chapman, S. R. Davis, D. Gomez-Cabrero, A. C. Culhane, B. Haibe-Kains, K. D. Hansen, H. Kodali, M. S. Louis, A. S. Mer, M. Riester, M. Morgan, V. Carey, L. Waldron, Software for the integration of multiomics experiments in bioconductor. *Cancer Res.* **77**, e39–e42 (2017).

95. V. E. Seshan, A. B. Olshen, DNAcopy : A package for analyzing DNA copy data. *Bioconductor Vignette,* (2014) 10.18129/B9.bioc.DNAcopy.

96. J. Grimwood, L. A. Gordon, A. Olsen, A. Terry, J. Schmutz, J. Lamerdin, U. Hellsten, D. Goodstein, O. Couronne, M. Tran-Gyamfi, A. Aerts, M. Altherr, L. Ashworth, E. Bajorek, S. Black, E. Branscomb, S. Caenepeel, A. Carrano, C. Caoile, Y. Man Chan, M. Christensen, C. A. Cleland, A. Copeland, E. Dalin, P. Dehal, M. Denys, J. C. Detter, J. Escobar, D. Flowers, D. Fotopulos, C. Garcia, A. M. Georgescu, T. Glavina, M. Gomez, E. Gonzales, M. Groza, N. Hammon, T. Hawkins, L. Haydu, I. Ho, W. Huang, S. Israni, J. Jett, K. Kadner, H. Kimball, A. Kobayashi, V. Larionov, S.-H. Leem, F. Lopez, Y. Lou, S. Lowry, S. Malfatti, D. Martinez, P. McCready, C. Medina, J. Morgan, K. Nelson, M. Nolan, I. Ovcharenko, S. Pitluck, M.

Pollard, A. P. Popkie, P. Predki, G. Quan, L. Ramirez, S. Rash, J. Retterer, A. Rodriguez, S. Rogers, A. Salamov, A. Salazar, X. She, D. Smith, T. Slezak, V. Solovyev, N. Thayer, H. Tice, M. Tsai, A. Ustaszewska, N. Vo, M. Wagner, J. Wheeler, K. Wu, G. Xie, J. Yang, I. Dubchak, T. S. Furey, P. DeJong, M. Dickson, D. Gordon, E. E. Eichler, L. A. Pennacchio, P. Richardson, L. Stubbs, D. S. Rokhsar, R. M. Myers, E. M. Rubin, S. M. Lucas, The DNA sequence and biology of human chromosome 19. *Nature* **428**, 529–535 (2004).

97. R. A. Harris, M. Raveendran, K. C. Worley, J. Rogers, Unusual sequence characteristics of human chromosome 19 are conserved across 11 nonhuman primates. *BMC Evol. Biol.* **20**, 33 (2020).

98. R. Straver, E. A. Sistermans, H. Holstege, A. Visser, C. B. M. Oudejans, M. J. T. Reinders, WISECONDOR: Detection of fetal aberrations from shallow sequencing maternal plasma based on a within-sample comparison scheme. *Nucleic Acids Res.* **42**, e31 (2014).

99. T. Gerber, S. Taschner-Mandl, L. Saloberger-Sindhöringer, N. Popitsch, E. Heitzer, V. Witt, R. Geyeregger, C. Hutter, R. Schwentner, I. M. Ambros, P. F. Ambros, Assessment of pre-analytical sample handling conditions for comprehensive liquid biopsy analysis. *J. Mol. Diagn.* **22**, 1070–1086 (2020).

100. X. Hu, H. Zhang, Y. Wang, Y. Lin, Q. Li, L. Li, G. Zeng, R. Ou, X. Cheng, Y. Zhang, X. Jin, Effects of blood-processing protocols on cell-free DNA fragmentomics in plasma: Comparisons of one- and two-step centrifugations. *Clin. Chim. Acta* **560**, 119729 (2024).

101. S. K. Terp, I. S. Pedersen, M. P. Stoico, Extraction of cell-free DNA: Evaluation of efficiency, quantity, and quality. *J. Mol. Diagn.* **26**, 310–319 (2024).

102. R Core Team, *R: A Language and Environment for Statistical Computing* (R Foundation for Statistical Computing, Vienna, Austria, 2022); https://R-project.org/.

103. A. Kassambara, F. Mundt, *Factoextra: Extract and Visualize the Results of Multivariate Data Analyses* (2020); https://CRAN.R-project.org/package=factoextra.

104. F. Pedregosa, G. Varoquaux, A. Gramfort, V. Michel, B. Thirion, O. Grisel, M. Blondel, P. Prettenhofer, R. Weiss, V. Dubourg, J. Vanderplas, A. Passos, D. Cournapeau, M. Brucher, M. Perrot, E. Duchesnay, Scikit-learn: Machine Learning in Python. *J. Mach. Learn. Res.* **12**, 2825–2830 (2011).

105. T. Chen, C. Guestrin, "XGBoost: A scalable tree boosting system" in *Proceedings of the 22nd ACM SIGKDD International Conference on Knowledge Discovery and Data Mining* (ACM, 2016); https://dl.acm.org/doi/10.1145/2939672.2939785, pp. 785–794.

106. S. M. Lundberg, G. Erion, H. Chen, A. DeGrave, J. M. Prutkin, B. Nair, R. Katz, J. Himmelfarb, N. Bansal, S.-I. Lee, From local explanations to global understanding with explainable AI for trees. *Nat. Mach. Intell.* **2**, 56–67 (2020).

107. G. Van Rossum, F. L. Drake, *Python 3 Reference Manual* (CreateSpace, 2009).

108. G. M. Kurtzer, V. Sochat, M. W. Bauer, Singularity: Scientific containers for mobility of compute. *PLOS ONE* **12**, e0177459 (2017).

109. L. Paracchini, L. Beltrame, T. Grassi, A. Inglesi, R. Fruscio, F. Landoni, D. Ippolito, M. Delle Marchette, M. Paderno, M. Adorni, M. Jaconi, C. Romualdi, M. D'Incalci, G. Siravegna, S. Marchini, Genome-wide copy-number alterations in circulating tumor DNA as a novel biomarker for patients with high-grade serous ovarian cancer. *Clin. Cancer Res.* **27**, 2549–2559 (2021).

110. Picard2019toolkit, Picard toolkit, 2020 (2019); http://broadinstitute.github.io/picard/.

111. G. van der Auwera, B. O'Connor, *Genomics in the cloud : using Docker, GATK, and WDL in Terra* (O'Reilly Media, ed. 1, 2020).

112. A. M. Bolger, M. Lohse, B. Usadel, Trimmomatic: A flexible trimmer for Illumina sequence data. *Bioinformatics* **30**, 2114–2120 (2014).

113. H. Li, Aligning sequence reads, clone sequences and assembly contigs with BWA-MEM. arXiv:1303.3997 [q-bio.GN] (2013); https://doi.org/10.48550/arXiv.1303.3997.

114. G. G. Faust, I. M. Hall, SAMBLASTER: Fast duplicate marking and structural variant read extraction, in *Bioinformatics* (2014), vol. 30.

115. M. Morgan, L. Shepherd, AnnotationHub: Client to access AnnotationHub resources, Bioconductor (2022). 10.18129/B9.bioc.AnnotationHub.
